# Genetic dissection of the fuzzless seed trait in *Gossypium barbadense*

**DOI:** 10.1093/jxb/erx459

**Published:** 2018-01-17

**Authors:** Qian-Hao Zhu, Yuman Yuan, Warwick Stiller, Yinhua Jia, Pengpeng Wang, Zhaoe Pan, Xiongming Du, Danny Llewellyn, Iain Wilson

**Affiliations:** 1 CSIRO Agriculture and Food, Canberra, ACT, Australia; 2 CSIRO Agriculture and Food, Locked, Narrabri, NSW, Australia; 3 State Key Laboratory of Cotton Biology/Institute of Cotton Research, Chinese Academy of Agricultural Sciences, Anyang, Henan, China

**Keywords:** Bulk segregant analysis, fuzzless, fuzz percentage, *G. barbadense*, lintless, mapping-by-sequencing

## Abstract

Cotton fibres are single-celled trichomes arising from the epidermal cells of the seed coat and may be either long (lint) or very short (fuzz). The dominant fuzzless *N*_*1*_ of *Gossypium hirsutum* is a defective allele of the At-subgenome homoeolog of *MYB25-like*, but the genetic components underlying the recessive fuzzless trait from *G. barbadense* (Gb) are unknown. We have identified five genetic loci, including a major contributing locus containing *MYB25-like_Dt*, associated with Gb fuzzless seeds based on genotyping of fuzzy and fuzzless near isogenic lines (NILs) from an interspecies cross (*G. barbadense* × *G. hirsutum*). At 3 d post-anthesis when fuzz fibres are initiating, expression of *MYB25-like_Dt* was significantly lower in fuzzless NILs than in fuzzy seeded NILs, while higher *MYB25-like_Dt* expression was associated with more seed fuzz across different cotton genotypes. Phenotypic and genotypic analysis of *MYB25-like* homoeoalleles in cottons showing different fibre phenotypes and their crossing progeny indicated that both *MYB25-like_At* and *MYB25-like_Dt* are associated with lint development, and that fuzz development is mainly determined by the expression level of *MYB25-like_Dt* at ~3 d post-anthesis. Expression of Gb fuzzless seeds depends on genetic background and interactions amongst the multiple loci identified. *MYB25-like_Dt* is one of the best candidates for *N*_*2*_.

## Teaser

Five genetic loci were found to be associated with the fuzzless seed trait in *Gossypium barbadense*, one of them containing *MYB25-like_Dt*, the best candidate for the *N*_*2*_ gene.

## Introduction

Mature cotton seeds are covered with two types of fibres: lint (up to ~3.5 cm) and fuzz (<0.5 cm). Both are single-celled, tubular outgrowths that arise from the epidermal cells of the seed coat and are indistinguishable in appearance during the early stages of their growth (Lang, 1938; Stewart, 1975), suggesting that their growth may involve the same physiological and biochemical processes. Lint fibre initials in *Gossypium hirsutum* (Gh) and *G. barbadense* (Gb) start developing on the day of anthesis, i.e. 0 d post-anthesis (dpa), with approximately a quarter to a third of the epidermal cells becoming fibre initials and finally lint fibres (Lang, 1938; Stewart, 1975). Fuzz fibres start developing after the lint at ~4 dpa, do not elongate to the same extent as the lint, and are variable in length and abundance among different genotypes (Lang, 1938; Joshi *et al.*, 1967; Lee *et al.*, 2006), with some cottons having no fuzz fibres, but still producing normal lint fibres.

Fuzzless cotton seeds are referred to as ‘naked seeds’ and have advantages during ginning because they generally require much less force to remove the lint from the seed than fuzzy seeded cottons, and hence less power consumption at the gin and less breakage of the lint fibres (Boykin, 2007; Bechere *et al*., 2009, 2012). A number of fuzzless loci have been reported in cotton, including the dominant *N*_*1*_ and the recessive *n*_*2*_ (Percy & Kohel, 1999). Homozygous *N*_*1*_*N*_*1*_ mutants are completely fuzzless, and lack any tuft (seen in the recessive mutants) at the micropylar tip of the seed and also have a significantly reduced lint percentage (Ware, 1940; Turley & Kloth, 2002; Turley *et al.*, 2007) probably because of delayed lint initiation and their shorter lint (Lee *et al.*, 2006; Turley *et al.*, 2007; Zhang *et al.*, 2007; Romano *et al.*, 2011). In earlier genetic studies, the recessive *n*_*2*_ fuzzless mutant was believed to have a genotype of *n*_*2*_*n*_*2*_. However, Turley & Kloth (2002) demonstrated that a third unlinked recessive locus, *n*_*3*_, is required for expression of the fuzzless trait in accessions carrying *n*_*2*_. This second locus may have confounded some earlier genetic studies of the *n*_*2*_ mutant, which shows variable fuzz development that is influenced by genetic background and environmental conditions (Turley & Kloth, 2002; Rong *et al.*, 2005). Compared with the wild-type TM-1, in which fuzz initiation was observed at 4 dpa, few or no epidermal protrusions were observed in the *N*_*1*_ and *n*_*2*_*n*_*3*_ mutants at the same time point (Zhang *et al.*, 2007). Cotton plants homozygous for all three fuzzless loci (*N*_*1*_*N*_*1*_*n*_*2*_*n*_*2*_*n*_*3*_*n*_*3*_) are fibreless (i.e. lack both lint and fuzz; Turley & Kloth, 2002), so clearly the genes at these loci have central roles in the development of both types of fibres. A new ethyl methanesulfonate-induced mutant, *n*_*4*_^*t*^, was recently reported that appears to be different from the other naked seed loci (Bechere *et al*., 2009, 2012). The *n*_*4*_^*t*^ mutation has been reported to have a less negative effect on lint percentage and hence lint development (Bechere *et al.*, 2012). In addition, no fuzzy seeded but lintless phenotype has ever been observed in cotton, suggesting that fuzz genes are epistatic to lint genes (Du *et al.*, 2001) and/or that development of fuzz and lint are temporally and spatially regulated by the same regulatory genes. The genetic control of fuzz development is clearly very complex and there are many different genes other than these major naked seed genes that modify the amount of fuzz on the seed (Turley & Kloth, 2002) even on the same plant (Kearney & Harrison, 1928), and few of these have been characterized. Cotton breeders have long aimed to develop cotton cultivars with fuzzless seeds and a high lint percentage to capitalize on their ginning advantages, but to achieve that goal, it is essential to identify the genes underlying the regulation of fuzz and lint development and to understand their biological roles and genetic interactions.

Cotton is an allotetraploid with At and Dt subgenomes. The *N*_*1*_ and the *n*_*2*_ genes have been localized to chromosomes 12 (A12) and 26 (D12) (a pair of homoeologous chromosomes), respectively (Endrizzi & Ramsay, 1980; Samora *et al.* 1994). The *n*_*3*_ locus has not yet been mapped, but is reported to be unlinked to *n*_*2*_ (Turley & Kloth, 2002). Commercial Gb cultivars produce some of the highest quality lint fibres of any cotton species, but all appear to contain the *n*_*2*_ gene making them almost universally fuzzless, although there is considerable environmental effect on the amount of fuzz (Kearney & Harrison, 1928). It is not known if all Gb cultivars also carry the *n*_*3*_ locus, but this is highly likely. A recent study using a map-based cloning strategy has identified the dominant *N*_*1*_ gene to be a defective allele of the At-subgenome homoeolog of *MYB25-like* (i.e. *MYB25-like_At*) that generates small interfering RNAs from its 3′ end due to production of an overlapping antisense transcript that post-transcriptionally silences both homoeologs of this gene (Wan *et al.*, 2016). *MYB25-like* is a master regulator involved in fibre initiation and development as silencing *MYB25-like* results in cotton seeds lacking both fuzz and lint fibres (Walford *et al.*, 2011). However, the identities of the *n*_*2*_ and *n*_*3*_ genes are as yet unknown.

High-throughput genotyping and next-generation sequencing technologies are revolutionizing the speed and ability to fine-map and clone genes underlying agronomically important traits, which are usually controlled by multiple genes with complex interactions (Schneeberger, 2014; Zhu *et al.*, 2014*a*). In cotton, a large number of single nucleotide polymorphism (SNP) markers have been reported based on whole genome re-sequencing of diverse cotton cultivars and transcriptome sequencing (Byers *et al.*, 2012; Zhu *et al.*, 2014*b*; Hulse-Kemp *et al.*, 2015*a*; Wang *et al.*, 2015; Fang *et al.*, 2017). A cotton SNP array containing ~63 000 SNPs, mainly from US and Australian cotton cultivars, was recently developed (Hulse-Kemp *et al.*, 2015*b*) and has been widely used by the cotton community for a diversity of studies (Ellis *et al.*, 2016; Li *et al.*, 2016; Wang *et al.*, 2016; Gapare *et al.*, 2017; Hinze *et al.*, 2017; Huang *et al.*, 2017*a*), including identification of the gene responsible for okra leaf shape (Zhu *et al.*, 2016). One of the applications of next-generation sequencing in identification of genes underlying specific phenotypes is mapping-by-sequencing (MBS) where pools of segregating progeny are bulked according to their mutant phenotype and their genome sequenced to identify genomic regions inherited predominantly from the parent displaying the phenotype (Zhu *et al.*, 2017). This approach has been applied in cotton to map genes related to the development and properties of fibres (Thyssen *et al*., 2014, 2015, 2016, 2017; Islam *et al.*, 2016) and branching architectures (Chen *et al.*, 2015). In model plant species, such as Arabidopsis, it is possible to narrow down the resolution of MBS to the single gene level as demonstrated recently by the cloning of an imprinted gene involved in endosperm cellularization (Huang *et al.*, 2017*b*), but this is still difficult in allopolyploid species such as cotton, and therefore final identification of the causative genes usually requires other additional approaches once a genomic region containing the gene is identified.

In this study, we aimed to uncover the genetic basis of the fuzzless trait from *G. barbadense* and to explore the possibility of breeding *G. hirsutum* cottons with fuzzless seeds and without the lint percentage penalty normally associated with this trait that has so far prevented its adoption in commercial breeding programmes. To this end, we developed and used near isogenic lines (NILs) for identification of genetic loci tightly linked to the fuzzless trait using the Cotton SNP63K array and the MBS approach. We found that the fuzzless seed trait in Pima S-7, a Gb cultivar, is controlled by multiple recessive loci, including a locus containing *MYB25-like_Dt*. Our results indicate that lint development is associated with the expression levels of both *MYB25-like_At* and *MYB25-like_Dt* at ~0 dpa, while fuzz development is mainly determined by the expression level of *MYB25-like_Dt* at ~3 dpa.

## Methods and materials

### Plant materials, development of nearly isogenic lines, and segregating populations

One *G. barbadense* (Pima S-7, fuzzless) and four *G. hirsutum* accessions (Sicala 40, Sicala V-2, T586, and Xu142*fl*) were used in genetic and gene expression analyses. Sicala 40 and Sicala V-2 are normal fuzz commercial cultivars producing copious amounts of lint, T586 is a lint bearing cotton genetic standard line containing the dominant fuzzless gene *N*_*1*_ (Endrizzi & Taylor, 1968), while Xu142*fl* is a fibreless mutant known to carry *n*_*2*_ amongst other fibre mutations (Zhang & Pan, 1991; Du *et al.*, 2001). In addition, 134 Gb accessions with variable fuzz phenotypes were selected from the National Mid-term Genetic Bank of Cotton at the Institute of Cotton Research (ICR) of the Chinese Academy of Agricultural Sciences, Anyang, China, and used for association analysis.

An F_2_ population derived from Pima S-7 × Sicala 40 (PS; 169 plants) was used in segregation and fuzz percentage analysis. Pima S-7 and two genetically related Gh cultivars, Sicala V-2 and Sicala 40, were used in the development of a set of NILs each showing fuzzless or fuzzy seeded phenotypes that were selected from a breeder’s population aimed at generating a fuzzless seed in an elite commercial Gh background. After four backcrosses, homozygous fuzzless and normal fuzz NILs were identified and confirmed in BC_4_F_4_ and BC_4_F_5_ generations (see Supplementary Fig. S1 at *JXB* online). Selected BC_4_F_4_ and BC_4_F_5_ plants showing normal fuzz (eight NILs) or reduced fuzz (20 fuzzless or intermediate fuzz NILs) were genotyped using the Cotton SNP63K array. BC_4_F_6_ progeny showing uniform fuzzless or segregating fuzz phenotypes were used in bulk segregant sequencing (Supplementary Fig. S1A). A BC_5_F_2_ population (designated FLNS) derived from a cross between one of the BC_4_F_5_ fuzzless NILs, FLN1-10, and Sicala 40 was used in fuzz segregation and lint percentage analyses. Gene expression analysis was performed using Pima S-7, Sicala 40, T586, Xu142*fl*, and different BC_4_F_5_ NILs that had normal fuzz or were fuzzless. The contributing effects of *MYB25-like* homoeoalleles on fuzz and lint development were evaluated using two more F_2_ populations: PX (92 plants) from Pima S-7 × Xu142*fl* and FLNX (87 plants) from FLN1-10 × Xu142*fl*. The reason for use of Xu142*fl* was that both of its *MYB25-like* homoeologs are very lowly expressed and therefore potentially dysfunctional. All cotton plants, except the 134 Gb accessions, which were planted in the field of ICR’s Experiment Station in Xinjiang, China (2015), were grown in a glasshouse (Canberra, Australia) at 28 ± 2 °C with natural lighting.

### Phenotyping

Fuzzy, intermediate, and fuzzless seed phenotypes were scored based on visual inspection. The fuzz phenotype of the PS F_2_ population and the 134 Gb accessions was measured quantitatively using fuzz percentage, which was calculated using at least 100 seeds and the formula of [(weight of ginned seeds−weight of delinted seeds)/weight of ginned seeds]×100, where the seeds were equilibrated to a constant moisture content before and after sulfuric acid delinting to burn off the fuzz fibres.

### DNA sample preparation and SNP genotyping

DNA extraction, SNP genotyping using the Cotton SNP63K array or KASP (Kompetitive Allele Specific PCR) were performed as previously described (Zhu *et al.*, 2016). For calculation of SNP frequencies based on the SNP array, Pima S-7, Sicala 40 and heterozygous alleles were designated 1, 0 and 0.5, respectively. For *MYB25-like_Dt*, a KASP marker was designed using a SNP located within its coding region (see Supplementary Fig. S2A). The *MYB25-like_At* alleles of Pima S-7 and Xu142*fl* were distinguished using species-specific SNPs (Gb *vs* Gh) and a non-synonymous SNP within the coding region of the Xu142*fl MYB25-like_At* (Supplementary Fig. S2B), respectively. KASP oligos are shown in Supplementary Table S1.

### Bulk segregant analysis and mapping-by-sequencing

DNA was extracted from 24 randomly selected BC_4_F_6_ progeny derived from NILs segregating for the fuzz phenotype and bulked to form the Recessive Fuzzless Bulk1 (RFB1) DNA pool. DNA was extracted from 15 BC_4_F_6_ progeny derived from fuzzless NILs and bulked to form the RFB2 DNA pool. Two barcoded DNA sequencing libraries were created using RFB1 and RFB2, and sequenced in a single lane using the Illumina HiSeq2500 platform at the Australian Genome Research Facility (Melbourne, Australia). Approximately 50 Gb of 100-bp paired-end reads were generated.

After quality check using FastQC, the clean reads of the two bulks were separately mapped to the Sicala 40 genome sequence, which was generated by mapping re-sequenced Sicala 40 reads to the TM-1 (*G. hirsutum*) reference genome (Zhang *et al.*, 2015) using CLC Genomics Workbench (version 7.5.1) with the following parameter settings: mismatch cost, 2; insertion and deletion cost, 3; length fraction, 0.5; similarity fraction, 0.95; and non-specifically matched reads ignored. Variants (including SNPs and indels) between RFB1 or RFB2 and Sicala 40 were identified using the ‘basic variant detection’ module implemented in CLC Genomics Workbench with the following settings: ploidy, 2; default read quality filters (i.e. neighbourhood radius, 5; minimum neighbourhood quality, 15; minimum central quality, 20); minimum read coverage, 10; minimum variant read count, 3; minimum variant frequency, 35%. SNPs were filtered from the results. Average SNP, i.e. Pima S-7 allele, frequency of a 1-Mbp window (500 kb overlapping between the two adjacent windows) was calculated for RFB1 and RFB2 using a sliding-window-based approach and plotted for each chromosome using the physical co-ordinates of the TM-1 genome (Zhang *et al.*, 2015). Candidate regions associated with the recessive fuzzless trait should be homozygous for Pima S-7 in the RFB2 pool and hence have a Pima S-7 allele frequency of 1 or close to 1, while in the segregating RFB1 pool the Pima S-7 allele frequency should be ~0.5 because this pool contains plants that are homozygous Pima S-7, heterozygous Pima S-7 and homozygous Sicala 40.

### Gene expression analysis using quantitative real-time PCR

Two types of tissues, whole ovules and ovule outer integuments (a 3–4-cell layer including the epidermis that produces the lint and fuzz fibres) were used in gene expression analysis. Whole ovules of different developmental stages were collected from normal fuzz (NFN) or fuzzless (FLN) NILs and used for dissection of outer integuments according to a previously reported protocol (Bedon *et al.*, 2014). For the four cotton accessions (Sicala 40, Pima S-7, T586, and Xu142*fl*), whole ovules of different developmental stages were directly used for RNA extraction. Total RNA extraction and quantitative real-time PCR (qRT-PCR) procedures were performed as previously described (Zhu *et al.*, 2009, 2013) except that the reference gene used was the cotton ubiquitin gene (GenBank accession no. EU604080), and the reactions were run on the ViiA7 Real-Time PCR System (Life Technologies) using the FasterStart Universal SYBR Green Master Mix (ROX) (Roche). Gene expression levels were determined based on three biological replicates each with three technical replicates. Primers specifically amplifying *MYB25-like_At* could not be designed, so the expression level of *MYB25-like_At* was thus determined by subtraction of the expression level of *MYB25-like_Dt* from the total expression level of *MYB25-like*. Primers used in qRT-PCR are shown in Supplementary Table S1. All primer pairs had a similar PCR efficiency (89.9–99.2%) determined by LinRegPCR (http://www.hartfaalcentrum.nl/index.php?main=files&sub=LinRegPCR).

## Results

### The fuzzless trait in *G. barbadense* is controlled by multiple recessive genetic loci

**Fig. 1.**
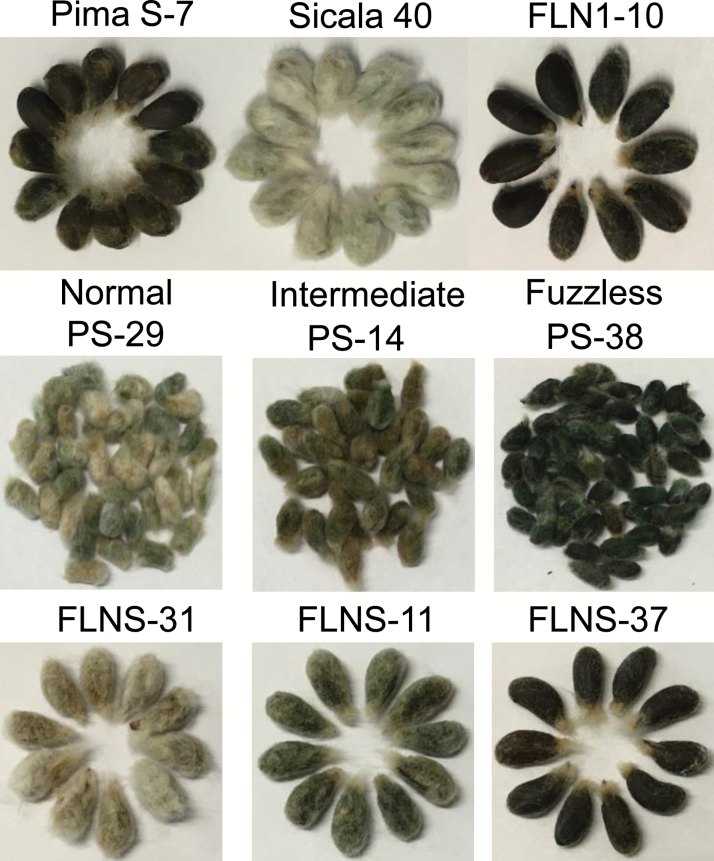
Fuzz phenotypes of the parental lines (Pima S-7, Sicala 40, and the fuzzless NIL, FLN1-10) used in crosses and representative normal fuzz, intermediate, and fuzzless seed phenotypes of the segregating PS and FLNS F_2_ population. PS represents Pima S-7 × Sicala 40. FLN1-10 is a BC_4_F_5_ fuzzless NIL described in Fig. S1A.

F_3_ seeds of 169 F_2_ plants derived from Pima S-7 (Gb) × Sicala 40 (Gh; hereafter named PS) showed a continuous gradation of the fuzz phenotype, from Pima S-7-like (fuzzless) through to Sicala 40-like (normal fuzz) (Fig. 1). Of the 169 F_2_:F_3_ families, seeds of only three families (e.g. PS-38; Fig. 1) looked similar to the naked seeds of Pima S-7, fitting a model with three recessive genes controlling fuzz formation (χ^2^_63:1,1_=0.0497, *P*=0.8236). However, for the FLNS F_2_ population created using Sicala 40 and a fuzzless NIL (FLN1-10; Fig. 1; Supplementary Fig. S1), five out of the 60 F_2_:F_3_ families showed a Pima S-7-like naked seed phenotype (e.g. FLNS-37; Fig. 1), fitting a model with two recessive genes (χ^2^_15:1,1_=0.4444, *P*=0.5050). We noticed that the three fuzzless F_2_:F_3_ families observed in the PS population were not completely fuzzless and had a little more fuzz than Pima S-7, and we also observed transgressive segregants (with more fuzz than Sicala 40) in the PS but not in the FLNS F_2_ populations. These results indicate that (i) fuzz development is complex with at least three recessive genes being involved in the regulation of the fuzzless trait in Pima S-7, and (ii) Pima S-7 must contain additional epistatic modifier(s) affecting the function and/or the interaction of the three loci responsible for its fuzzless phenotype that have not been identified here due to the small population sizes used.

### Identification of genetic loci, including the *MYB25-like_Dt* containing locus, associated with low seed fuzz

**Fig. 2.**
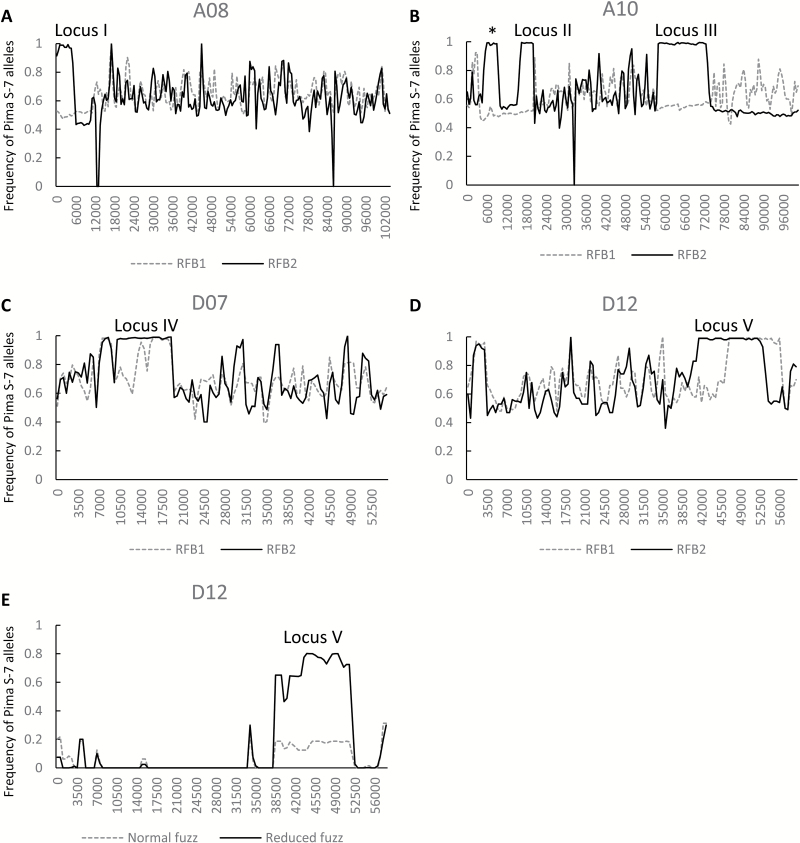
Identification of genetic loci associated with fuzz development. (A–D) Frequency distribution of the Pima S-7 alleles on chromosomes A08, A10, D07, and D12. The results were based on sequencing of bulked segregants showing fuzzless (RFB2) or segregating fuzz phenotypes (RFB1). The graphs were generated using a sliding-window-based approach (1 Mbp window length and 500 kb overlap between the two adjacent windows) with the *x*-axis representing chromosomal coordinates (kb). The regions indicated by I, II, III, IV and V were the five loci associated with fuzz development, whereas the region indicated by an asterisk was not associated with fuzz development based on fuzz percentage analysis of the PS F_2_ population and the results from other segregating NILs genotyped by the Cotton SNP63K array. (E) The Pima S-7 allele frequency of the SNP markers polymorphic between Pima S-7 and Sicala 40 on D12 (267 markers). The result was based on the genotyping of eight normal fuzz and 20 reduced fuzz (including fuzzless) BC_4_F_4_ or BC_4_F_5_ NILs using the Cotton SNP63K array. The graph was generated using the same approach as those in (A–D).

We used NILs with different fuzz phenotypes to identify genetic loci controlling fuzz development (see Supplementary Fig. S1). Eight normal fuzz and 20 reduced fuzz (fuzzless or intermediate) BC_4_F_4_ or BC_4_F_5_ NILs were used in the SNP array analysis. Of the ~63 000 SNPs, 5426 were polymorphic between Pima S-7 and Sicala 40, and these were distributed across all 26 chromosomes, although chromosomes A02, A04, and D04 had less than 20 SNPs each (Supplementary Table S2). The Pima S-7 allele frequency of the polymorphic SNPs was plotted for each chromosome based on their genomic coordinates relative to the TM-1 genome. For the pool of the 20 reduced fuzz NILs, each candidate locus associated with low fuzz would have a Pima S-7 allele frequency close to 1 because the fuzzless NILs should be homozygous for the Pima S-7 allele and the NILs close to being fuzzless could be still heterozygous for the Pima S-7 allele. For the pool of the eight normal fuzz NILs, each candidate locus would have a Pima S-7 allele frequency <0.5 because these NILs could contain heterozygous or null Pima S-7 alleles. Three regions on chromosomes A12, D05, and D12 met these criteria (Supplementary Table S3; Fig. 2E; Supplementary Fig. S3). The A12 locus contained only three SNP markers and all of them were mapped to the D12 interval based on previous interspecific mapping results (Hulse-Kemp *et al.*, 2015*b*) and so can be excluded as their positions were incorrectly assigned based on blasting. We further created and sequenced two bulked DNA libraries, RFB1 (progeny of normal fuzz NILs) and RFB2 (progeny of fuzzless NILs). Genome-wide SNP identification was performed for RFB1 and RFB2 using Sicala 40 as a reference. Candidate loci associated with low fuzz seeds would be expected to have a Pima S-7 allele frequency of ~0.5 and 1 in RFB1 and RFB2, respectively. Nine regions on seven different chromosomes met these criteria (Supplementary Table S3; Fig. 2A–D; Supplementary Fig. S4). The D12 locus was identified in both MBS and SNP array analyses, and so represents the best candidate for a major locus contributing to fuzzless seeds.

**Fig. 3.**
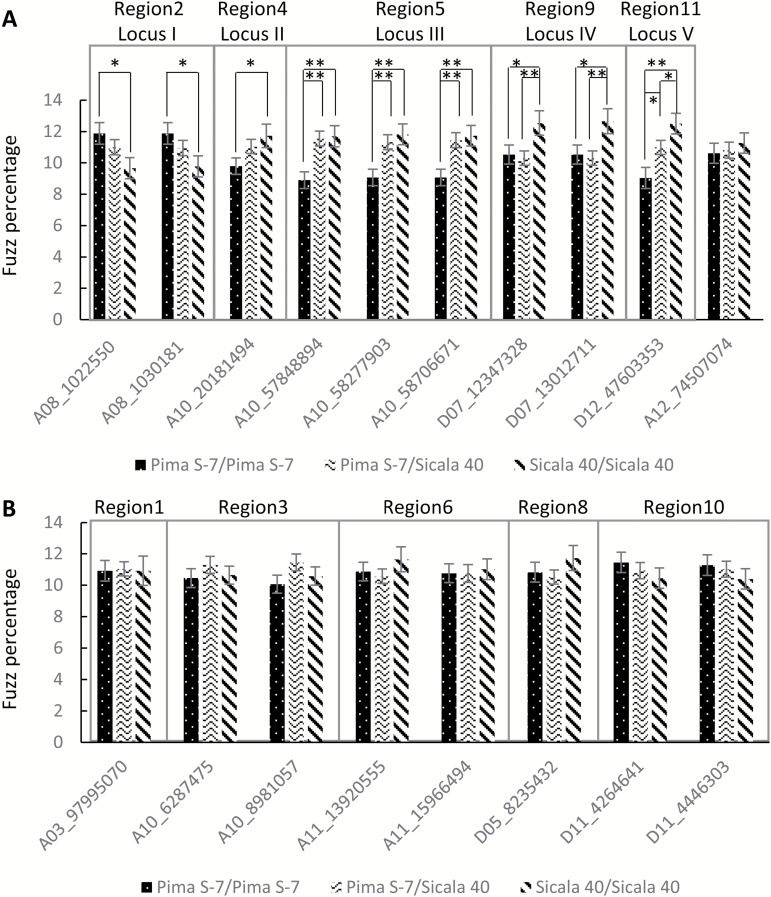
Association analysis between SNP markers and fuzz percentages of the PS F_2_:F_3_ seeds. F_2_ plants were genotyped using the markers shown below the *x*-axis. For each marker, F_2_ plants were grouped based on their genotypes, i.e. homozygous Pima S-7 (Pima S-7/Pima S-7) or Sicala 40 (Sicala 40/Sicala 40), or heterozygous (Pima S-7/Sicala 40) individuals. Error bars represent standard error. (A) Regions or genetic loci associated with fuzz percentage. Asterisks denote significant difference determined by Student’s *t*-test: *P<0.05, **P<0.01. A12_74507074 was a randomly selected SNP marker and used as a negative control. (B) Regions not associated with fuzz development.

Because the fuzzless NILs used were self-pollinated for a few generations, some of the ten candidate regions identified could thus be false positives due to fixation of Pima S-7 alleles not related to the fuzzless phenotype. To refine these candidate loci we designed KASP marker assays (between 1 and 3 assays for each region) across all the ten candidates and genotyped the PS F_2_ population with those markers and also measured fuzz percentage for each F_2_ individual. For each SNP marker, F_2_ plants were grouped based on their genotypes (i.e. homozygous Pima S-7 or Sicala 40, and heterozygous) and the average fuzz percentage was compared for each genotype. Of the ten candidate regions, five (regions 2, 4, 5, 9, and 11) were found to be positively or negatively correlated with the amount of seed fuzz. They were designated loci I–V (Supplementary Table S3; Fig. 3). For loci II–V, plants with a genotype of homozygous Pima S-7 regions had a significantly lower fuzz percentage than those with a genotype of homozygous Sicala 40, but for locus I (region 2), plants with a genotype of homozygous Pima S-7 had a significantly higher fuzz percentage than those with a genotype of homozygous Sicala 40 (Fig. 3A), suggesting Pima S-7 contained an enhancer rather than an inhibitor of fuzz development within that locus. For locus V (region 11), the fuzz percentage of heterozygous plants was intermediate and significantly different from either homozygous Pima S-7 or Sicala 40 (Fig. 3A), suggesting a major and additive effect of locus V on fuzz percentage, whereas the other loci were closer to one or other of the homozygous parental genotypes. This was supported by the fuzz phenotypes of the segregants within the FLNS population, as its F_2_:F_3_ families with a homozygous Pima S-7 region at locus V always had less fuzz than those with a homozygous Sicala 40 region (see Supplementary Fig. S5), and this trend was not observed for the other loci.

By checking the co-segregation of markers on the individual plants used with the Cotton SNP63K array analysis and the overlap between the SNP array and MBS analyses, we were able to deduce the intervals containing the candidate gene(s) for the five individual loci that contained between 20 and >300 annotated genes. For example, the size of the overlapping interval of locus V is ~1.4 Mbp containing 84 annotated genes (see Supplementary Tables S3 and S4). Fine mapping and/or other strategies will be required to pinpoint the actual causal gene(s) within these loci. However, one of the locus V genes is *MYB25-like_Dt* (*Gh_D12G1628*), a homoeolog of *MYB25-like_At* that has been shown to be critical for fuzz development (Wan *et al.*, 2016). *MYB25-like_Dt* is thus the best candidate at this locus.

We also found that the average fuzz percentage of the 134 Gb accessions used in this study was low at 2.68% (with a range of 0.29–9.20%), and generally considerably less than the 10–12% found with normal fuzz Gh cultivars. Although the majority of these Gb accessions were fuzzless or close to fuzzless, a few were normal fuzzy seeded (e.g. M210353 in Supplementary Fig. S6). Despite their variable fuzz phenotypes, all of these Gb accessions had a homozygous Pima S-7 genotype in the five loci we have identified, suggesting that there could be additional modifiers of those five fuzzless loci in the rare fuzzy seeded Gb accessions, but this will have to await a more detailed genetic and molecular analysis to ensure that these accessions have not been misclassified or are partial hybrids with Gh.

### Expression of *MYB25-like_Dt* is down-regulated in outer integuments of fuzzless NILs during fuzz initiation

**Fig. 4.**
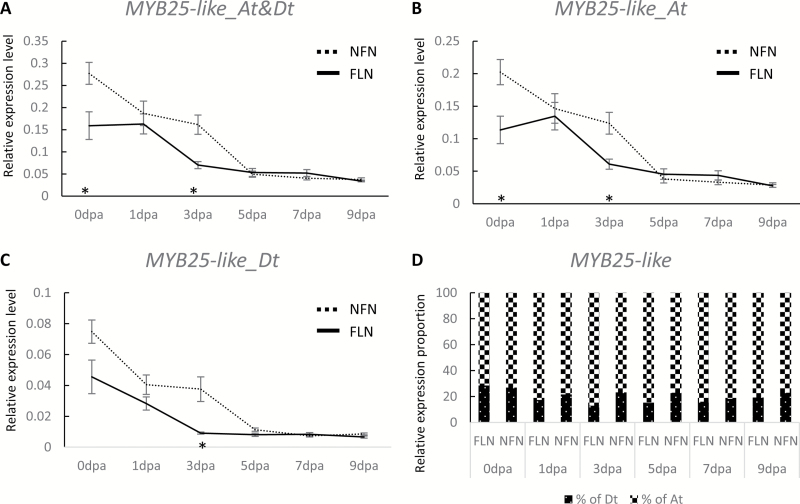
Expression profile of *MYB25-like* in 0–9 dpa outer integuments of the normal fuzz NIL (NFN, homozygous Sicala 40 allele for *MYB25-like_Dt*) and fuzzless NIL (FLN, homozygous Pima S-7 allele for *MYB25-like_Dt*). (A–C) Expression level changes of *MYB25-like*, *MYB25-like_At* and *MYB25-like_Dt*. *Significant difference at *P*<0.05 determined by one-way ANOVA. (D) Relative expression proportion of the At and Dt homoeologs of *MYB25-like* in 0–9 dpa outer integuments of NFN and FLN.

In 0–9 dpa ovule outer integuments of NILs with normal fuzz (NFN) or fuzzless (FLN) seeds, *MYB25-like_At* and *MYB25-like_Dt* had a similar expression profile to each other, but were differentially expressed in both seed phenotypes, with approximately 70–80% of the total expression of *MYB25-like* contributed by its At homoeolog (Fig. 4). In NFN, the expression level of *MYB25-like_Dt* dropped steadily from 0 to 5 dpa, and then remained relatively low thereafter. The fuzzless FLN had a lower expression level of *MYB25-like_Dt* from 0 dpa and declined more rapidly than in the normal fuzz NFN NILs (Fig. 4C), such that there was little expression at 3 dpa, just prior to when fuzz fibres would normally initiate. Although the significantly reduced fuzz percentage observed in F_2_ plants homozygous for the Pima S-7 allele of *MYB25-like_Dt* (i.e. *MYB25-like_Dt*^*PimaS7/PimaS7*^; Fig. 3A) could be contributed by other gene(s) within the same linkage block as *MYB25-like_Dt*, this expression profile of *MYB25-like_Dt*, together with the previously reported role of its At homoeolog in fuzz development (Wan *et al.*, 2016), provides additional support for *MYB25-like_Dt* being one of the best candidates for a gene involved in the reduced fuzz of Pima S-7.

The protein coding sequences of *MYB25-like_Dt* between the reference sequence of TM-1 (fuzzy seeded) and Pima S-7 differ at three nucleotides with two of them giving rise to changed amino acids in Pima S-7 (see Supplementary Fig. S2A). In both cases, however, the changed bases are all found in either the Dt-subgenome or the At-subgenome homoeologs from other cultivars including TM-1, Sicala 40, and Xu142 (Supplementary Figs S2A and S7) that have fuzzy seeds, so these SNPs are unlikely to lead to a loss of function of *MYB25-like_Dt* in Pima S-7. The core promoters (−1 to −250 bp) of *MYB25-like_Dt* and *MYB25-like_At* in Pima S-7, Sicala 40, and TM-1 were very similar, but the remaining upstream sequences of their promoters were quite different. Whether any of these differences is the cause of their differential expression remains to be investigated by fusing different lengths of promoter to reporter genes. The promoter of *MYB25-like_Dt* in Pima S-7, compared with that in Sicala 40 and TM-1, had seven differences, including three SNPs, two small indels (1 bp deletion or insertion), and two deletions (>20 bp) (Supplementary Fig. S8).

### Expression levels of *MYB25-like_Dt* but not of *MYB25-like_At* predominantly determine the fuzz phenotypes

**Fig. 5.**
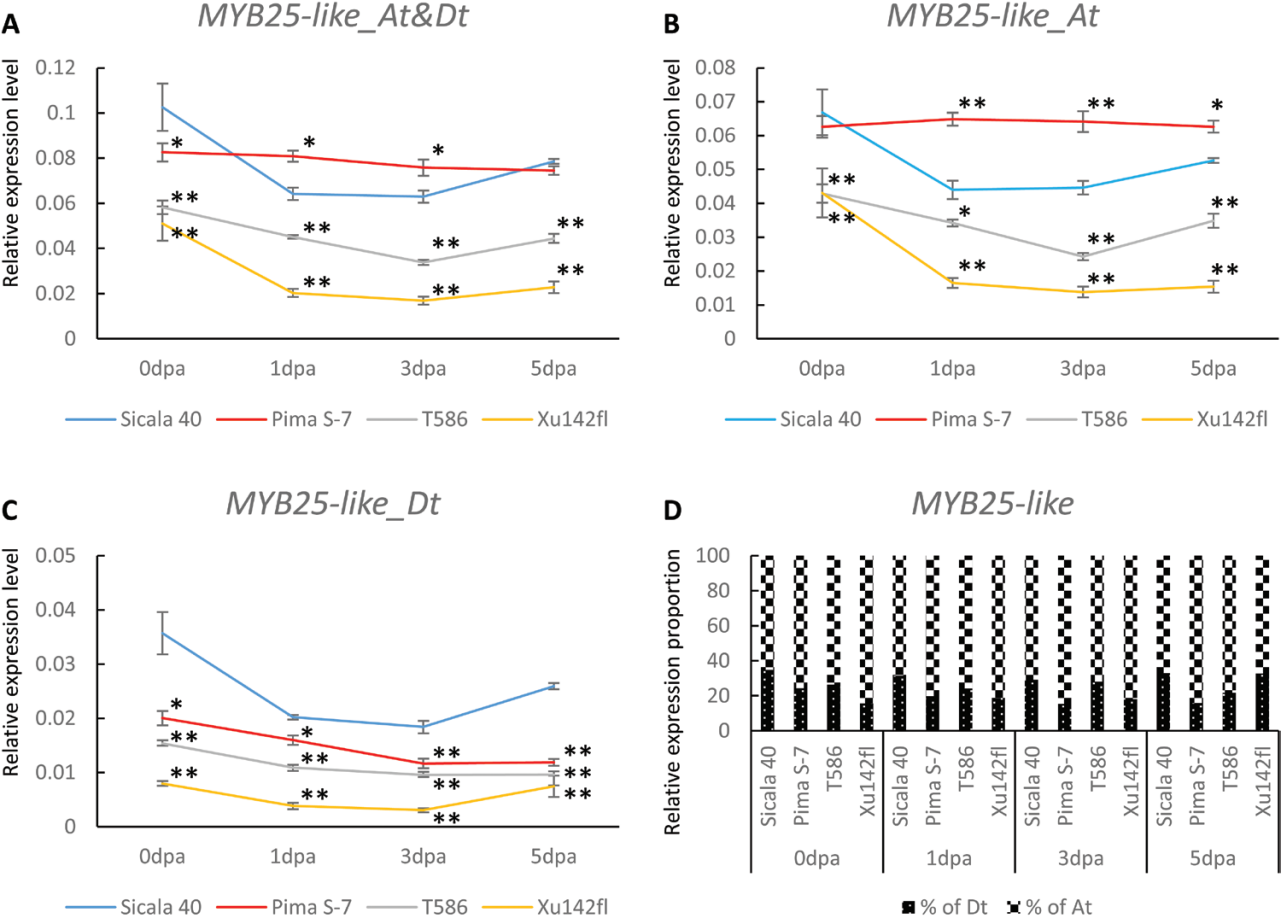
Expression profile of *MYB25-like* in 0–5 dpa whole ovules of four cotton accessions with different fuzz and lint phenotypes. (A) Total expression level of *MYB25-like* (both homoeologs). (B) Expression level of *MYB25-like_At*. (C) Expression level of *MYB25-like_Dt*. Asterisks denote a significant difference compared with the expression level in Sicala 40 determined by one-way ANOVA: *P<0.05, **P<0.01. (D) Relative expression proportion of the At and Dt homoeologs of *MYB25-like* in 0–5 dpa whole ovules of the same four cotton accessions.

We compared the expression levels of the two *MYB25-like* homoeologs in 0–5 dpa whole ovules of the linted-fuzzless T586 (*N*_*1*_) and fibreless Xu142*fl* lines with those of Pima S-7 and Sicala 40 (Fig. 5). At 0 dpa when lint fibres have started to initiate, the total expression level of *MYB25-like* was highest in Sicala 40 and lowest in Xu142*fl* with a ranking of Sicala 40>Pima S-7>T586>Xu142*fl* (Fig. 5A), which is consistent with their rankings in final lint percentage (Sicala 40: 41.58%; Pima S-7: 34.51%; T586: 11.37%; Xu142*fl*: 0), suggesting that the expression level of *MYB25-like* at ~0 dpa is correlated with lint fibre initiation and development. In 1–5 dpa ovules, Pima S-7 had a more lowly expressed *MYB25-like_Dt* than Sicala 40, but a significantly more highly expressed *MYB25-like_At* (Fig. 5B, C) despite being fuzzless, supporting the association between the expression of *MYB25-like_Dt*, but not *MYB25-like_At*, and the poor fuzz development in Pima S-7. From 0 to 5 dpa, the expression levels of both *MYB25-like* homoeologs were consistently lower in Xu142*fl* and T586 than in Sicala 40 and Pima S-7. Around the time of fuzz initiation at 3 dpa, Xu142*fl* had the lowest expression of both homoeologs in any of the genotypes, but particularly the Dt homoeolog. In addition, there is a non-conservative substitution at position 314 within the conserved MYB DNA binding domain of *MYB25-like_At* in Xu142*fl* (see Supplementary Fig. S2B) that is likely to negatively impact on the activity of this transcription factor, supporting the assertion previously reported (Walford *et al.*, 2011) that this fibreless mutant line has two dysfunctional *MYB25-like* homoeologs.

To investigate the effect of different *MYB25-like* homoeoalleles from Gh and Gb on lint and fuzz development, we generated two other segregating F_2_ populations, PX from Pima S-7 x Xu142*fl* and FLNX from FLN1-10 (*MYB25-like_Dt*^*Pima*^^*S7/Pima*^^*S7*^ locus in an essentially Sicala 40 genetic background) × Xu142*fl* and examined lint and fuzz phenotypes of the F_2_ plants with different allele combinations determined by the presence of SNPs specific to each of the *MYB25-like* homoeologs and their parental origins (Table 1; Supplementary Fig. S9). The difference in these two populations was in the maternal allele of *MYB25-like_At*, which was from Pima S-7 in PX and from Sicala 40 in FLNX, both of which should be highly expressed and as demonstrated below, fully functional (Fig. 5B). For each F_2_ population, individual plants were classified into 9 types (1–9 and 10–18 for PX and FLNX, respectively) based on the homozygosity or heterozygosity of parental origin for the At and Dt homoeolog of *MYB25-like*. A number of observations were clear from this analysis:

**Table 1.** Segregation of the fuzzless trait by *MYB25-like* homoeoallele combination in two F_2_ populations

Type	*MYB25-like_At* ^*a*^	*MYB25-like_Dt* ^*a*^	No. of plants (%)					Lint^*d*^	Significance^*e*^	
			Normal	Intermediate	Fuzzless	Tufted	Not tufted	(%)	*P*<0.05	*P*<0.01
**The PX F**_**2**_ **population (Pima S-7 × Xu142*fl***)										
1	Xu142*fl*(M)/Xu142*fl*(M)	Xu142*fl*(M)/Xu142*fl*(M)	0 (0)	0 (0)	5 (5.43)^*b*^	0 (0)	5 (5.43)	0.77	a	A
2	Xu142*fl*(M)/Xu142*fl*(M)	Xu142*fl*(M)/Pima S-7(M)	0 (0)	0 (0)	11 (11.96)^*c*^	0 (0)	11 (11.96)	17.17	b	B
3	Xu142*fl*(M)/Xu142*fl*(M)	Pima S-7(M)/Pima S-7(M)	0 (0)	0 (0)	3 (3.26)	0 (0)	3 (3.26)	22.44	bc	BC
4	Xu142*fl*(M)/Pima S-7(N)	Xu142*fl*(M)/Xu142*fl*(M)	0 (0)	0 (0)	15 (16.30)	10 (10.87)	5 (5.43)	24.21	bc	CD
5	Xu142*fl*(M)/Pima S-7(N)	Xu142*fl*(M)/Pima S-7(M)	0 (0)	1 (1.09)	24 (26.09)	23 (25.00)	2 (2.17)	27.26	d	CD
6	Xu142*fl*(M)/Pima S-7(N)	Pima S-7(M)/Pima S-7(M)	1 (1.09)	0 (0)	6 (6.52)	5 (5.43)	2 (2.17)	28.94	de	D
7	Pima S-7(N)/Pima S-7(N)	Xu142*fl*(M)/Xu142*fl*(M)	1 (1.09)	1 (1.09)	5 (5.43)	7 (7.61)	0 (0)	29.61	de	D
8	Pima S-7(N)/Pima S-7(N)	Xu142*fl*(M)/Pima S-7(M)	3 (3.26)	7 (7.61)	2 (2.17)	11 (11.96)	1 (1.09)	29.99	de	D
9	Pima S-7(N)/Pima S-7(N)	Pima S-7(M)/Pima S-7(M)	3 (3.26)	2 (2.17)	2 (2.17)	7 (7.61)	0 (0)	31.03	e	D
**The FLNX F**_**2**_ **population (FLN1-10 × Xu142*fl***)										
10	Xu142*fl*(M)/Xu142*fl*(M)	Xu142*fl*(M)/Xu142*fl*(M)	0 (0)	0 (0)	5 (5.49)	0 (0)	5 (5.49)	0	a	A
11	Xu142*fl*(M)/Xu142*fl*(M)	Xu142*fl*(M)/Pima S-7(M)	0 (0)	0 (0)	14 (15.38)	1 (1.10)	13 (14.29)	31.12	b	B
12	Xu142*fl*(M)/Xu142*fl*(M)	Pima S-7(M)/Pima S-7(M)	0 (0)	0 (0)	4 (4.40)	2 (2.20)	2 (2.20)	36.50	cd	CD
13	Xu142*fl*(M)/Sicala 40(N)	Xu142*fl*(M)/Xu142*fl*(M)	0 (0)	0 (0)	11 (12.09)	0 (0)	11 (12.09)	31.26	b	B
14	Xu142*fl*(M)/Sicala 40(N)	Xu142*fl*(M)/Pima S-7(M)	0 (0)	0 (0)	25 (27.47)	13 (14.29)	12 (13.19)	36.72	cd	CD
15	Xu142*fl*(M)/Sicala 40(N)	Pima S-7(M)/Pima S-7(M)	0 (0)	1 (1.10)	4 (4.40)	5 (5.49)	0 (0)	39.30	d	D
16	Sicala 40(N)/Sicala 40(N)	Xu142*fl*(M)/Xu142*fl*(M)	0 (0)	0 (0)	8 (8.79)	5 (5.49)	3 (3.30)	34.74	c	BC
17	Sicala 40(N)/Sicala 40(N)	Xu142*fl*(M)/Pima S-7(M)	0 (0)	1 (1.10)	6 (6.59)	7 (7.69)	0 (0)	36.58	cd	CD
18	Sicala 40(N)/Sicala 40(N)	Pima S-7(M)/Pima S-7(M)	0 (0)	7 (7.69)	5 (5.49)	11 (12.09)	1 (1.10)	37.22	cd	CD

^*a*^ M and N represent mutated and normal alleles, respectively; see Supplementary Table S1 for markers used in distinguishing different alleles.

^*b*^ Four were completely lintless and one was close to lintless.

^*c*^ Four were close to lintless.

^*d*^ Weight of lint as a percentage of total seed cotton weight.

^*e*^ Values with the same letter were not significantly different determined by Student’s *t*-test.

(i) Fuzzless-lintless seeds (all lacking a micropylar tuft) (types 1 and 10, Table 1) were only produced when the two homozygous defective homoeologs, *MYB25-like_At*^*Xu142fl/Xu142fl*^ and *MYB25-like_Dt*^*Xu142fl/Xu142fl*^, were combined together, as in the original Xu142*fl* fibreless parent. Replacing a single copy of *MYB25-like_Dt*^*Xu142fl*^ with the Pima S-7 Dt allele (types 2 and 11) or the more highly expressed At allele from Pima S-7 (type 4) was sufficient to allow lint, but not fuzz, to develop on the seeds. This suggests that *MYB25-like_Dt*^*PimaS7*^ and *MYB25-like_At*^*PimaS7*^ both produce fully functional MYB proteins able to activate lint fibre initiation. However, although *MYB25-like_At*^*PimaS7*^ is highly expressed from 0 through to 5 dpa (Fig. 5B), a single copy (type 4) was unable to rescue fuzz fibre production (Table 1), although two copies (type 7) allowed the development of some intermediate and normal fuzzy seeds in some plants, suggesting that fuzz and lint development are both dependent on gene dosage and hence total expression of *MYB25-like* homoeoalleles at specific times during development. Interestingly, plants in the PX population with homozygous Pima S-7 alleles for both homoeologs (type 9) were not all fuzzless like the Pima S-7 parent, attesting to the influence of some of the other identified fuzzless loci. All plants in the two populations have a lowly expressed *MYB25-like_Dt* allele, i.e. *MYB25-like_Dt*^*Xu142fl*^ or *MYB25-like_Dt*^*PimaS7*^, and regardless of whether their *MYB25-like_At* allele was functional or mutant, produced mostly fuzzless or reduced fuzz seeds, suggesting it is the expression level of the Dt homoeolog that is critical for fuzz development, but either homoeolog can support lint fibre development provided that it expresses a functional protein during lint initiation. As Pima S-7 and Xu142*fl* are both believed to carry alleles of *n*_*2*_ causing their fuzzless seeds, this provides support for *MYB25-like_Dt* being a good candidate for *N*_*2*_.
(ii) The Gb allele *MYB25-like_At*^*PimaS7*^ was less effective than that from Gh, *MYB25-like_At*^*Sicala40*^, in suppressing fuzz development in the presence of mutated Dt homoeologs, producing fewer fuzzless and more intermediate and fuzzy seeded F_2_ segregants (comparing types 7–9 with types 16–18), perhaps because of its higher expression during fuzz initiation (Fig. 5B), or because of other modifier genes brought in with the different genetic backgrounds.
(iii) Lint percentage (and hence lint fibre development) was largely determined by the functionality and expression (gene dosage) of the At homoeolog of *MYB25-like*, but could also be influenced by the Dt homoeolog. The negative effects of the defective *MYB25-like_Dt* on lint percentage was less severe in the predominantly Gh background of the FLNX plants than in the Gb background of the PX plants that had lower lint percentages with all allele combinations other than in those with fibreless seeds (Table 1).

### Breeding fuzzless cottons without a penalty on lint fibre production is possible

Fuzzless seeds have historically been associated with an undesirable lint percentage (Ware, *et al.* 1944), most likely due to both fuzz and lint fibres being regulated by differential expression of the same genes. The *N*_*1*_ gene, i.e. the dysfunctional *MYB25-like_At*, has a strong negative effect on lint development (Turley *et al.*, 2007), and therefore our ability to breed cottons with both *N*_*1*_ and a high lint percentage is quite poor. Most commercial Gb cultivars have a relatively high lint percentage (~34%), so their fuzzless seed trait should have smaller deleterious effects on lint development. To know whether it is possible to combine the fuzzless seed trait of Gb with the high lint percentage of Gh, we created a BC_5_F_2_ population (FLNS from FLN1-10 × Sicala 40) and measured lint percentages of individual plants. These plants segregated for all five fuzzless associated loci, and contained homozygous *MYB25-like_At* alleles from Sicala 40, i.e. *MYB25-like_At*^*Sicala40/Sicala40*^, which is expressed at a similar level to *MYB25-like_At*^*PimaS7/PimaS7*^ at 0 dpa. Overall, the average lint percentage of the BC_5_F_2_ plants with a genotype of *MYB25-like_Dt*^*Sicala40/Sicala40*^ (normal fuzz) and *MYB25-like_Dt*^*PimaS7/PimaS7*^ (fuzzless) were similar (41.96% and 41.71%, respectively), and as high as that of the elite Gh cultivar Sicala 40 (41.58%). These results suggest that Sicala 40 contains alleles that are able to compensate for any negative effects of *MYB25-like_Dt*^*PimaS7*^ on lint percentage. Individually, the Pima S-7 allele of loci V and II had a positive and negative effect on lint percentage, respectively, whereas the other three loci had no significant effect on lint percentage (see Supplementary Fig. S10).

## Discussion

Identification and characterization of genes contributing to initiation of lint and fuzz fibres is essential for understanding the biological processes underlying fibre development and for improvement of lint yield through traditional breeding or transgenic approaches. Several fibreless or fuzzless mutants, such as Xu142*fl*, SL1-7-1, *N*_*1*_, and *n*_*2*_, have been reported and characterized (Zhang & Pan, 1991; Percy & Kohel, 1999; Turley & Kloth, 2008). They are valuable genetic resources for uncovering genes related to lint and fuzz fibre development. Transcriptome analyses using some of these fibre mutants have identified a large number of genes with a potential role in fibre development (Wu *et al.*, 2006; Padmalatha *et al.*, 2012; Wan *et al.*, 2014); however, only a few of those genes (*GhMYB25-like*, *GhVIN1*, and *GhJAZ2*) have been shown to be essential for fibre initiation and development (Walford *et al.*, 2011; Wang *et al.*, 2014; Hu *et al.*, 2016). Using a forward genetics approach, Wan *et al.* (2016) identified the dominant fuzzless *N*_*1*_ gene to be a dysfunctional allele of *MYB25-like_At* that generates siRNAs and showed that suppression of *MYB25-like_At* by virus-induced gene silencing phenocopied the fuzzless phenotype. In this study, we identified five loci associated with the fuzzless seed trait from Pima S-7 with the *MYB25-like_Dt*-containing locus having a more prominent effect on fuzz development than the other loci (Fig. 3A; Supplementary Fig. S5).

We propose that *MYB25-like_Dt* is a candidate for the *N*_*2*_ gene based on a number of pieces of evidence. First, *N*_*1*_ and *n*_*2*_ have previously been assigned to the two homoeologous chromosomes A12 and D12, respectively (Endrizzi & Ramsay, 1980). *N*_*1*_ has been shown to be localized to an interval containing *MYB25-like_At* (Wan *et al.*, 2016), and here we show that there is a major locus associated with the fuzzless seed phenotype of Gb carrying *n*_*2*_ that maps to an interval containing its homoeolog, *MYB25-like_Dt.* While there are several other loci that associated with fuzzless seeds in different genetic populations segregating for *n*_*2*_, only the locus containing *MYB25-like_Dt* is on D12, known to contain *n*_*2*_. Second, in a highly introgressed population (FLNS) that is predominantly in a Sicala 40 background, those lines carrying the allele of *MYB25-like_Dt* from Pima S-7 all have much reduced fuzz relative to lines carrying the corresponding wild-type allele from Sicala 40, regardless of the combinations of loci for the other four fuzzless associated regions from Gb (see Supplementary Fig. S5). Third, expression of *MYB25-like_Dt* when fuzz fibres begin to initiate at 3 dpa is consistently much lower in NILs and other lines producing fuzzless seeds compared with those with fuzzy seeds, whereas the expression of *MYB25-like_At* in Pima S-7 is even higher than in the fuzzy seeded cultivar Sicala 40 and so is not indispensable for fuzz development. *MYB25-like_Dt* from Pima S-7 does not appear to contain any obvious deleterious mutations within its coding region, and a single copy of this gene is able to restore lint development in lines containing otherwise defective homoeoalleles of *MYB25-like* from Xu142*fl*. The Pima S-7 *MYB25-like_Dt* locus must thus be sufficiently expressed at 0 dpa and produce a functional protein to be able to activate transcription of its downstream gene(s), at least within the lint fibre pathway. There are a number of upstream sequence differences between the *MYB25-like_Dt* alleles from Pima S-7 and Sicala 40 (Supplementary Fig. S8) that may be responsible for the low expression of the Pima S-7 allele during fuzz initiation in lines carrying *n*_*2*_, but further experiments will be required to verify that they are the causal mutations that convert *N*_*2*_ into *n*_*2*_.

Taking advantage of those dysfunctional *MYB25-like* homoeoalleles in Xu142*fl*, we used it to infer both the functionality and the dosage effects of *MYB25-like_Dt*^*PimaS7*^ in fuzz development by comparing fuzz phenotype of F_2_ segregants with *MYB25-like_Dt*^*Xu142fl/Xu142fl*^ or *MYB25-like_Dt*^*PimaS7/PimaS7*^ in common *MYB25-like_At* backgrounds (Table 1). This investigation allowed us to not only demonstrate a key role for *MYB25-like_Dt* in both fuzz and lint development, but also to uncover subtle differences in expression and dosage effects between *MYB25-like_Dt*^*PimaS7*^ and *MYB25-like_Dt*^*Xu142fl*^ alleles in fuzz and lint fibre development. In addition, the loss of fuzz production by *MYB25-like_Dt*^*PimaS7*^ could be alleviated by the relatively highly expressed *MYB25-like_At*^*PimaS7*^ allele that was more effective than the less expressed *MYB25-like_At*^*Sicala40*^ allele (Table 1; Fig. 5B).

**Fig. 6.**
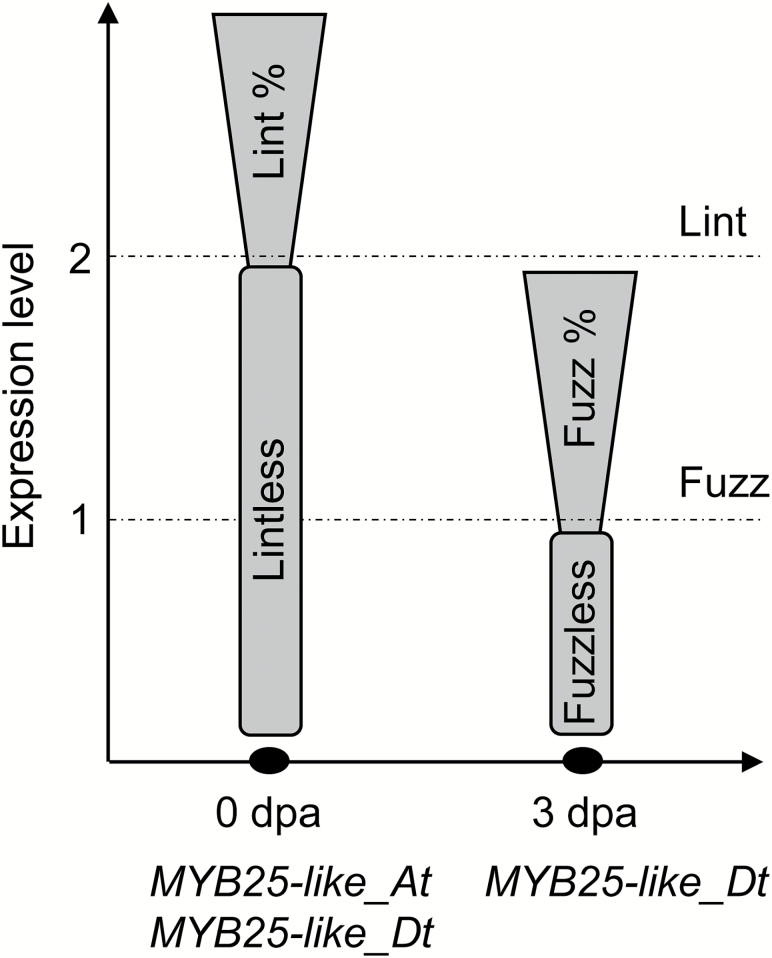
Working model for the role of *MYB25-like* homoeologs in lint and fuzz development. Initiation and development of lint are determined by the total expression level of both homoeologs of *MYB25-like* at ~0 dpa, while initiation and development of fuzz are mainly governed by the expression level of *MYB25-like_Dt* at ~3 dpa, although *MYB25-like_At* may have the ability to compensate for lack of *MYB25-like_Dt* at ~3 dpa. If the expression level of *MYB25-like* is below the threshold level 2 at ~0 dpa, no lint fibre will initiate; otherwise lint fibres develop and their amount is positively correlated with the total expression level of *MYB25-like*. If the expression level of *MYB25-like_Dt* is lower than the threshold level 1 at ~3 dpa, cotton seeds will be fuzzless; otherwise fuzz fibres develop and their amount is positively correlated with the expression level of *MYB25-like_Dt*. When the total expression levels of *MYB25-like* at ~0 dpa and of *MYB25-like_Dt* at ~3 dpa are lower than the threshold level 2 and 1, respectively, cotton seeds will be fibreless (e.g. Xu142*fl*). When the total expression level of *MYB25-like* at ~0 dpa is higher than the threshold level 2 but the expression level of *MYB25-like_Dt* at ~3 dpa is below the threshold level 1, cotton seeds will be fuzzless (e.g. T586). No mutant with lintless and fuzzy seed has ever been reported; that is probably because cottons with an expression level of *MYB25-like_Dt* at ~3 dpa high enough for fuzz development usually have a high enough total expression level of *MYB25-like_Dt* and *MYB25-like_At* at ~0 dpa for lint development.

Based on our results, we proposed a working model for the role of *MYB25-like* homoeologs in lint and fuzz development (Fig. 6). According to this model, transferring a homozygous allele with low expression at ~3 dpa, such as *MYB25-like_Dt*^*PimaS7/PimaS7*^, into a Gh background by backcrossing such that there is a high level of total *MYB25-like* at ~0 dpa to enhance lint fibre development should allow the breeding of elite commercial cotton lines with fuzzless seeds and without any penalty on lint yield, provided that other yield components (such as seeds per boll and bolls per plant) are not affected. We believe we are well on the way to achieving that result.

Interactions between *MYB25-like_At*, *MYB25-like_Dt*, and other loci indicate that the genetic model regulating the fuzzless seed trait from Pima S-7 is genetic background dependent. For instance, the female parents of the PS and FLNS F_2_ populations (Pima S-7 and FLN1-10, respectively) had the same genotype at loci I–V (i.e. homozygous Pima S-7), but their F_2_ populations showed a three- and two-gene-model for the fuzzless trait, respectively. In addition, the PS but not the FLNS F_2_ population showed transgressive segregation, and a few Gb accessions (with homozygous Pima S-7 alleles at all five loci) produce considerable amounts of fuzz (see Supplementary Fig. S6). These results suggest the presence of as yet unknown modifier(s) affecting expression of the fuzzless genes or the interaction amongst the fuzzless genes in the Gb background, consistent with the previous finding that the genetics of the Pima S-7 fuzzless phenotype is complex (Rong *et al.*, 2005).

In conclusion, the Gb fuzzless seed trait is regulated by multiple recessive genetic loci. The role of *MYB25-like_Dt* in regulating fuzz initiation and development is yet to be confirmed by specific silencing of its expression using gene editing, but the expression profile of *MYB25-like_Dt* and genetic analyses of cottons with variable lint and fuzz phenotypes provided quite convincing evidence for *MYB25-like_Dt* being the major fuzz gene in addition to its role in regulating lint development together with *MYB25-like_At*.

## Supplementary data

Supplementary data are available at *JXB* online.

Fig. S1. Schematic of the development of the near isogeneic lines (NILs) used in SNP genotyping and mapping-by-sequencing.

Fig. S2. Alignment of the coding sequences of *MYB25-like*.

Fig. S3. The Cotton SNP63K array based frequency of the Pima S-7 allele of the polymorphic SNPs between Pima S-7 and Sicala 40 in the NILs with normal or reduced fuzz.

Fig. S4. Distribution of the Pima S-7 allele frequency across the 26 cotton chromosomes in the NILs showing fuzzless (RFB2) and segregating fuzz phenotype (RFB1) determined by MBS.

Fig. S5. Representative fuzz phenotype of the FLNS F_2_:F_3_ families.

Fig. S6. Seed fuzz phenotype of representative *G. barbadense* accessions.

Fig. S7. Alignment of the amino acid sequences of *MYB25-like* homoeoalleles from Xu142, Xu142*fl*, TM-1, and Pima S-7.

Fig. S8. Alignments of the promoter sequences of *MYB25-like* from mutant and wild-type lines.

Fig. S9. Distribution of fuzz phenotypes (F_3_ seeds) in the F_2_ populations PX and FLNX by *MYB25-like* homoeoalleles.

Fig. S10. Effects of fuzz associated loci on lint percentage of the FLNS F_2_:F_3_ families.

Table S1. Primers used in the study.

Table S2. Chromosomal distributions of the 5426 polymorphic SNPs between Pima S-7 and Sicala 40 based on the Cotton SNP63K array.

Table S3. Candidate regions identified based on mapping-by-sequencing (MBS) and SNP array association analysis.

Table S4. List of annotated genes in the five chromosomal regions associated with fuzz development.

Supplementary Figure S2

Supplementary Figure S3

Supplementary Figure S4

Supplementary Figure S8

Supplementary Figure S1_S5-S7_S9-S10

Supplementary_Tables_S1-S3

Supplementary_Tables_S4

## References

[CIT0001] Bechere E, Auld DL, Hequet E 2009 Development of ‘naked-tufted’ seed coat mutants for potential use in cotton production. Euphytica 167, 333–339.

[CIT0002] Bechere E, Turley RB, Auld DL, Zeng L 2012 A new fuzzless seed locus in an Upland cotton (*Gossypium hirsutum* L.) mutant. American Journal of Plant Science 3, 799–804.

[CIT0003] Bedon F, Ziolkowski L, Walford SA, Dennis ES, Llewellyn DJ 2014 Members of the MYBMIXTA-like transcription factors may orchestrate the initiation of fiber development in cotton seeds. Frontiers in Plant Science 5, 179. 24860577 10.3389/fpls.2014.00179 PMC4028877

[CIT0004] Boykin JC 2007 Cultivar differences in gin stand energy utilization. Transactions of the ASABE 50, 733–743.

[CIT0005] Byers RL, Harker DB, Yourstone SM, Maughan PJ, Udall JA 2012 Development and mapping of SNP assays in allotetraploid cotton. Theoretical and Applied Genetics 124, 1201–1214. 22252442 10.1007/s00122-011-1780-8 PMC3324690

[CIT0006] Chen W, Yao J, Chu L, Yuan Z, Li Y, Zhang Y 2015 Genetic mapping of the nulliplex-branch gene (*gb_nb1*) in cotton using next-generation sequencing. Theoretical and Applied Genetics 128, 539–547. 25575840 10.1007/s00122-014-2452-2

[CIT0007] Du XM, Pan JJ, Wang RH, Zhang TZ, Shi YZ 2001 Genetic analysis of presence and absence of lint and fuzz in cotton. Plant Breeding 120, 519–522.

[CIT0008] Ellis MH, Stiller WN, Phongkham T, Tate WA, Gillespie VJ, Gapare WJ, Zhu Q-H, Llewellyn DJ, Wilson IW 2016 Molecular mapping of bunchy top disease resistance in *Gossypium hirsutum* L. Euphytica 210, 135–142.

[CIT0009] Endrizzi JE, Ramsay G 1980 Identification of ten chromosome deficiencies in cotton. Journal of Heredity 71, 45–48.

[CIT0010] Endrizzi JE, Taylor T 1968 Cytogenetic studies of *N Lc*_1_*yg*_2_*R*_2_ marker genes and chromosome deficiencies in cotton. Genetics Research 12, 295–304.

[CIT0011] Fang L, Wang Q, Hu Y 2017 Genomic analyses in cotton identify signatures of selection and loci associated with fiber quality and yield traits. Nature Genetics 49, 1089–1098. 28581501 10.1038/ng.3887

[CIT0012] Gapare W, Conaty W, Zhu Q-H, Liu S, Stiller W, Llewellyn D, Wilson I 2017 Genome-wide association study of yield components and fibre quality traits in a cotton germplasm diversity panel. Euphytica 213, 66.

[CIT0013] Hinze LL, Hulse-Kemp AM, Wilson IW 2017 Diversity analysis of cotton (*Gossypium hirsutum* L.) germplasm using the CottonSNP63K Array. BMC Plant Biology 17, 37. 28158969 10.1186/s12870-017-0981-y PMC5291959

[CIT0014] Hu HY, He X, Tu LL, Zhu LF, Zhu ST, Ge ZH, Zhang XL 2016 GhJAZ2 negatively regulates cotton fiber initiation by interacting with the R2R3-MYB transcription factor GhMYB25-like. The Plant Journal 88, 921–935. 27419658 10.1111/tpj.13273

[CIT0015] Huang C, Nie X, Shen C, You C, Li W, Zhao W, Zhang X, Lin Z 2017 *a* Population structure and genetic basis of the agronomic traits of upland cotton in China revealed by a genome-wide association study using high-density SNPs. Plant Biotechnology Journal 15, 1374–1386. 28301713 10.1111/pbi.12722 PMC5633765

[CIT0016] Huang F, Zhu Q-H, Zhu A, Wu X, Xie L, Wu X, Helliwell C, Chaudhury A, Finnegan EJ, Luo M 2017 *b* Mutants in the imprinted *PICKLE RELATED 2* gene suppress seed abortion of *fertilization independent seed* class mutants and paternal excess interploidy crosses in Arabidopsis. The Plant Journal 90, 383–395. 28155248 10.1111/tpj.13500

[CIT0017] Hulse-Kemp AM, Ashrafi H, Stoffel K, Zheng X, Saski CA, Scheffler BE, Fang DD, Chen ZJ, Van Deynze A, Stelly DM 2015 *a* BAC-end sequence-based SNP mining in allotetraploid cotton (*Gossypium*) utilizing resequencing data, phylogenetic inferences, and perspectives for genetic mapping. G3 5, 1095–1105. 25858960 10.1534/g3.115.017749 PMC4478540

[CIT0018] Hulse-Kemp AM, Lemm J, Plieske J 2015 *b* Development of a 63K SNP array for cotton and high-density mapping of intraspecific and interspecific populations of *Gossypium* spp. G3 5, 1187–1209. 25908569 10.1534/g3.115.018416 PMC4478548

[CIT0019] Islam MS, Zeng L, Thyssen GN, Delhom CD, Kim HJ, Li P, Fang DD 2016 Mapping by sequencing in cotton (*Gossypium hirsutum*) line MD52ne identified candidate genes for fiber strength and its related quality attributes. Theoretical and Applied Genetics 129, 1071–1086. 26883043 10.1007/s00122-016-2684-4

[CIT0020] Joshi PC, Wadhwani AM, Johri BM 1967 Morphological and embryological studies of *Gossypium* L. Indian Journal of Agriculture Research 33, 37–93.

[CIT0021] Kearney TH, Harrison GJ 1928 Variation in seed fuzziness on individual plants of Pima cotton. Journal of Agricultural Research 37, 465–472.

[CIT0022] Lang AG 1938 The origin of lint and fuzz hairs of cotton. Journal of Agricultural Research 56, 507–521.

[CIT0023] Lee JJ, Hassan OS, Gao W, Wei NE, Kohel RJ, Chen XY, Payton P, Sze SH, Stelly DM, Chen ZJ 2006 Developmental and gene expression analyses of a cotton naked seed mutant. Planta 223, 418–432. 16254724 10.1007/s00425-005-0098-7

[CIT0024] Li C, Dong Y, Zhao T 2016 Genome-wide SNP linkage mapping and QTL analysis for fiber quality and yield traits in the upland cotton recombinant inbred lines population. Frontiers in Plant Science 7, 1356. 27660632 10.3389/fpls.2016.01356 PMC5014859

[CIT0025] Padmalatha KV, Patil DP, Kumar K 2012 Functional genomics of fuzzless-lintless mutant of *Gossypium hirsutum* L. cv. MCU5 reveal key genes and pathways involved in cotton fibre initiation and elongation. BMC Genomics 13, 624. 23151214 10.1186/1471-2164-13-624 PMC3556503

[CIT0026] Percy RG, Kohel RJ 1999 Qualitative genetics. In: Smith CW, Cothren JT, eds. Cotton: origin, history, technology, and production. New York: John Wiley & Sons, 319–360.

[CIT0027] Romano GB, Taliercio EW, Turley RB, Scheffler JA 2011 Fiber initiation in 18 cultivars and experimental lines of three *Gossypium* species. The Journal of Cotton Science 15, 61–72.

[CIT0028] Rong JK, Pierce GJ, Waghmare VN 2005 Genetic mapping and comparative analysis of seven mutants related to seed fiber development in cotton. Theoretical and Applied Genetics 111, 1137–1146. 16075204 10.1007/s00122-005-0041-0

[CIT0029] Samora PJ, Stelly DJ, Kohel RJ 1994 Localization and mapping of the *Le1* and *Gl2* loci of cotton (*Gossypium hirsutum* L.). Journal of Heredity 85, 152–157.

[CIT0030] Schneeberger K 2014 Using next-generation sequencing to isolate mutant genes from forward genetic screens. Nature Reviews. Genetics 15, 662–676. 10.1038/nrg3745 25139187

[CIT0031] Stewart J 1975 Fiber initiation on the cotton ovule (*Gossypium hirsutum*). American Journal of Botany 62, 723–730.

[CIT0032] Thyssen GN, Fang DD, Turley RB, Florane CB, Li P, Mattison CP, Naoumkina M 2017 A Gly65Val substitution in an actin, GhACT_LI1, disrupts cell polarity and F-actin organization resulting in dwarf, lintless cotton plants. The Plant Journal 90, 111–121. 28078746 10.1111/tpj.13477

[CIT0033] Thyssen GN, Fang DD, Turley RB, Florane C, Li P, Naoumkina M 2014 Next generation genetic mapping of the Ligon-lintless-2 (*Li₂*) locus in upland cotton (*Gossypium hirsutum* L.). Theoretical and Applied Genetics 127, 2183–2192. 25119870 10.1007/s00122-014-2372-1

[CIT0034] Thyssen GN, Fang DD, Turley RB, Florane C, Li P, Naoumkina M 2015 Mapping-by-sequencing of *Ligon-lintless-1* (*Li*_*1*_) reveals a cluster of neighboring genes with correlated expression in developing fibers of Upland cotton (*Gossypium hirsutum* L.). Theoretical and Applied Genetics 128, 1703–1712. 26021293 10.1007/s00122-015-2539-4

[CIT0035] Thyssen GN, Fang DD, Zeng L, Song X, Delhom CD, Condon TL, Li P, Kim HJ 2016 The immature fiber mutant phenotype of cotton (*Gossypium hirsutum*) is linked to a 22-bp frame-shift deletion in a mitochondria targeted pentatricopeptide repeat gene. G3 6, 1627–1633. 27172184 10.1534/g3.116.027649 PMC4889659

[CIT0036] Turley RB, Kloth RH 2002 Identification of a third fuzzless seed locus in upland cotton (*Gossypium hirsutum* L.). The Journal of Heredity 93, 359–364. 12547925 10.1093/jhered/93.5.359

[CIT0037] Turley RB, Kloth RH 2008 The inheritance model for the fiberless trait in upland cotton (*Gossypium hirsutum* L.) line SL1-7-1: variation on a theme. Euphytica 164, 123–132.

[CIT0038] Turley RB, Vaughn KC, Scheffler JA 2007 Lint development and properties of fifteen fuzzless seed lines of Upland cotton (*Gossypium hirsutum* L.). Euphytica 156, 57–65.

[CIT0039] Walford SA, Wu YR, Llewellyn DJ, Dennis ES 2011 GhMYB25-like: a key factor in early cotton fibre development. The Plant Journal 65, 785–797. 21235650 10.1111/j.1365-313X.2010.04464.x

[CIT0040] Wan Q, Guan X, Yang N 2016 Small interfering RNAs from bidirectional transcripts of *GhMML3_A12* regulate cotton fiber development. New Phytologist 210, 1298–1310. 26832840 10.1111/nph.13860

[CIT0041] Wan Q, Zhang H, Ye W, Wu H, Zhang T 2014 Genome-wide transcriptome profiling revealed cotton fuzz fiber development having a similar molecular model as *Arabidopsis* trichome. PLoS ONE 9, e97313. 24823367 10.1371/journal.pone.0097313 PMC4019585

[CIT0042] Wang S, Chen J, Zhang W 2015 Sequence-based ultra-dense genetic and physical maps reveal structural variations of allopolyploid cotton genomes. Genome Biology 16, 108. 26003111 10.1186/s13059-015-0678-1 PMC4469577

[CIT0043] Wang L, Cook A, Patrick JW, Chen XY, Ruan YL 2014 Silencing the vacuolar invertase gene GhVIN1 blocks cotton fiber initiation from the ovule epidermis, probably by suppressing a cohort of regulatory genes via sugar signaling. The Plant Journal 78, 686–696. 24654806 10.1111/tpj.12512

[CIT0044] Wang X, Lu X, Wang J, Wang D, Yin Z, Fan W, Wang S, Ye W 2016 Mining and analysis of SNP in response to salinity stress in upland cotton (*Gossypium hirsutum* L.). PLoS ONE 11, e0158142. 27355327 10.1371/journal.pone.0158142 PMC4927152

[CIT0045] Ware JO 1940 Relation of fuzz pattern to lint in an upland cotton cross. Journal of Heredity 31, 489–496.

[CIT0046] Ware JO, Jenkins WH, Harrell DC 1944 Seed characters and lint production. Journal of Heredity 35, 153–160.

[CIT0047] Wu Y, Machado AC, White RG, Llewellyn DJ, Dennis ES 2006 Expression profiling identifies genes expressed early during lint fibre initiation in cotton. Plant & Cell Physiology 47, 107–127. 16278222 10.1093/pcp/pci228

[CIT0048] Zhang DY, Zhang TZ, Sang ZQ, Guo WZ 2007 Comparative development of lint and fuzz using different cotton fiber-specific developmental mutants in *Gossypium hirsutum*. Journal of Integrative Plant Biology 49, 1038–1046.

[CIT0049] Zhang T, Hu Y, Jiang W 2015 Sequencing of allotetraploid cotton (*Gossypium hirsutum* L. acc. TM-1) provides a resource for fiber improvement. Nature Biotechnology 33, 531–537. 10.1038/nbt.3207 25893781

[CIT0050] Zhang TZ, Pan JJ 1991 Genetic analysis of a fuzzless-lintless mutant in *Gossypium hirsutum* L. Jiangsu Journal of Agricultural Sciences 7, 13–16.

[CIT0051] Zhu Q-H, Fan L, Liu Y, Xu H, Llewellyn D, Wilson I 2013 miR482 regulation of NBS-LRR defense genes during fungal pathogen infection in cotton. PLoS ONE 8, e84390. 24391949 10.1371/journal.pone.0084390 PMC3877274

[CIT0052] Zhu Q-H, Llewellyn D, Wilson I 2014 *a* Use of next-generation sequencing in genomic studies of polyploidcrops: cotton as an example. The Journal of Zhejiang University 40, 355–369.

[CIT0053] Zhu Q-H, Spriggs A, Taylor JM, Llewellyn D, Wilson I 2014 *b* Transcriptome and complexity-reduced, DNA-based identification of intraspecies single-nucleotide polymorphisms in the polyploid *Gossypium hirsutum* L. G3 4, 1893–1905. 25106949 10.1534/g3.114.012542 PMC4199696

[CIT0054] Zhu Q-H, Upadhyaya NM, Gubler F, Helliwell CA 2009 Over-expression of miR172 causes loss of spikelet determinacy and floral organ abnormalities in rice (*Oryza sativa*). BMC Plant Biology 9, 149. 20017947 10.1186/1471-2229-9-149 PMC2803185

[CIT0055] Zhu Q-H, Wilson I, Llewellyn D 2017 Mapping-by-sequencing enabled fast forward genetics in crops with complex genomes. CAB Reviews 12, 16.

[CIT0056] Zhu Q-H, Zhang J, Liu D, Stiller W, Liu D, Zhang Z, Llewellyn D, Wilson I 2016 Integrated mapping and characterization of the gene underlying the okra leaf trait in *Gossypium hirsutum* L. Journal of Experimental Botany 67, 763–774. 26567355 10.1093/jxb/erv494 PMC4737076

