# Supplementary material for: Genetic dissection of the fuzzless seed trait in Gossypium barbadense
Source: J Exp Bot. 2018 Jan 17;69(5):997–1009. doi: 10.1093/jxb/erx459 (PMC6018843; doi:10.1093/jxb/erx459)
Supplement: Supplementary Figure S2 [file erx459_suppl_supplementary_figure_s2.pdf]

Figure S2 Alignment of the coding sequences of *MYB25-like*. Genome-specific single nucleotide polymorphisms (SNPs) are highlighted in pink, the At or Dt sub-genome SNPs among TM-1, Sicala 40 and Pima S-7 are shown in red, SNPs between the At and A2 genome are shown in green, and SNPs between the Dt and D5 genome are shown in blue. Codons with SNPs that changed amino acids are underlined. In *MYB25-like_Dt*, three nucleotides (positions 76, 419, and 965) differ between Pima S-7 and TM-1. Position 76 SNP does not change amino acid.

(A) *MYB25-like*

```
Cotton_A_06163          ATGCAGCAGTCTCCATGTAGCGACAAGGTGGGGTTGAAGAAAGGGCCATGGACTCCAGAA   60
Gh_A12G1503/Pima S-7    ATGCAGCAGTCTCCATGTAGCGACAAGGTGGGGTTGAAGAAAGGGCCATGGACTCCAGAA   60
Gh_A12G1503/TM-1        ATGCAGCAGTCTCCATGTAGCGACAAGGTGGGGTTGAAGAAAGGGCCATGGACTCCAGAA   60
Gh_A12G1503/Sicala 40   ATGCAGCAGTCTCCATGTAGCGACAAGGTGGGGTTGAAGAAAGGGCCATGGACTCCAGAA   60
Gorai.008G179600        ATGCAGCAGTCTCCATGTAGCGACAAGGTGGGGTTGAAGAAAGGGCCATGGACTCCAGAA   60
Gh_D12G1628/Pima S-7    ATGCAGCAGTCTCCATGTAGCGACAAGGTGGTGTTGAAGAAAGGGCCATGGACTCCAGAA   60
Gh_D12G1628/TM-1        ATGCAGCAGTCTCCATGTAGCGACAAGGTGGTGTTGAAGAAAGGGCCATGGACTCCAGAA   60
Gh_D12G1628/Sicala 40   ATGCAGCAGTCTCCATGTAGCGACAAGGTGGTGTTGAAGAAAGGGCCATGGACTCCAGAA   60
                        ******************************* ****************************
```

D12_47603353, KASP marker for the MYB25-like_Dt allele

```
Cotton_A_06163          GAAGACCAAAAACTCTTGTCTTATATTCAAGAACACGGCGGTGGAAGCTGGCGAGGCTTG   120
Gh_A12G1503/Pima S-7    GAAGACCAAAAACTCTCGTCTTATATTCAAGAACACGGCGGTGGAAGCTGGCGAGGCTTG   120
Gh_A12G1503/TM-1        GAAGACCAAAAACTCTTGTCTTATATTCAAGAACACGGCGGTGGAAGCTGGCGAGGCTTG   120
Gh_A12G1503/Sicala 40   GAAGACCAAAAACTCTTGTCTTATATTCAAGAACACGGCGGTGGAAGCTGGCGAGGCTTG   120
Gorai.008G179600        GAAGACCAAAAACTCTTGTCTTATATTCAAGAACACGGCGGTGGAAGCTGGCGCGGCTTG   120
Gh_D12G1628/Pima S-7    GAAGACCAAAAACTCTTGTCTTATATTCAAGAACACGGCGGTGGAAGCTGGCGCGGCTTG   120
Gh_D12G1628/TM-1        GAAGACCAAAAACTCCTGTCTTATATTCAAGAACACGGCGGTGGAAGCTGGCGCGGCTTG   120
Gh_D12G1628/Sicala 40   GAAGACCAAAAACTCCTGTCTTATATTCAAGAACACGGCGGTGGAAGCTGGCGCGGCTTG   120
                        ***************  ************************************ ******

Cotton_A_06163          CCCGCAAAAGCTGGACTTCAAAGATGTGGCAAGAGTTGTAGACTTAGGTGGATTAACTAC   180
Gh_A12G1503/Pima S-7    CCCGCAAAAGCTGGACTTCAAAGATGTGGCAAGAGTTGTAGACTTAGGTGGATTAACTAC   180
Gh_A12G1503/TM-1        CCCGCAAAAGCTGGACTTCAAAGATGTGGCAAGAGTTGTAGACTTAGGTGGATTAACTAC   180
Gh_A12G1503/Sicala 40   CCCGCAAAAGCTGGACTTCAAAGATGTGGCAAGAGTTGTAGACTTAGGTGGATTAACTAC   180
Gorai.008G179600        CCCGCAAAAGCTGGACTTCAAAGATGTGGCAAGAGTTGTAGACTTAGGTGGATTAACTAC   180
Gh_D12G1628/Pima S-7    CCCGCAAAAGCTGGACTTCAAAGATGTGGCAAGAGTTGTAGACTTAGGTGGATTAACTAC   180
Gh_D12G1628/TM-1        CCCGCAAAAGCTGGACTTCAAAGATGTGGCAAGAGTTGTAGACTTAGGTGGATTAACTAC   180
Gh_D12G1628/Sicala 40   CCCGCAAAAGCTGGACTTCAAAGATGTGGCAAGAGTTGTAGACTTAGGTGGATTAACTAC   180
                        ************************************************************

Cotton_A_06163          TTAAGACCAGATATCAAAAGAGGAAAGTTCAGTTCGCAGGAAGAACGAACCATCATTCAA   240
Gh_A12G1503/Pima S-7    TTAAGACCAGATATCAAAAGAGGAAAGTTCAGTTCGCAGGAAGAACGAACCATCATTCAA   240
Gh_A12G1503/TM-1        TTAAGACCAGATATCAAAAGAGGAAAGTTCAGTTCGCAGGAAGAACGAACCATCATTCAA   240
Gh_A12G1503/Sicala 40   TTAAGACCAGATATCAAAAGAGGAAAGTTCAGTTCGCAGGAAGAACGAACCATCATTCAA   240
Gorai.008G179600        TTAAGACCAGATATCAAAAGAGGAAAGTTCAGTTCGCAGGAAGAACGAACCATCATTCAA   240
Gh_D12G1628/Pima S-7    TTAAGACCAGATATCAAAAGAGGAAAGTTCAGTTCGCAGGAAGAACGAACCATCATTCAA   240
Gh_D12G1628/TM-1        TTAAGACCAGATATCAAAAGAGGAAAGTTCAGTTCGCAGGAAGAACGAACCATCATTCAA   240
Gh_D12G1628/Sicala 40   TTAAGACCAGATATCAAAAGAGGAAAGTTCAGTTCGCAGGAAGAACGAACCATCATTCAA   240
                        ************************************************************

Cotton_A_06163          CTCCATGCCCTTCTTGGAAACAGGTGGTCGGCTATTGCGGCTCATTTGCCAAAAAGAACA   300
Gh_A12G1503/Pima S-7    CTCCATGCCCTTCTTGGAAACAGGTGGTCGGCTATTGCGGCTCATTTGCCAAAAAGAACA   300
Gh_A12G1503/TM-1        CTCCACGCCCTTCTTGGAAACAGGTGGTCGGCTATTGCGGCTCATTTGCCAAAAAGAACA   300
Gh_A12G1503/Sicala 40   CTCCACGCCCTTCTTGGAAACAGGTGGTCGGCTATTGCGGCTCATTTGCCAAAAAGAACA   300
Gorai.008G179600        CTCCATGCCCTTCTTGGAAACAGGTGGTCGGCTATTGCGGCTCATTTGCCAAAAAGAACA   300
Gh_D12G1628/Pima S-7    CTCCATGCCCTTCTTGGAAACAGGTGGTCGGCTATTGCGGCTCATTTGCCAAAAAGAACA   300
Gh_D12G1628/TM-1        CTCCATGCCCTTCTTGGAAACAGGTGGTCGGCTATTGCGGCTCATTTGCCAAAAAGAACA   300
Gh_D12G1628/Sicala 40   CTCCATGCCCTTCTTGGAAACAGGTGGTCGGCTATTGCGGCTCATTTGCCAAAAAGAACA   300
                        ***** ******************************************************
```

Position 314, T in Xu142fl, KASP marker for MYB25-like_At of Xu142fl

```
Cotton_A_06163          GACAATGAGATCAAGAACTACTGGAATACACAGTTGAAGAAAAGGTTGACGACGATAGGG   360
Gh_A12G1503/Pima S-7    GACAATGAGATCAAGAACTACTGGAATACACAGTTGAAGAAAAGGTTGACGACGATAGGG   360
Gh_A12G1503/TM-1        GACAATGAGATCAAGAACTACTGGAATACACAGTTGAAGAAAAGGTTGACGACGATAGGG   360
Gh_A12G1503/Sicala 40   GACAATGAGATCAAGAACTACTGGAATACACAGTTGAAGAAAAGGTTGACGACGATAGGG   360
Gorai.008G179600        GACAATGAGATCAAGAACTACTGGAATACACAGTTGAAGAAAAGATTGACCAAGATAGGG   360
Gh_D12G1628/Pima S-7    GACAATGAGATCAAGAACTACTGGAATACACAGTTGAAGAAAAGATTGACCAAGATAGGG   360
Gh_D12G1628/TM-1        GACAATGAGATCAAGAACTACTGGAATACACAGTTGAAGAAAAGATTGACCAAGATAGGG   360
Gh_D12G1628/Sicala 40   GACAATGAGATCAAGAACTACTGGAATACACAGTTGAAGAAAAGATTGACCAAGATAGGG   360
                        ******************************************** ***** * *******
```

08g179600DtF5 & MYB25L_F

```
Cotton_A_06163        ATCGACCCTGCAACTCACAGGCCTAAAACCGATACCCTCGGTTCAACTCCCAAGGATGCC  420
Gh_A12G1503/Pima S-7  ATCGACCCTGCAACTCACAGGCCTAAAACCGATACCCTCGGTTCAACTCCCAAGGATGCC  420
Gh_A12G1503/TM-1      ATCGACCCTGCAACTCACAGGCCTAAAACCGATACCCTCGGTTCAACTCCCAAGGATGCC  420
Gh_A12G1503/Sicala 40 ATCGACCCTGCAACTCACAGGCCTAAAACCGATACCCTCGGTTCAACTCCCAAGGATGCC  420
Gorai.008G179600      ATCGACCCTGCAACTCACAGGCCTAAAACCGATACCCTCGGTTCAACTCCCAAGGATGCC  420
Gh_D12G1628/Pima S-7  ATCGACCCTGCAACTCACAGGCCTAAAACCGATACCCTCGGTTCAACTCCCAAGGATGTC  420
Gh_D12G1628/TM-1      ATCGACCCTGCAACTCACAGGCCTAAAACCGATACCCTCGGTTCAACTCCCAAGGATGCC  420
Gh_D12G1628/Sicala 40 ATCGACCCTGCAACTCACAGGCCTAAAACCGATACCCTCGGTTCAACTCCCAAGGATGTC  420
                      ********************************************************** *

Cotton_A_06163        GCTAACCTTAGCCACATGGCTCAATGGGAGAGTGCTCGGTTAGAAGCTGAAGCTAGATTG  480
Gh_A12G1503/Pima S-7  GCTAACCTTAGCCACATGGCTCAATGGGAGAGTGCTCGGTTAGAAGCTGAAGCTAGATTG  480
Gh_A12G1503/TM-1      GCTAACCTTAGCCACATGGCTCAATGGGAGAGTGCTCGGTTAGAAGCTGAAGCTAGATTG  480
Gh_A12G1503/Sicala 40 GCTAACCTTAGCCACATGGCTCAATGGGAGAGTGCTCGGTTAGAAGCTGAAGCTAGATTG  480
Gorai.008G179600      GCTAACCTTAGCCACATGGCTCAATGGGAGAGTGCTCGGTTAGAAGCTGAAGCTAGATTG  480
Gh_D12G1628/Pima S-7  GCTAACCTTAGCCACATGGCTCAATGGGAGAGTGCTCGGTTAGAAGCTGAAGCTAGATTG  480
Gh_D12G1628/TM-1      GCTAACCTTAGCCACATGGCTCAATGGGAGAGTGCTCGGTTAGAAGCTGAAGCTAGATTG  480
Gh_D12G1628/Sicala 40 GCTAACCTTAGCCACATGGCTCAATGGGAGAGTGCTCGGTTAGAAGCTGAAGCTAGATTG  480
                      ************************************************************

Cotton_A_06163        GTGAGAGAGTCGAAACGAGTTTCAAACCCTCCGCAAAACCAATTTAGGTTCACGTCTTCA  540
Gh_A12G1503/Pima S-7  GTGAGAGAGTCGAAACGAGTTTCAAACCCTCCGCAAAACCAATTTAGGTTCACGTCTTCA  540
Gh_A12G1503/TM-1      GTGAGAGAGTCGAAACGAGTTTCAAACCCTCCGCAAAACCAATTTAGGTTCACGTCTTCA  540
Gh_A12G1503/Sicala 40 GTGAGAGAGTCGAAACGAGTTTCAAACCCTCCGCAAAACCAATTTAGGTTCACGTCTTCA  540
Gorai.008G179600      GTGAGAGAGTCGAAACGAGTTTCAAACCCTCCGCAAAACCAATTTAGGTTCACGTCTTCA  540
Gh_D12G1628/Pima S-7  GTGAGAGAGTCGAAACGAGTTTCAAACCCTCCGCAAAACCAATTTAGGTTCACGTCTTCA  540
Gh_D12G1628/TM-1      GTGAGAGAGTCGAAACGAGTTTCAAACCCTCCGCAAAACCAATTTAGGTTCACGTCTTCA  540
Gh_D12G1628/Sicala 40 GTGAGAGAGTCGAAACGAGTTTCAAACCCTCCGCAAAACCAATTTAGGTTCACGTCTTCA  540
                      ************************************************************

Cotton_A_06163        TCGGCTCCTCCACTGGTAAGCAAAATTGATGTTGGTTTGGCTCATGCTACTAAACCGCAA  600
Gh_A12G1503/Pima S-7  TCGGCTCCTCCACTGGTAAGCAAAATTGATGTTGGTTTGGCTCATGCTACTAAACCGCAA  600
Gh_A12G1503/TM-1      TCGGCTCCTCCACTGGTAAACAAAATTGATGTTGGTTTGGCTCATGCTACTAAACCGCAA  600
Gh_A12G1503/Sicala 40 TCGGCTCCTCCACTGGTAAACAAAATTGATGTTGGTTTGGCTCATGCTACTAAACCGCAA  600
Gorai.008G179600      TCGGCTCCTCCACTGGTAAGCAAAATTGATGTTGGTTTGGCTCATGCTACTAAACCGCAA  600
Gh_D12G1628/Pima S-7  TCGGCTCCTCCACTGGTAAACAAAATTGATGTTGGTTTGGCTCATGCTACTAAACCGCAA  600
Gh_D12G1628/TM-1      TCGGCTCCTCCACTGGTAAACAAAATTGATGTTGGTTTGGCTCATGCTACTAAACCGCAA  600
Gh_D12G1628/Sicala 40 TCTGCTCCTCCACTGGTAAACAAAATTGATGTTGGTTTGGCTCATGCTACTAAACCGCAA  600
                      ******************* ****************************************

Cotton_A_06163        TGCCTCGATGTACTCAAAGCTTGGCAACGTGTAGTCACTGGATTGTTCACTTTCAACACT  660
Gh_A12G1503/Pima S-7  TGCCTCGATGTACTCAAAGCTTGGCAACGTGTAGTCACTGGATTGTTCACTTTCAACACT  660
Gh_A12G1503/TM-1      TGCCTCGATGTACTCAAAGCTTGGCAACGTGTAGTCACTGGATTGTTCACTTTCAACACT  660
Gh_A12G1503/Sicala 40 TGCCTCGATGTACTCAAAGCTTGGCAACGTGTAGTCACTGGATTGTTCACTTTCAACACT  660
Gorai.008G179600      TGCCTCGATGTACTCAAAGCTTGGCAACGTGTAGTCACTGGATTGTTCACTTTCAACACT  660
Gh_D12G1628/Pima S-7  TGCCTCGATGTACTCAAAGCTTGGCAACGTGTAGTCACTGGATTGTTCACTTTCAACACT  660
Gh_D12G1628/TM-1      TGCCTCGATGTACTCAAAGCTTGGCAACGTGTAGTCACTGGATTGTTCACTTTCAACACT  660
Gh_D12G1628/Sicala 40 TGCCTCGATGTACTCAAAGCTTGGCAACGTGTAGTCACTGGATTGTTCACTTTCAACACT  660
                      ************************************************************

Cotton_A_06163        GACAACCTCCAATCTCCAACATCGACGTCGAGCTTCACGGAAAACACGTTACCAATCTCA  720
Gh_A12G1503/Pima S-7  GACAACCTCCAATCTCCAACATCGACGTCGAGCTTCACGGAAAACACGTTACCAATCTCA  720
Gh_A12G1503/TM-1      GACAACCTCCAATCTCCAACATCGACGTCGAGCTTCACGGAAAACACGTTACCAATCTCA  720
Gh_A12G1503/Sicala 40 GACAACCTCCAATCTCCAACATCGACGTCGAGCTTCACGGAAAACACGTTACCAATCTCA  720
Gorai.008G179600      GACAACCTCCAATCTCCAACATCGACGTCGAGCTTCACGGAAAACAGGTTACCAATCTCA  720
Gh_D12G1628/Pima S-7  GACAACCTCCAATCTCCAACATCGACGTCGAGCTTCACGGAAAACAGGTTACCAATCTCA  720
Gh_D12G1628/TM-1      GACAACCTCCAATCTCCAACATCGACGTCGAGCTTCACGGAAAACAGGTTACCAATCTCA  720
Gh_D12G1628/Sicala 40 GACAACCTCCAATCTCCAACATCGACGTCGAGCTTCACGGAAAACAGGTTACCAATCTCA  720
                      ********************************************** *************

Cotton_A_06163        TCTGTCGGGTTCATTGACAGCTTTGTGGGGAACTCAAATAACAGCTGTTGCGGAAATAAT  780
Gh_A12G1503/Pima S-7  TCTGTCGGGTTCATTGACAGCTTTGTGGGGAACTCAAATAACAGCTGTTGCGGAAATAAT  780
Gh_A12G1503/TM-1      TCTGTCGGGTTCATTGACAGCTTTGTGGGGAACTCAAATAACAGCTGTTGCGGAAATAAT  780
Gh_A12G1503/Sicala 40 TCTGTCGGGTTCATTGACAGCTTTGTGGGGAACTCAAATAACAGCTGTTGCGGAAATAAT  780
Gorai.008G179600      TCTGTCGGATTCATTGACAGCTTTTTGGGGAACTCAAATAACAGCTGTTGCGGAAATAAT  780
Gh_D12G1628/Pima S-7  TCTGTCGGATTCATTGACAGCTTTGTGGGGAACTCAAATAACAGCTGTTGCGGAAATAAT  780
Gh_D12G1628/TM-1      TCTGTCGGATTCATTGACAGCTTTGTGGGGAACTCAAATAACAGCTGTTGCGGAAATAAT  780
Gh_D12G1628/Sicala 40 TCTGTCGGATTCATTGACAGCTTTGTGGGGAACTCAAATAACAGCTGTTGCGGAAATAAT  780
                      ******** *************** ***********************************

Cotton_A_06163        TGGGAATGTGTGGAGAAATCGAGCCAAGTTGCTGACTTACAGGAAAGATTGGATAACTCA  840
Gh_A12G1503/Pima S-7  TGGGAATGTGTGGAGAAATCGAGCCAAGTTGCTGAATTACAGGAAAGATTGGATAACTCA  840
Gh_A12G1503/TM-1      TGGGAATGTGTGGAGAAATCGAGCCAAGTTGCTGAATTACAGGAAATATTGGATAACTCA  840
Gh_A12G1503/Sicala 40 TGGGAATGTGTGGAGAAATCGAGCCAAGTTGCTGAATTACAGGAAATATTGGATAACTCA  840
Gorai.008G179600      TGGGAATGTGTGGAGAAATCGAGCCAAGTTGCCGAGTTACAGGAAAGATTGGATAACTCA  840
Gh_D12G1628/Pima S-7  TGGGAATGTGTGGAGAAATCGAGCCAAGTTGCTGAGTTACAGGAAAGATTGGATAACTCA  840
Gh_D12G1628/TM-1      TGGGAATGTGTGGAGAAATCGAGCCAAGTTGCTGAGTTACAGGAAAGATTGGATAACTCA  840
Gh_D12G1628/Sicala 40 TGGGAATGTGTGGAGAAATCGAGCCAAGTTGCTGAGTTACAGGAAAGATTGGATAACTCA  840
                      ******************************** ** ********** *************
```

08g179600_R2

```
Cotton_A_06163         ATGGGGTTGCATGACATATTGGATCTCTCCTCAGAAGATGTATGGTTTCAAGGCTCATAC  900
Gh_A12G1503/Pima S-7   ATGGGGTTGCATGACATATTGGATCTCTCCTCAGAAGATGTATGGTTTCAAGGCTCATAC  900
Gh_A12G1503/TM-1       ATGGGGTTGCATGACATATTGGATCTCTCCTCAGAAGATGTATGGTTTCAAGGCTCATAC  900
Gh_A12G1503/Sicala 40  ATGGGGTTGCATGACATATTGGATCTCTCCTCAGAAGATGTATGGTTTCAAGGCTCATAC  900
Gorai.008G179600       ATGGGGTTGCATGACATATTGGATTTCTCCTCAGACGATGTATGGTTTCAAGGCTCATAC  900
Gh_D12G1628/Pima S-7   ATGGGGTTGCATGACATATTGGATTTCTCCTCAGAAGATGTATGGTTTCAAGGCTCATAC  900
Gh_D12G1628/TM-1       ATGGGGTTGCATGACATATTGGATTTCTCCTCAGAAGATGTATGGTTTCAAGGCTCATAC  900
Gh_D12G1628/Sicala 40  ATGGGGTTGCATGACATATTGGATTTCTCCTCAGAAGATGTATGGTTTCAAGGCTCATAC  900
                       ************************ ********** ************************

Cotton_A_06163         AGGGCGGAAAATATGATGGAAGGGTATTCGGACACGTTAATGGTTTGTGATTCTGGGGAT  960
Gh_A12G1503/Pima S-7   AGGGCGGAAAATATGATGGAAGGGTATTCGGACACGTTAATGGTTTGTGATTCTGGGGAT  960
Gh_A12G1503/TM-1       AGGGCGGAAAATATGATGGAAGGGTATTCGGACACGTTAATGGTTTGTGATTCTGGGGAT  960
Gh_A12G1503/Sicala 40  AGGGCGGAAAATATGATGGAAGGGTATTCGGACACGTTAATGGTTTGTGATTCTGGGGAT  960
Gorai.008G179600       AGGGCGGAAAATATGATGGAAGGGTATTCGGACACGTTAATGGTTTGTGATTCTGGGGAT  960
Gh_D12G1628/Pima S-7   AGGGCGGAAAATATGATGGAAGGGTATTCGGACACGTTAATGGTTTGTGATTCTGGGGAT  960
Gh_D12G1628/TM-1       AGGGCGGAAAATATGATGGAAGGGTATTCGGACACGTTAATGGTTTGTGATTCTGGGGAT  960
Gh_D12G1628/Sicala 40  AGGGCGGAAAATATGATGGAAGGGTATTCGGACACGTTAATGGTTTGTGATTCTGGGGAT  960
                       ************************************************************

Cotton_A_06163         CATCCGAAGAGTTTGTCAATGGAGCCTAGACAAAACTTTAATGTTGGAACAAGTAATGCT  1020
Gh_A12G1503/Pima S-7   CATCCGAAGAGTTTGTCAATGGAGCCTAGACAAAACTTTAATGTTGGAACAAGTAATGCT  1020
Gh_A12G1503/TM-1       CATCCGAAGAGTTTGTCAATGGAGCCTAGACAAAACTTTAATGTTGGAACAAGTAATGCT  1020
Gh_A12G1503/Sicala 40  CATCCGAAGAGTTTGTCAATGGAGCCTAGACAAAACTTTAATGTTGGAACAAGTAATGCT  1020
Gorai.008G179600       CATCAGAAGAGTTTGTCAATGGAGCCTAGACAAAACTTTAATGTTGGAACAAGTAATGCT  1020
Gh_D12G1628/Pima S-7   CATCAGAAGAGTTTGTCAATGGAGCCTAGACAAAACTTTAATGTTGGAACAAGTAATGCT  1020
Gh_D12G1628/TM-1       CATCCGAAGAGTTTGTCAATGGAGCCTAGACAAAACTTTAATGTTGGAACAAGTAATGCT  1020
Gh_D12G1628/Sicala 40  CATCAGAAGAGTTTGTCAATGGAGCCTAGACAAAACTTTAATGTTGGAACAAGTAATGCT  1020
                       **** *******************************************************

Cotton_A_06163         AGTAGTTTCGAAGAAAACAAGAATTACTGGAACAACATCCTTAATTTTGCGAATGCTTCC  1080
Gh_A12G1503/Pima S-7   AGTAGTTTCGAAGAAAACAAGAATTACTGGAACAACATCCTTAATTTTGCGAATGCTTCC  1080
Gh_A12G1503/TM-1       AGTAGTTTCGAAGAAAACAAGAATTACTGGAACAACATCCTTAATTTTGCGAATGCTTCC  1080
Gh_A12G1503/Sicala 40  AGTAGTTTCGAAGAAAACAAGAATTACTGGAACAACATCCTTAATTTTGCGAATGCTTCC  1080
Gorai.008G179600       AGTAGTTTCGAAGAAAACAAGAATTACTGGAACAACATCCTTAATTTTGCGAATGCTTCC  1080
Gh_D12G1628/Pima S-7   AGTAGTTTCGAAGAAAACAAGAATTACTGGAACAACATCCTTAATTTTGCGAATGCTTCC  1080
Gh_D12G1628/TM-1       AGTAGTTTCGAAGAAAACAAGAATTACTGGAACAACATCCTTAATTTTGCGAATGCTTCC  1080
Gh_D12G1628/Sicala 40  AGTAGTTTCGAAGAAAACAAGAATTACTGGAACAACATCCTTAATTTTGCGAATGCTTCC  1080
                       ************************************************************

Cotton_A_06163         CCTTCTGGTTCTTCTGTCTTTTGA          1104
Gh_A12G1503/Pima S-7   CCTTCTGGTTCTTCTGTCTTTTGA          1104
Gh_A12G1503/TM-1       CCTTCTGGTTCTTCTGTCTTTTGA          1104
Gh_A12G1503/Sicala 40  CCTTCTGGTTCTTCTGTCTTTTGA          1104
Gorai.008G179600       CCATCTGGTTCTTCTGTCTTTTGA          1104
Gh_D12G1628/Pima S-7   CCATCTGGTTCTTCTGTCTTTTGA          1104
Gh_D12G1628/TM-1       CCATCTGGTTCTTCTGTCTTTTGA          1104
Gh_D12G1628/Sicala 40  CCATCTGGTTCTTCTGTCTTTTGA          1104
                       ** *********************
```

(B)*MYB25-like* of Xu142 and Xu142*fl* (the SNP in *MYB25-like_At* of Xu142 and Xu142*fl* is highlighted in pink)

```
Xu142_MYB25-like_Dt      ATGCAGCAGTCTCCATGTAGCGACAAGGTGGGGTTGAAGAAAGGGCCATGGACTCCAGAA 60
Xu142fl_MYB25-like_Dt    ATGCAGCAGTCTCCATGTAGCGACAAGGTGGGGTTGAAGAAAGGGCCATGGACTCCAGAA 60
Xu142_MYB25-like_At      ATGCAGCAGTCTCCATGTAGCGACAAGGTGGGGTTGAAGAAAGGGCCATGGACTCCAGAA 60
Xu142fl_MYB25-like_At    ATGCAGCAGTCTCCATGTAGCGACAAGGTGGGGTTGAAGAAAGGGCCATGGACTCCAGAA 60
                         ************************************************************

Xu142_MYB25-like_Dt      GAAGACCAAAAACTCCTGTCTTATATTCAAGAACACGGCGGTGGAAGCTGGCGCGGCTTG 120
Xu142fl_MYB25-like_Dt    GAAGACCAAAAACTCCTGTCTTATATTCAAGAACACGGCGGTGGAAGCTGGCGCGGCTTG 120
Xu142_MYB25-like_At      GAAGACCAAAAACTCTTGTCTTATATTCAAGAACACGGCGGTGGAAGCTGGCGAGGCTTG 120
Xu142fl_MYB25-like_At    GAAGACCAAAAACTCTTGTCTTATATTCAAGAACACGGCGGTGGAAGCTGGCGAGGCTTG 120
                         *************** ************************************* ******

Xu142_MYB25-like_Dt      CCCGCAAAAGCTGGACTTCAAAGATGTGGCAAGAGTTGTAGACTTAGGTGGATTAACTAC 180
Xu142fl_MYB25-like_Dt    CCCGCAAAAGCTGGACTTCAAAGATGTGGCAAGAGTTGTAGACTTAGGTGGATTAACTAC 180
Xu142_MYB25-like_At      CCCGCAAAAGCTGGACTTCAAAGATGTGGCAAGAGTTGTAGACTTAGGTGGATTAACTAC 180
Xu142fl_MYB25-like_At    CCCGCAAAAGCTGGACTTCAAAGATGTGGCAAGAGTTGTAGACTTAGGTGGATTAACTAC 180
                         ************************************************************

Xu142_MYB25-like_Dt      TTAAGACCAGATATCAAAAGAGGAAAGTTCAGTTCGCAGGAAGAACGAACCATCATTCAA 240
Xu142fl_MYB25-like_Dt    TTAAGACCAGATATCAAAAGAGGAAAGTTCAGTTCGCAGGAAGAACGAACCATCATTCAA 240
Xu142_MYB25-like_At      TTAAGACCAGATATCAAAAGAGGAAAGTTCAGTTCGCAGGAAGAACGAACCATCATTCAA 240
Xu142fl_MYB25-like_At    TTAAGACCAGATATCAAAAGAGGAAAGTTCAGTTCGCAGGAAGAACGAACCATCATTCAA 240
                         ************************************************************

Xu142_MYB25-like_Dt      CTCCATGCCCTTCTTGGAAACAGGTGGTCGGCTATTGCGGCTCATTTGCCAAAAAGAACA 300
Xu142fl_MYB25-like_Dt    CTCCATGCCCTTCTTGGAAACAGGTGGTCGGCTATTGCGGCTCATTTGCCAAAAAGAACA 300
Xu142_MYB25-like_At      CTCCACGCCCTTCTTGGAAACAGGTGGTCGGCTATTGCGGCTCATTTGCCAAAAAGAACA 300
Xu142fl_MYB25-like_At    CTCCACGCCCTTCTTGGAAACAGGTGGTCGGCTATTGCGGCTCATTTGCCAAAAAGAACA 300
                         ***** ******************************************************

                         Position 314, KASP marker for MYB25-like_At of Xu142fl

Xu142_MYB25-like_Dt      GACAATGAGATCAAGAACTACTGGAATACACAGTTGAAGAAAAGATTGACCAAGATAGGG 360
Xu142fl_MYB25-like_Dt    GACAATGAGATCAAGAACTACTGGAATACACAGTTGAAGAAAAGATTGACCAAGATAGGG 360
Xu142_MYB25-like_At      GACAATGAGATCAAGAACTACTGGAATACACAGTTGAAGAAAAGGTTGACGACGATAGGG 360
Xu142fl_MYB25-like_At    GACAATGAGATCATGAACTACTGGAATACACAGTTGAAGAAAAGGTTGACGACGATAGGG 360
                         ************* ****************************** ***** * *******

Xu142_MYB25-like_Dt      ATCGACCCTGCAACTCACAGGCCTAAAACCGATACCCTCGGTTCAACTCCCAAGGATGTC 420
Xu142fl_MYB25-like_Dt    ATCGACCCTGCAACTCACAGGCCTAAAACCGATACCCTCGGTTCAACTCCCAAGGATGTC 420
Xu142_MYB25-like_At      ATCGACCCTGCAACTCACAGGCCTAAAACCGATACCCTCGGTTCAACTCCCAAGGATGCC 420
Xu142fl_MYB25-like_At    ATCGACCCTGCAACTCACAGGCCTAAAACCGATACCCTCGGTTCAACTCCCAAGGATGCC 420
                         ********************************************************** *

Xu142_MYB25-like_Dt      GCTAACTTTAGCCACATGGCTCAATGGGAGAGTGCTCGGTTAGAAGCTGAAGCTAGATTG 480
Xu142fl_MYB25-like_Dt    GCTAACTTTAGCCACATGGCTCAATGGGAGAGTGCTCGGTTAGAAGCTGAAGCTAGATTG 480
Xu142_MYB25-like_At      GCTAACCTTAGCCACATGGCTCAATGGGAGAGTGCTCGGTTAGAAGCTGAAGCTAGATTG 480
Xu142fl_MYB25-like_At    GCTAACCTTAGCCACATGGCTCAATGGGAGAGTGCTCGGTTAGAAGCTGAAGCTAGATTG 480
                         ****** *****************************************************

Xu142_MYB25-like_Dt      GTGAGAGAGTCGAAACGAGTTTCAAACCCTCCGCAAAACCAATTTAGGTTCACGTCTTCA 540
Xu142fl_MYB25-like_Dt    GTGAGAGAGTCGAAACGAGTTTCAAACCCTCCGCAAAACCAATTTAGGTTCACGTCTTCA 540
Xu142_MYB25-like_At      GTGAGAGAGTCGAAACGAGTTTCAAACCCTTCGCAAAACCAATTTAGGTTCACGTCTTCA 540
Xu142fl_MYB25-like_At    GTGAGAGAGTCGAAACGAGTTTCAAACCCTTCGCAAAACCAATTTAGGTTCACGTCTTCA 540
                         ****************************** *****************************

Xu142_MYB25-like_Dt      TCTGCTCCTCCACTGGTAAACAAAATTGATGTTGGTTTGGCTCATGCTACTAAACCGCAA 600
Xu142fl_MYB25-like_Dt    TCTGCTCCTCCACTGGTAAACAAAATTGATGTTGGTTTGGCTCATGCTACTAAACCGCAA 600
Xu142_MYB25-like_At      TCGGCTCCTCCACTGGTAAGCAAAATTGATGTTGGTTTGGCTCATGCTACTAAACCGCAA 600
Xu142fl_MYB25-like_At    TCGGCTCCTCCACTGGTAAGCAAAATTGATGTTGGTTTGGCTCATGCTACTAAACCGCAA 600
                         ** **************** ****************************************

Xu142_MYB25-like_Dt      TGCCTCGATGTACTCAAAGCTTGGCAACGTGTAGTCACTGGATTGTTCACTTTCAACACT 660
Xu142fl_MYB25-like_Dt    TGCCTCGATGTACTCAAAGCTTGGCAACGTGTAGTCACTGGATTGTTCACTTTCAACACT 660
Xu142_MYB25-like_At      TGCCTCGATGTACTCAAAGCTTGGCAACGTGTAGTCACTGGATTGTTCACTTTCAACACT 660
Xu142fl_MYB25-like_At    TGCCTCGATGTACTCAAAGCTTGGCAACGTGTAGTCACTGGATTGTTCACTTTCAACACT 660
                         ************************************************************

Xu142_MYB25-like_Dt      GACAACCTCCAATCTCCAACATCGACGTCGAGCTTCACGGAAAACAGGTTACCAATCTCA 720
Xu142fl_MYB25-like_Dt    GACAACCTCCAATCTCCAACATCGACGTCGAGCTTCACGGAAAACAGGTTACCAATCTCA 720
Xu142_MYB25-like_At      GACAACCTCCAATCTCCAACATCGACGTCGAGCTTCACGGAAAACACGTTACCAATCTCA 720
Xu142fl_MYB25-like_At    GACAACCTCCAATCTCCAACATCGACGTCGAGCTTCACGGAAAACACGTTACCAATCTCA 720
                         ********************************************** *************

Xu142_MYB25-like_Dt      TCTGTCGGATTCATTGACAGCTTTGTGGGGAACTCAAATAACAGCTGTTGCGGAAATAAT 780
Xu142fl_MYB25-like_Dt    TCTGTCGGATTCATTGACAGCTTTGTGGGGAACTCAAATAACAGCTGTTGCGGAAATAAT 780
Xu142_MYB25-like_At      TCTGTCGGGTTCATTGACAGCTTTGTGGGGAACTCAAATAACAGCTGTTGCGGAAATAAT 780
Xu142fl_MYB25-like_At    TCTGTCGGGTTCATTGACAGCTTTGTGGGGAACTCAAATAACAGCTGTTGCGGAAATAAT 780
                         ******** ***************************************************

Xu142_MYB25-like_Dt      TGGGAATGTGTGGAGAAATCGAGCCAAGTTGCTGAGTTACAGGAAAGATTGGATAACTCA 840
Xu142fl_MYB25-like_Dt    TGGGAATGTGTGGAGAAATCGAGCCAAGTTGCTGAGTTACAGGAAAGATTGGATAACTCA 840
Xu142_MYB25-like_At      TGGGAATGTGTGGAGAAATCGAGCCAAGTTGCTGAATTACAGGAAAGATTGGATAACTCA 840
Xu142fl_MYB25-like_At    TGGGAATGTGTGGAGAAATCGAGCCAAGTTGCTGAATTACAGGAAAGATTGGATAACTCA 840
                         *********************************** ************************
```

```
Xu142_MYB25-like_Dt        ATGGGGTTGCATGACATATTGGATTTCTCCTCAGAAGATGTATGGTTTCAAGGCTCATAC 900
Xu142fl_MYB25-like_Dt      ATGGGGTTGCATGACATATTGGATTTCTCCTCAGAAGATGTATGGTTTCAAGGCTCATAC 900
Xu142_MYB25-like_At        ATGGGGTTGCATGACATATTGGATCTCTCCTCAGAAGATGTATGGTTTCAAGGCTCATAC 900
Xu142fl_MYB25-like_At      ATGGGGTTGCATGACATATTGGATCTCTCCTCAGAAGATGTATGGTTTCAAGGCTCATAC 900
                           ************************ ***********************************

Xu142_MYB25-like_Dt        AGGGCGGAAAATATGATGGAAGGGTATTCGGACACGTTAATGGTTTGTGATTCTGGGGAT 960
Xu142fl_MYB25-like_Dt      AGGGCGGAAAATATGATGGAAGGGTATTCGGACACGTTAATGGTTTGTGATTCTGGGGAT 960
Xu142_MYB25-like_At        AGGGCGGAAAATATGATGGAAGGGTATTCGGACACGTTAATGGTTTGTGATTCTGGGGAT 960
Xu142fl_MYB25-like_At      AGGGCGGAAAATATGATGGAAGGGTATTCGGACACGTTAATGGTTTGTGATTCTGGGGAT 960
                           ************************************************************

Xu142_MYB25-like_Dt        CATCAGAAGAGTTTGTCAATGGAGCCTAGACAAAACTTTAATGTTGGAACAAGTAATGCT 1020
Xu142fl_MYB25-like_Dt      CATCAGAAGAGTTTGTCAATGGAGCCTAGACAAAACTTTAATGTTGGAACAAGTAATGCT 1020
Xu142_MYB25-like_At        CATCCGAAGAGTTTGTCAATGGAGCCTAGACAAAACTTTAATGTTGGAACAAGTAATGCT 1020
Xu142fl_MYB25-like_At      CATCCGAAGAGTTTGTCAATGGAGCCTAGACAAAACTTTAATGTTGGAACAAGTAATGCT 1020
                           **** *******************************************************

Xu142_MYB25-like_Dt        AGTAGTTTCGAAGAAAACAAGAATTACTGGAACAACATCCTTAATTTTGCGAATGCTTCC 1080
Xu142fl_MYB25-like_Dt      AGTAGTTTCGAAGAAAACAAGAATTACTGGAACAACATCCTTAATTTTGCGAATGCTTCC 1080
Xu142_MYB25-like_At        AGTAGTTTCGAAGAAAACAAGAATTACTGGAACAACATCCTTAATTTTGCGAATGCTTCC 1080
Xu142fl_MYB25-like_At      AGTAGTTTCGAAGAAAACAAGAATTACTGGAACAACATCCTTAATTTTGCGAATGCTTCC 1080
                           ************************************************************

Xu142_MYB25-like_Dt        CCATCTGGTTCTTCTGTCTTTTGA    1104
Xu142fl_MYB25-like_Dt      CCATCTGGTTCTTCTGTCTTTTGA    1104
Xu142_MYB25-like_At        CCTTCTGGTTCTTCTGTCTTTTGA    1104
Xu142fl_MYB25-like_At      CCTTCTGGTTCTTCTGTCTTTTGA    1104
                           ** *********************
```
