# Supplementary material for: Genetic dissection of the fuzzless seed trait in Gossypium barbadense
Source: J Exp Bot. 2018 Jan 17;69(5):997–1009. doi: 10.1093/jxb/erx459 (PMC6018843; doi:10.1093/jxb/erx459)
Supplement: Supplementary Figure S3 [file erx459_suppl_supplementary_figure_s3.pdf]

Figure S3 The Cotton SNP63K array based frequency of the Pima S-7 allele of the polymorphic SNPs between Pima S-7 and Sicala 40 in the NILs with normal or reduced fuzz. Chromosomes A02, A04 and D04 have less than 20 polymorphic SNPs (Table S3) and are not shown. * indicates the potential candidate regions used in association analysis. Locus V represents the locus associated with fuzz development.

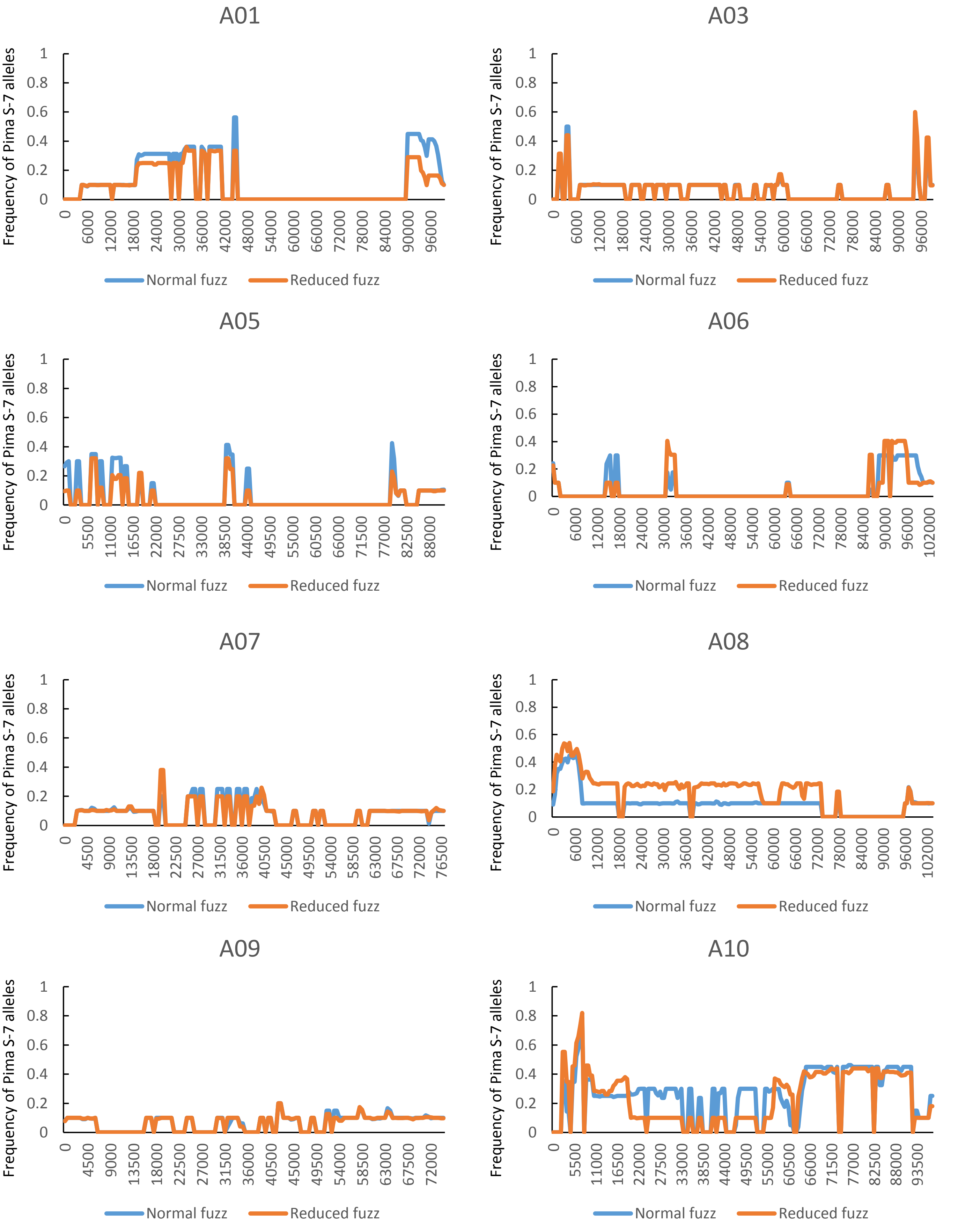

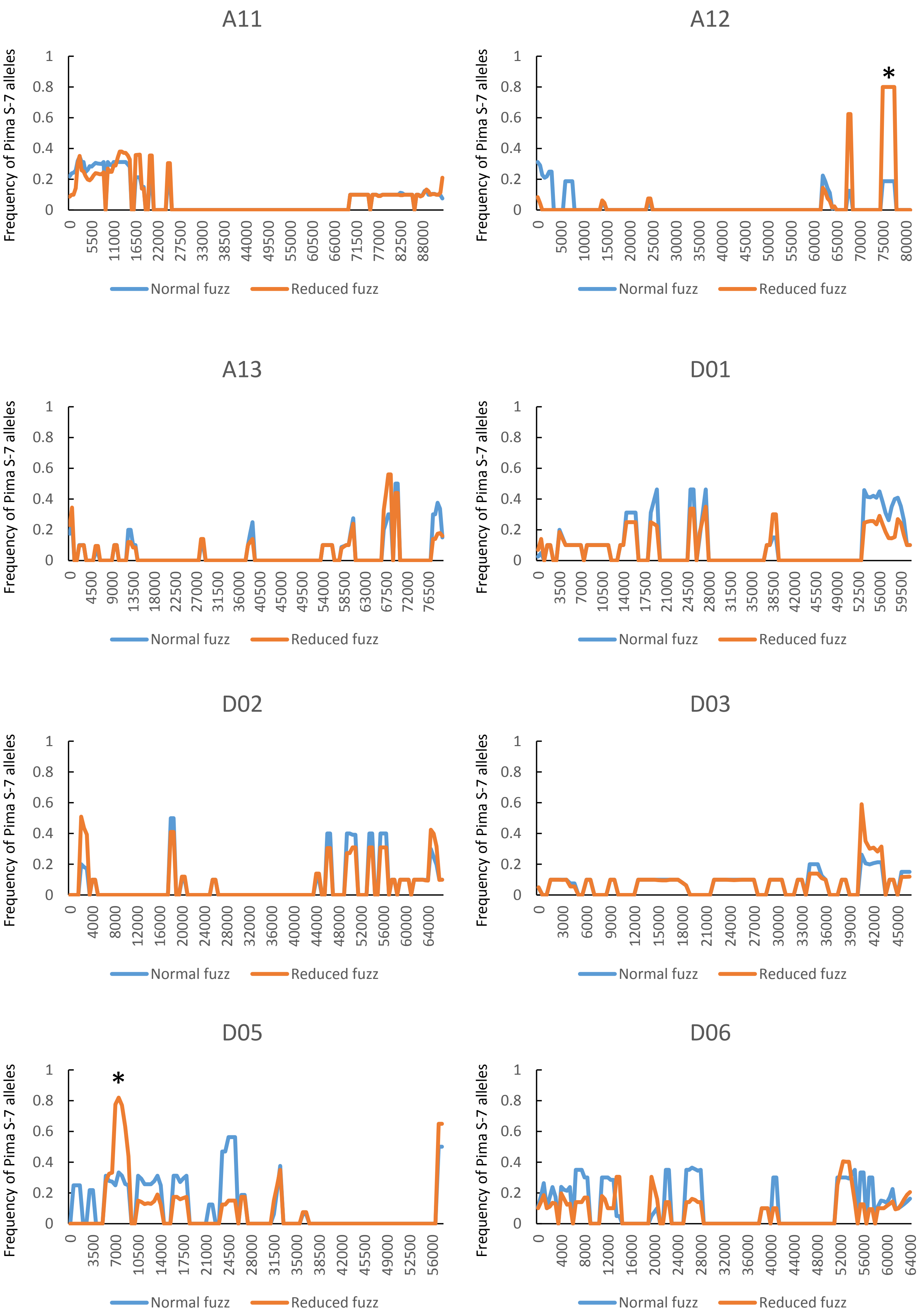
A11
Frequency of Pima S-7 alleles
1
0.8
0.6
0.4
0.2
0
0 5500 11000 16500 22000 27500 33000 38500 44000 49500 55000 60500 66000 71500 77000 82500 88000
Normal fuzz
Reduced fuzz
A12
*
0 5000 10000 15000 20000 25000 30000 35000 40000 45000 50000 55000 60000 65000 70000 75000 80000
A13
0 4500 9000 13500 18000 22500 27000 31500 36000 40500 45000 49500 54000 58500 63000 67500 72000 76500
D01
0 3500 7000 10500 14000 17500 21000 24500 28000 31500 35000 38500 42000 45500 49000 52500 56000 59500
D02
0 4000 8000 12000 16000 20000 24000 28000 32000 36000 40000 44000 48000 52000 56000 60000 64000
D03
0 3000 6000 9000 12000 15000 18000 21000 24000 27000 30000 33000 36000 39000 42000 45000
D05
*
0 3500 7000 10500 14000 17500 21000 24500 28000 31500 35000 38500 42000 45500 49000 52500 56000
D06
0 4000 8000 12000 16000 20000 24000 28000 32000 36000 40000 44000 48000 52000 56000 60000 64000

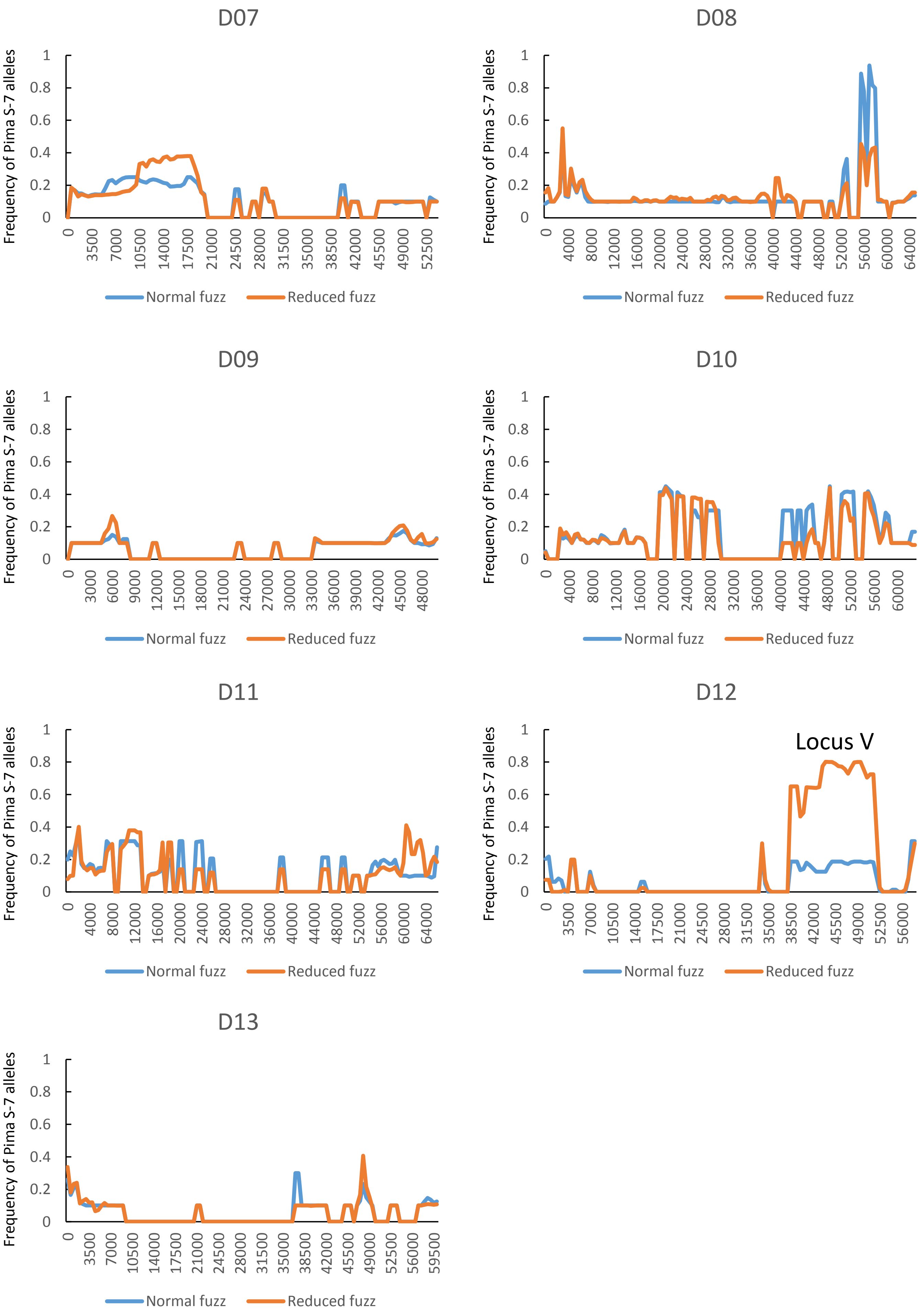
D07
D08
D09
D10
D11
D12
D13
Frequency of Pima S-7 alleles
Normal fuzz
Reduced fuzz
Locus V
