# Supplementary material for: Genetic dissection of the fuzzless seed trait in Gossypium barbadense
Source: J Exp Bot. 2018 Jan 17;69(5):997–1009. doi: 10.1093/jxb/erx459 (PMC6018843; doi:10.1093/jxb/erx459)
Supplement: Supplementary Figure S4 [file erx459_suppl_supplementary_figure_s4.pdf]

Figure S4 Distribution of the Pima S-7 allele frequency across the 26 cotton chromosomes in the NILs showing fuzzless (RFB2) and segregating fuzz phenotype (RFB1). * indicates the potential candidate regions used in association analysis. Locus I, II, III. IV and V represent the five loci associated with fuzz development.

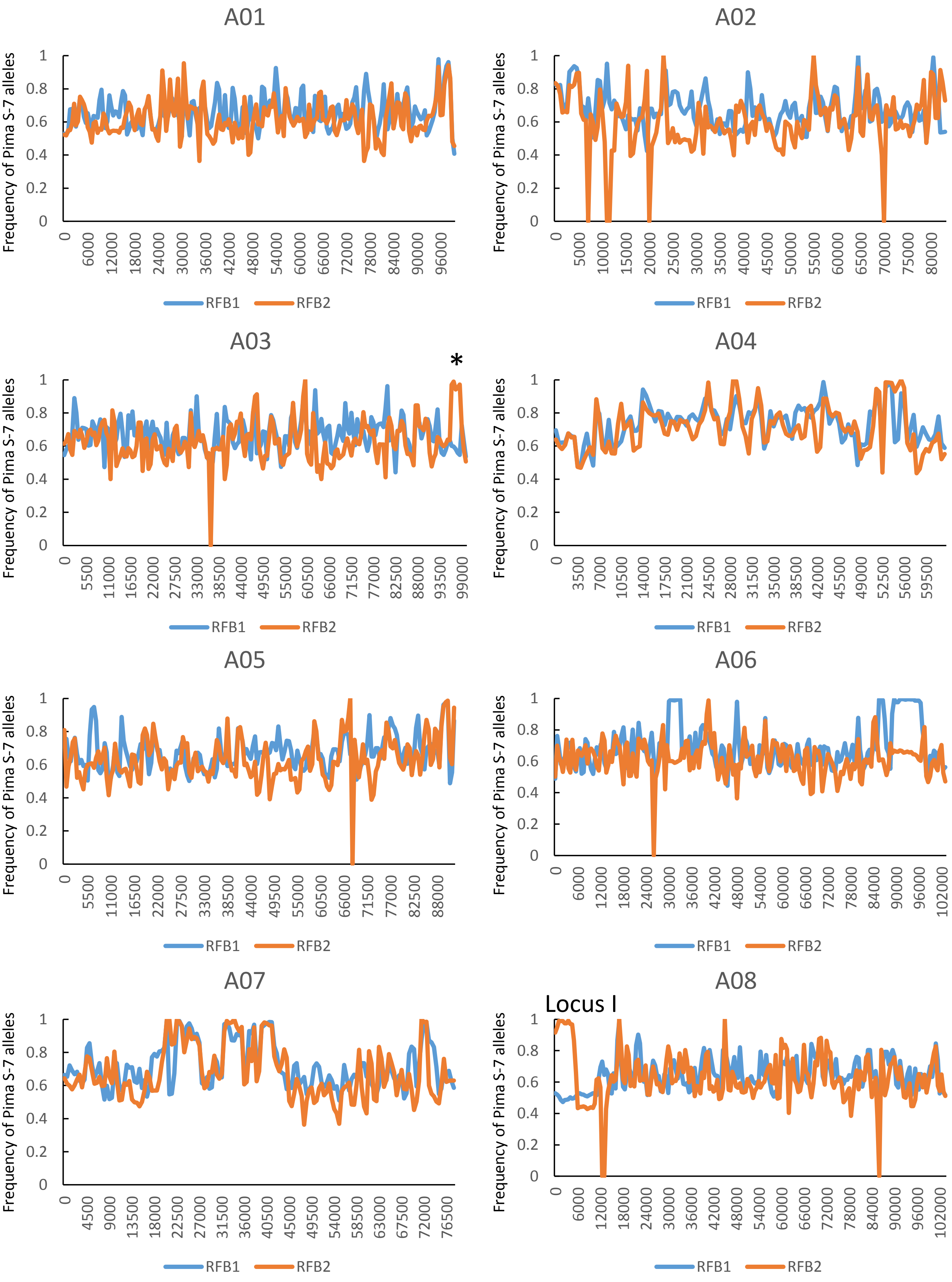

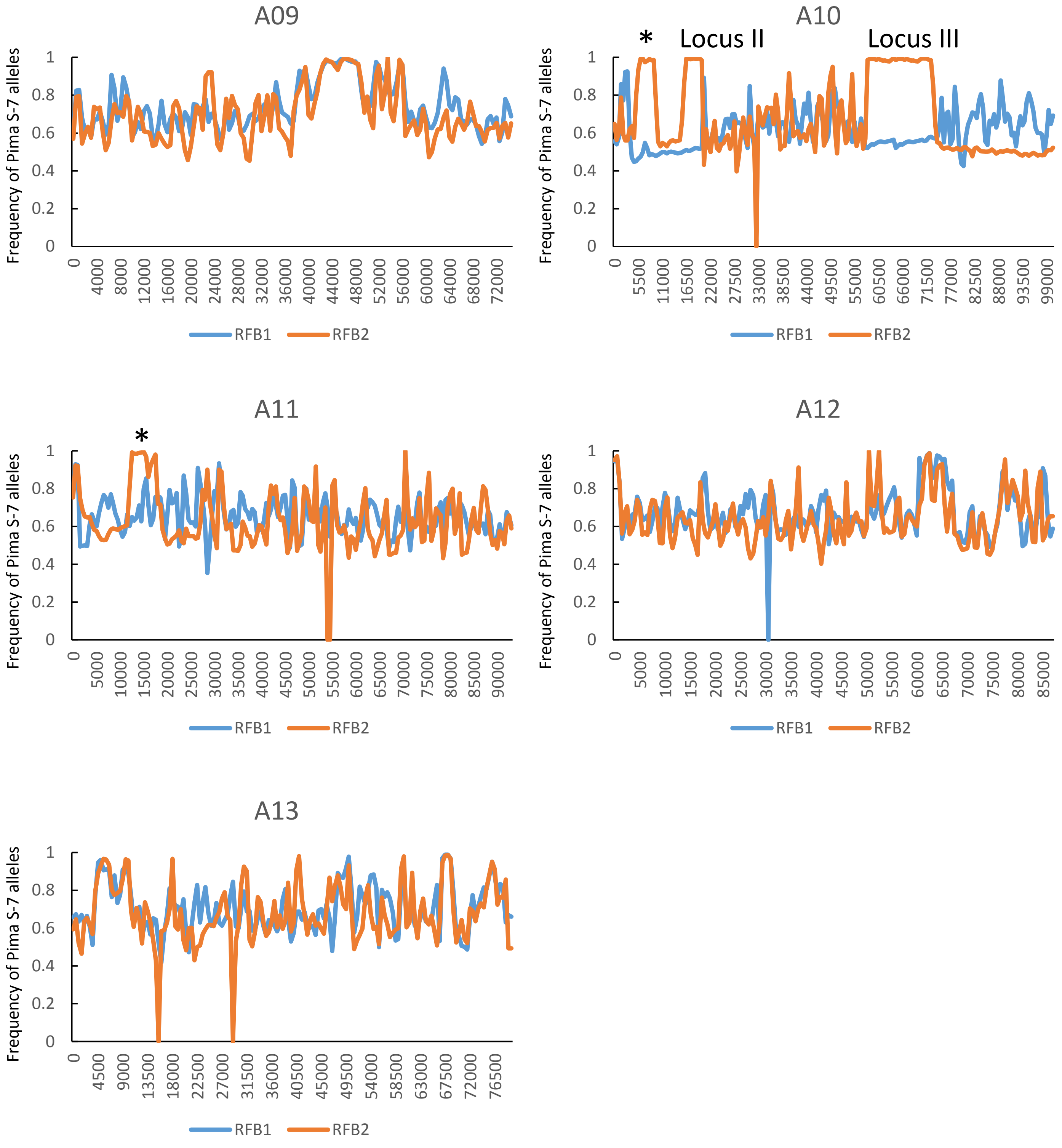
A09
Frequency of Pima S-7 alleles
1
0.8
0.6
0.4
0.2
0
0
4000
8000
12000
16000
20000
24000
28000
32000
36000
40000
44000
48000
52000
56000
60000
64000
68000
72000
RFB1
RFB2
A10
*
Locus II
Locus III
Frequency of Pima S-7 alleles
1
0.8
0.6
0.4
0.2
0
0
5500
11000
16500
22000
27500
33000
38500
44000
49500
55000
60500
66000
71500
77000
82500
88000
93500
99000
RFB1
RFB2
A11
*
Frequency of Pima S-7 alleles
1
0.8
0.6
0.4
0.2
0
0
5000
10000
15000
20000
25000
30000
35000
40000
45000
50000
55000
60000
65000
70000
75000
80000
85000
90000
RFB1
RFB2
A12
Frequency of Pima S-7 alleles
1
0.8
0.6
0.4
0.2
0
0
5000
10000
15000
20000
25000
30000
35000
40000
45000
50000
55000
60000
65000
70000
75000
80000
85000
RFB1
RFB2
A13
Frequency of Pima S-7 alleles
1
0.8
0.6
0.4
0.2
0
0
4500
9000
13500
18000
22500
27000
31500
36000
40500
45000
49500
54000
58500
63000
67500
72000
76500
RFB1
RFB2

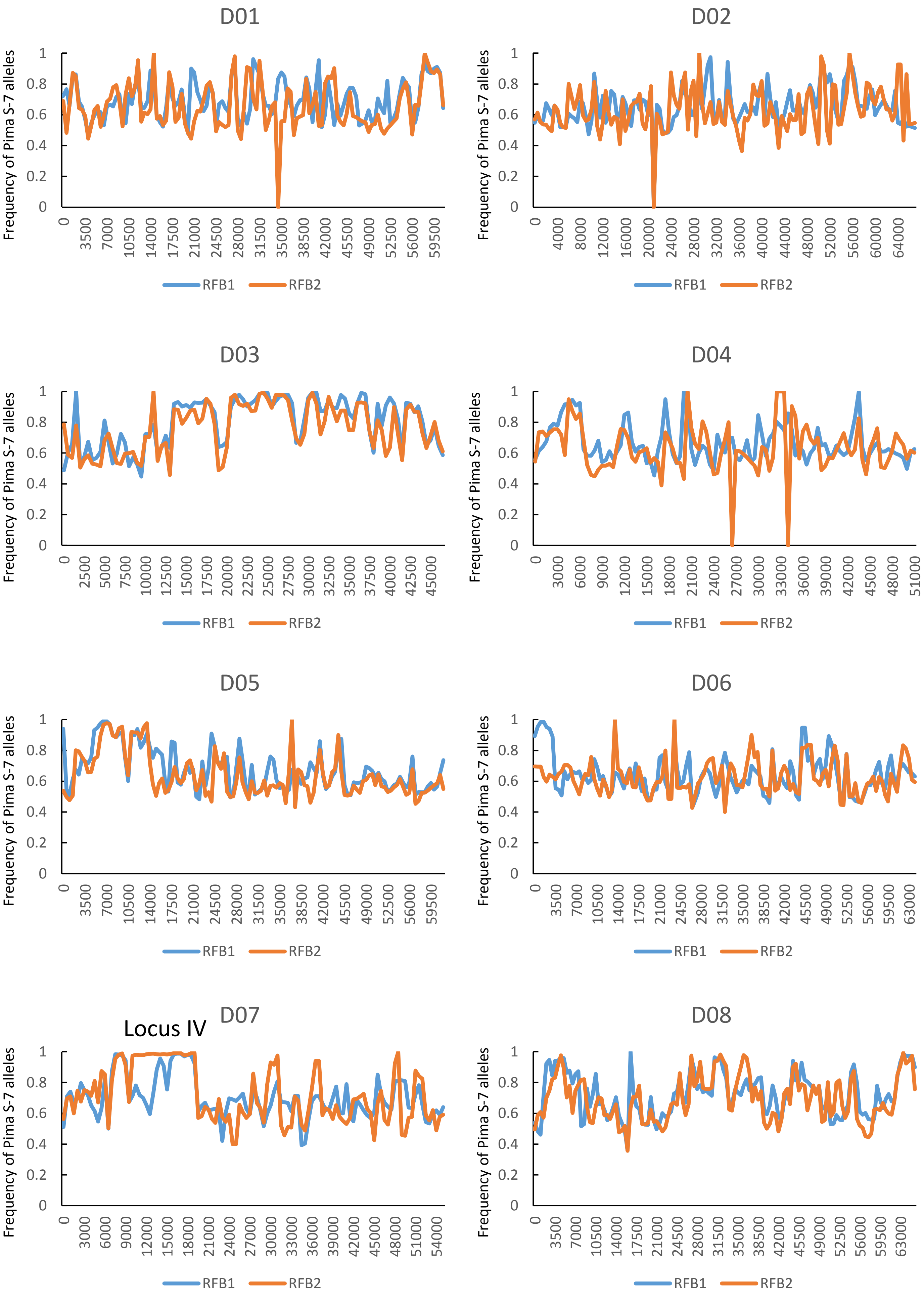
D01
D02
D03
D04
D05
D06
Locus IV
D07
D08
Frequency of Pima S-7 alleles
0
0.2
0.4
0.6
0.8
1
0 3500 7000 10500 14000 17500 21000 24500 28000 31500 35000 38500 42000 45500 49000 52500 56000 59500
0 4000 8000 12000 16000 20000 24000 28000 32000 36000 40000 44000 48000 52000 56000 60000 64000
0 2500 5000 7500 10000 12500 15000 17500 20000 22500 25000 27500 30000 32500 35000 37500 40000 42500 45000
0 3000 6000 9000 12000 15000 18000 21000 24000 27000 30000 33000 36000 39000 42000 45000 48000 51000
0 3500 7000 10500 14000 17500 21000 24500 28000 31500 35000 38500 42000 45500 49000 52500 56000 59500
0 3500 7000 10500 14000 17500 21000 24500 28000 31500 35000 38500 42000 45500 49000 52500 56000 59500 63000
0 3000 6000 9000 12000 15000 18000 21000 24000 27000 30000 33000 36000 39000 42000 45000 48000 51000 54000
0 3500 7000 10500 14000 17500 21000 24500 28000 31500 35000 38500 42000 45500 49000 52500 56000 59500 63000
RFB1
RFB2

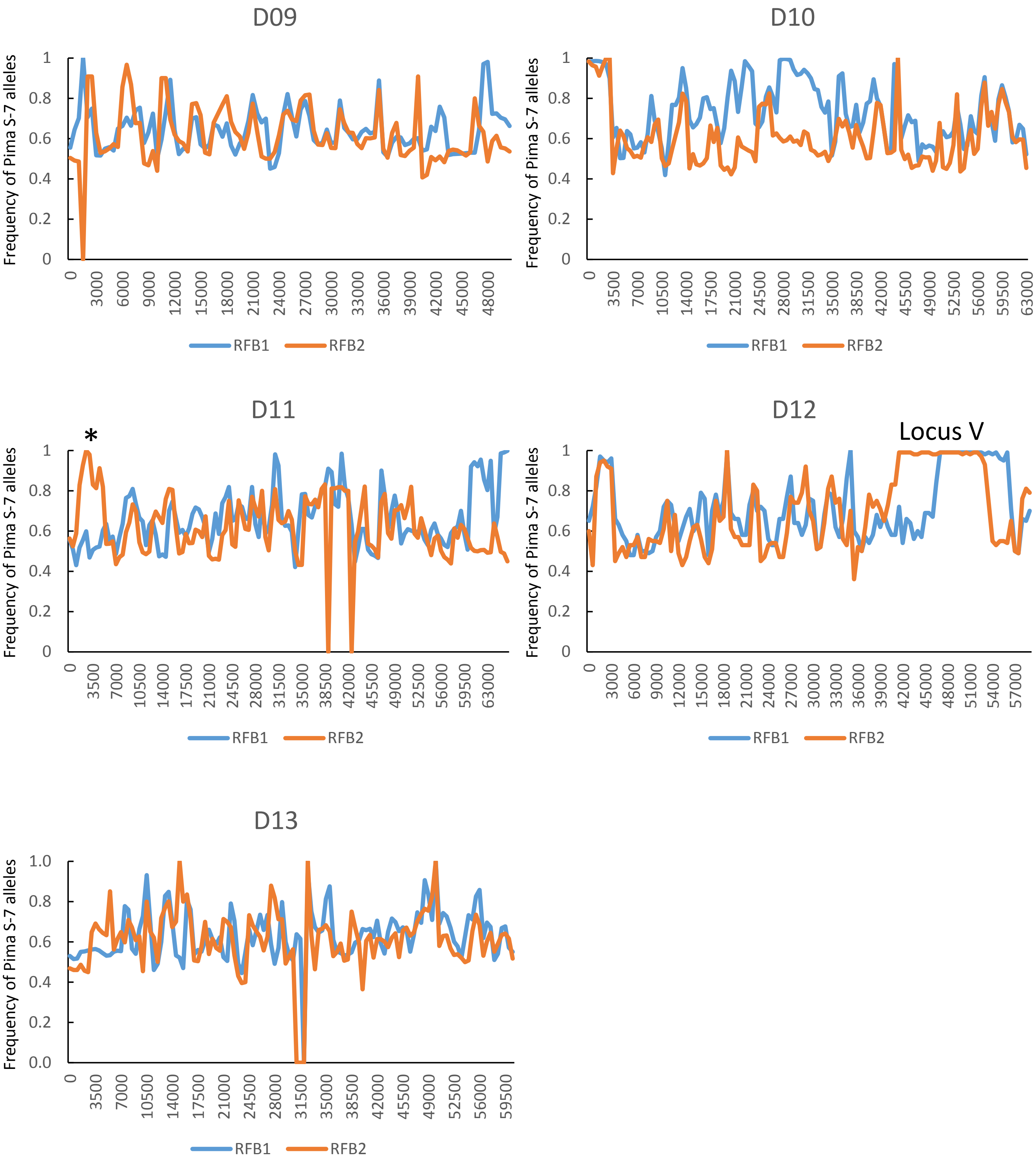
D09
D10
D11
D12
D13
Locus V
*
Frequency of Pima S-7 alleles
1
0.8
0.6
0.4
0.2
0
1.0
0.0
0
3000
6000
9000
12000
15000
18000
21000
24000
27000
30000
33000
36000
39000
42000
45000
48000
3500
7000
10500
14000
17500
24500
28000
31500
35000
38500
45500
49000
52500
56000
59500
63000
51000
54000
57000
5950
RFB1
RFB2
