# Supplementary material for: Genetic dissection of the fuzzless seed trait in Gossypium barbadense
Source: J Exp Bot. 2018 Jan 17;69(5):997–1009. doi: 10.1093/jxb/erx459 (PMC6018843; doi:10.1093/jxb/erx459)
Supplement: Supplementary Figure S8 [file erx459_suppl_supplementary_figure_s8.pdf]

Figure S8 Alignment of the promoter sequences of *MYB25-like*.
cis-acting regulatory elements are underlined and color-coded. MYB binding sites: blue; elements involved in light responsiveness: red; gibberellin-responsive element: black; elements involved in MeJA-responsiveness: green; elements involved in salicylic acid responsiveness: orange. Bases 1-421 of P_Pima S-7_At and P_Sicala 40_At were aligned to chromosome Ca5 of *G. arboreum* rather than chromosome Ca9, on which the A2 genome *MYB25-like* (Cotton-A-06163) was located. Differences between P_Pima S-7_Dt and P_Sicala 40_Dt as well as P_TM-1_Dt are highlighted in yellow and boxed, including three SNPs (one in a putative light responsive element located within the core promoter region and another in a MYB-domain binding site), two small indel (1-bp deletion or insertion), and two deletions that contain two putative light responsive elements.

```
P_A2                --TGAGATACCAATTTTGGGCGTCGCGAGGGTG--CTAATAACTTCCTCGCGCGTAACTA    56
P_PimaS-7_At        TTTGAAGGGATTTTTGTGTTTGTCTCATTTTTCAATCTTTGTAAAAATCCCCCCTTACAT    60
P_Sicala40_At       TTTGAAGGGATTTTTGTGTTTGTCTCATTTTTCAATCTTTGTAAAAATCCCCCCTTACAT    60
P_TM-1_At           TTTGAAGGGATTTTTGTGTTTGTCTCATTTTTCAATCTTTGTAAAAATCCCCCCTTACAT    60
P_D5                ---AAGGAAAAAAAAAAAG-----------------GAAATCTAAACAAAACAATTTTTAA    40
P_PimaS-7_Dt        -----AGGGAAAAAAAAAG-----------------GAAATCAAAACAAAACAATTTTTAA    38
P_Sicala40_Dt       -----AGGGAAAAAAAAAG-----------------GAAATCAAAACAAAACAATTTTTAA    38
P_TM-1_Dt           -----AGGGAAAAAAAAAG-----------------GAAATCAAAACAAAACAATTTTTAA    38
                                     *                     *          *   *

P_A2                ACTCCCGAACCCCAATTTTTCTCTGGACTTTCACGTAGACCTAAAT---TTAGCCTTCTT    113
P_PimaS-7_At        GATTTTGAATGGCTTTTTATAGC-CATCTTTTACATGATTTTCTATTATTTCTTTTTCTA    119
P_Sicala40_At       GATTTTGAATGGCTTTTTATAGC-CATCTTTTACATGATTTTCTATTATTTCTTTTTCTA    119
P_TM-1_At           GATTTTGAATGGCTTTTTATAGC-CATCTTTTACATGATTTTCTATTATTTCTTTTTCTA    119
P_D5                GATGTAAAATGTCTTTTTTGTCGACTTTTTTTTTATAAATAATTTTATTATGATGTTATA    100
P_PimaS-7_Dt        GATGTAAAATGTCTTTTTTGTCTACTTTTTTTTTATAAATAATTTTATTATGATGTTATA    98
P_Sicala40_Dt       GATGTAAAATGTCTTTTTTGTCTACTTTTTTTTTATAAATAATTTTATTATGATGTTATA    98
P_TM-1_Dt           GATGTAAAATGTCTTTTTTGTCTACTTTTTTTTTATAAATAATTTTATTATGATGTTATA    98
                      *    **   *  ***          ***    *         *    *    ** *

P_A2                TTTGTTT-----------TAAAATAATTTTATTAGGTGTCCGATCACAACTATAA-----    157
P_PimaS-7_At        TTTTGTAGGTGGTGGTGGTAGACAATGGATATCAAAAATGGG-----AGAAAAAATGGGG    174
P_Sicala40_At       TTTTGTAGGTGGTGGTGGTAGACAATGGATATCAAAAATGGG-----AGAAAAAATTTT-    173
P_TM-1_At           TTTTGTAGGTGGTGGTGGTAGACAATGGATATCAAAAATGGG-----AGAAAAAATTTT-    173
P_D5                ATTGTA-AAACTTTATTATGAACAATCACCTTTGCATATTCTATCCAAACAAAATAAATA    159
P_PimaS-7_Dt        ATTGTTAAAACTTTATTATGAACAATCACCTTTGCATATTCTATCCAAACAAAATAAATA    158
P_Sicala40_Dt       ATTGTTAAAACTTTATTATGAACAATCACCTTTGCATATTCTATCCAAACAAAATAAATA    158
P_TM-1_Dt           ATTGTTAAAACTTTATTATGAACAATCACCTTTGCATATTCTATCCAAACAAAATAAATA    158
                     **               *  *  *      *      *        *   * *

P_A2                AAA-AGGATCGGTGGCGAC--------------TCCCTTTGTTAATAAAATCGAAAGTTT    202
P_PimaS-7_At        CAAGTGGGAAAGTGGCTAG---GTGGCTAGGGTTTCTTGGTTT--------------TTT    217
P_Sicala40_At       ------------------------------------------------------------    173
P_TM-1_At           ------------------------------------------------------------    173
P_D5                AAATTACTTAATTTTCTTTTCCTTCACTACTTTTTAGTGTTTAAATAAGATAATAAATAT    219
P_PimaS-7_Dt        AAATTACTTAATTTTCTTTTCCTTCACTACTTTTTAGTGTTTAAATAAGATAATAAATAT    218
P_Sicala40_Dt       AAATTACTTAATTTTCTTTTCCTTCACTACTTTTTAGTGTTTAAATAAGATAATAAATAT    218
P_TM-1_Dt           AAATTACTTAATTTTCTTTTCCTTCACTACTTTTTAGTGTTTAAATAAGATAATAAATAT    218

P_A2                GTTTTCAAATGT---TTAATAAATCGCCACAATTAGCGACCAAGCAAAACAAAAATTTTT    259
P_PimaS-7_At        TTTTGGGGTTAGTTTTTTTTTTTTTTTTTTTTTTTTGGGT-TAGGTTTTTGGGGGAGGCTT    276
P_Sicala40_At       ----------------TCTTTTTTTTTATTTTTTTTGGGT-TAGGTTTTTTGGGGAGGCTT    216
P_TM-1_At           ----------------TCTTTTTTTTTATTTTTTTTGGGT-TAGGTTTTTTGGGGAGGCTT    216
P_D5                TATTTACTTTCTTTTTTCTTTGCTTTCCTATCTTTAATT-CAAACAT-A-----------    266
P_PimaS-7_Dt        TATTTACTTTATTTTTTCTTTGCTTTCCTATCTTTAATT-CAAACAT-AGTAGAGGTGTT    276
P_Sicala40_Dt       TATTTACTTTATTTTTTCTTTGCTTTCCTATCTTTAATT-CACACAT-AGTAGAGGTGTT    276
P_TM-1_Dt           TATTTACTTTATTTTTTCTTTGCTTTCCTATCTTTAATT-CACACAT-AGTAGA------    270
                                    *  *   *        **       *

P_A2                TTTACGTCG--CTACAA--GAACATTTAAATATTTT----------------------GC    293
P_PimaS-7_At        AATGGGCTTTTGAATTG------GGTTTAATATTTGGGTTTTGTAATGGGTTTGGGTAGA    330
P_Sicala40_At       AATGGGCTTTTGAATTG------GGTTTAATATTTGGGTTTTGTAATGGGTTTGGGTAGA    270
P_TM-1_At           AATGGGCTTTTGAATTG------GGTTTAATATTTGGGTTTTGTAATGGGTTTGGGTAGA    270
P_D5                ------------------------------------------------------------    266
P_PimaS-7_Dt        TATGGGTTGGGCGGCTC-------------------------------------------    293
P_Sicala40_Dt       TATGGGTTGGGCGGCTCGGCCCTGCTTTCCTATCTTTAATTCAAACAT----AGTAGAGG    332
P_TM-1_Dt           ----------------------------------------------------------GG    272
```

```
P_A2            TATTTATATATTTAAAAACAATAAAAAATCATTTTAACCTCAATAAATTTATAATTTGAT    353
P_PimaS-7_At    TGTAAATGGGTCA------TGGGTTAAGGGTTTTAGTGGGTTTTGAGGTTTGGTTGGGTT    384
P_Sicala40_At   TGTAAATGGGTCA------TGGGTTAAGGGTTTTAGTGGGTTTTGAGGTTTGGTTGGGTT    324
P_TM-1_At       TGTAAATGGGTCA------TGGGTTAAGGGTTTTAGTGGGTTTTGAGGTTTGGTTGGGTT    324
P_D5            ------------------------------------------------------------    266
P_PimaS-7_Dt    ---------------------GGCCCGGCCCAATAGCCCGCCGAAAATATGGAAGGATTT    332
P_Sicala40_Dt   TGTTTATGGGTTGGGCGGCTCGGCCCGGCCCAACAGCCCGCCCGAAATATGGAAGGGT-T    391
P_TM-1_Dt       TGTTTATGGGTTGGGCGGCTCGGCCCGGCCCAACAGCCCGCCCGAAATATGGAAGGGT-T    331

P_A2            ATCTTTTTATTACAACCTCGTTAT--------------AATTTTTTTTACTCTATCCTTT    399
P_PimaS-7_At    TTGATATAATTGGGCCCGGGCAATTTGGGCTTCTACAGAGTATCTCTTGCTT--------    436
P_Sicala40_At   TTGATATAATCGGGCCCAGGCAATTTGGGCTTCTACAGAGTATCTCTTGCTT--------    376
P_TM-1_At       TTGATATAATCGGGCCCAGGCAATTTGGGCTTCTACAGAGTATCTCTTGCTT--------    376
P_D5            ------------------------------------------------------------    266
P_PimaS-7_Dt    TGGATAAAAATATAGGCCCGAAAT------------------------------------    356
P_Sicala40_Dt   TGGATAAAAATATAGGCCCGAAATATGGGCTCGGGCACCACTTTTTTTGCCTGAGCCCGG    451
P_TM-1_Dt       TGGATAAAAATATAGGCCCGAAATATGGGCTCGGGCACCACTTTTTTTGCCTGAGCCCGG    391

P_A2            ATCATAAATATCAAATTGAGTATCTCTTGCTTTCCTTGAAAAGTTAAATTTTTTATTTAT    459
P_PimaS-7_At    ----TCCTTGAAAAGT----------TAAAT------------------TTTTTATTTAT    464
P_Sicala40_At   ----TCCTTGAAAAGT----------TAAAT------------------TTTTTATTTAT    404
P_TM-1_At       ----TCCTTGAAAAGT----------TAAAT------------------TTTTTATTTAT    404
P_D5            ------------------------------------------------------------    266
P_PimaS-7_Dt    -----------------------------------ATGGGCTCGGGCACCACTTTTTTTG    381
P_Sicala40_Dt   C-CCGGCCCGATATAA----------TACATATATTTGGGCTCGGGCACCACTTTTTTTG    500
P_TM-1_Dt       ------------------------------------------------------------    391

P_A2            CCCGATTCAAGAGAAGTTAAAGAAAGGAAAAAGAAAATCAAAACAAAACAACTTTTAAGA    519
P_PimaS-7_At    CCCGATTCAAGAGAAGTTAAAGAAAGGAAAAAGAAAATCAAAACAAAACAACTTTTAAGA    524
P_Sicala40_At   CCCGATTCAAGAGAAGTTAAAGAAAGGAAAAAGAAAATCAAAACAAAACAACTTTTAAGA    464
P_TM-1_At       CCCGATTCAAGAGAAGTTAAAGAAAGGAAAAAGAAAATCAAAACAAAACAACTTTTAAGA    464
P_D5            ------------------------------------------------------------    266
P_PimaS-7_Dt    CCCGAGCCCGGCCCGGCCCGATATA--ATAAATATATTTATTTTTTAAAATTTTTTTAAT    439
P_Sicala40_Dt   CCCGAGCCCGGCCCGGCCCGATATA--ATAAATATATTTATTTTTTAAAATTTTTTTAAT    558
P_TM-1_Dt       -----------CCCGGCCCGATATA--ATAAATATATTTATTTTTTAAAATTTTTTTAAT    438

P_A2            TGTATAATGTCTTTTTT-------------------------------------GTCAAC    542
P_PimaS-7_At    TGTATAATGTCT---------TTTT-----------------------------TGTCAA    546
P_Sicala40_At   TGTATAATGTCT---------TTTT-----------------------------TGTCAA    486
P_TM-1_At       TGTATAATGTCT---------TTTT-----------------------------TGTCAA    486
P_D5            ------------------------------------------------------------    266
P_PimaS-7_Dt    TTTAAAATATTTTTAAAATACTTTTTTATTATTTTTTAATTTTAAAATTTTTTTAAAATA    499
P_Sicala40_Dt   TTTAAAATATTTTTAAAATACTTTTTTATTATTTTTTAATTTTAAAATTTTTTTAAAATA    618
P_TM-1_Dt       TTTAAAATATTTTTAAAATACTTTTTTATTATTTTTTAATTTTAAATTTTTTTTAAAATA    498

P_A2            TTTTTTTTTTTTTTATAAATAATTTTATGATGACGTTATAATTGTAAAACTTTATTATGA    602
P_PimaS-7_At    CTTT--TT--TTTTATAAATAATTTTATGATGACGTTATAATTGTAAAACTTTATTATGA    602
P_Sicala40_At   CTTT--TTTTTTTTATAAATAATTTTATGATGACGTTATAATTGTAAAACTTTATTATGA    544
P_TM-1_At       CTTT--TTTTTTTTATAAATAATTTTATGATGACGTTATAATTGTAAAACTTTATTATGA    544
P_D5            ------------------------------------------------------------    266
P_PimaS-7_Dt    CTTTTTTTAATTTTTAAAATAAATTTTTGGTATT--TATTAAAAAATGGGCCGAGTCGAG    557
P_Sicala40_Dt   CTTTTTTTAATTTTTAAAATAAATTTTTGGTATT--TATTAAAAAATGGGCCGAGTCGGG    676
P_TM-1_Dt       CTTTTTTTAATTTTTAAAATAAATTTTTGGTATT--TATTAAAAAATGGGCCGAGTCGGG    556

P_A2            ACAATCATTTCCTTTGCATATTCTATCCAAACAAAAT-AAATAAAATTACTTAATTTTCT    661
P_PimaS-7_At    ACAATCATTTCCTTTGCATATTCTATCCAAACA-AAATAAATAAAATTACTTAATTTTCT    661
P_Sicala40_At   ACAATCATTTCCTTTGCATATTCTATCCAAACA-AAATAAATAAAATTACTTAATTTTCT    603
P_TM-1_At       ACAATCATTTCCTTTGCATATTCTATCCAAACA-AAATAAATAAAATTACTTAATTTTCT    603
P_D5            ------------------------------------------------------------    266
P_PimaS-7_Dt    CCAGGCCTGGGCTTATGCTTTTTTCCCGGGCCGGGCCTGGGCAAAATTTTAGGCCCATAT    617
P_Sicala40_Dt   CCAGGCCTGGGCTTATGCTTTTTTCCCGGGCCGGGCCTGGGCAAAATTTTAGGCCCATAT    736
P_TM-1_Dt       CCAGGCCTGGGCTTATGCTTTTTTCCCGGGCCGGGCCTGGGCAAAATTTTAGGCCCATAT    616
```

MYB binding site differs between Pima S-7 and Sicala 40

```
P_A2              TTTC---------------------------CTTCACTAATTTTTAGTGTTTAAA-----     689
P_PimaS-7_At      TTTC---------------------------CTTCACTAATTTTTAGTGTTTAAATAAGA     694
P_Sicala40_At     TTTC---------------------------CTTCACTAATTTTTAGTGTTTAAATAAGA     636
P_TM-1_At         TTTC---------------------------CTTCACTAATTTTTAGTGTTTAAATAAGA     636
P_D5              ------------------------------------------------------------     266
P_PimaS-7_Dt      TTTGGGCCGGGCCGGGGCCGGGCCTAGGACCCGGGCCAAATT-TTTATGGGCCCGGCCCA     676
P_Sicala40_Dt     TTTGGGCCGGGCCGGGGCCGGGCCTAGGACTCGGCCCAAATTTTTTATGGGCCCGGCCCA     796
P_TM-1_Dt         TTTGGGCCGGGCCGGGGCCGGGCCTAGGACTCGGGCCAAATTTTTTATGGGCCCGGCCCA     676

P_A2              ---------------TAAGATAATAAATATTTTTTACTTTCTTTTTTCTTTGCTTTCCTA     734
P_PimaS-7_At      T--------------------AATAAATATTATTTACTTTCTTTTTTCTTTGCTTTCCTA     734
P_Sicala40_At     T--------------------AATAAATATTATTTACTTTCTTTTTTCTTTGCTTTCCTA     676
P_TM-1_At         T--------------------AATAAATATTATTTACTTTCTTTTTTCTTTGCTTTCCTA     676
P_D5              ------------------------------------------------------------     266
P_PimaS-7_Dt      AACCCAGCCCGGCCCGGCCCAAACCCAGCCCGGCCTGGCCCGAACCCGGCCCGGCCCATG     736
P_Sicala40_Dt     AACCCAGCCCGGCCTGGCCCAAACCCGGCCCGGCCTGGCCCGAACCCGGCCCGGCCCATG     856
P_TM-1_Dt         AACCCAGCCCGGCCTGGCCCAAACCCGGCCCGGCCTGGCCCGAACCCGGCCCGGCCCATG     736

P_A2              TCTTTAATTCAAACATAGTGTTAGAAAAGAAAATTAAATTCTTAAAGAAGGGTATAATGG     794
P_PimaS-7_At      TCTTTAATTCAAACATAGTGTTAGAAAAGAAAATTAAATTCTTAAAGAAGGGTATAATGG     794
P_Sicala40_At     TCTTTAATTCAAACATAGTGTTAGAAAAGAAAATTAAATTCTTAAAGAAGGGTATAATGG     736
P_TM-1_At         TCTTTAATTCAAACATAGTGTTAGAAAAGAAAATTAAATTCTTAAAGAAGGGTATAATGG     736
P_D5              -----------------GTGTTAGAAAAGAAAATTATATTCTTAAACAAGGGTATAATGG     309
P_PimaS-7_Dt      AGCACCTCTAAAACATAGTGTTAGAAAAGAAAATTAAATTCTTAAAGAAGGGTATAATGG     796
P_Sicala40_Dt     AGCACCTCTAAAACATAGTGTTAGAAAAGAAAATTAAATTCTTAAAGAAGGGTATAATGG     916
P_TM-1_Dt         AGCACCTCTAAAACATAGTGTTAGAAAAGAAAATTAAATTCTTAAAGAAGGGTATAATGG     796
                                   ******************* ********* *************

P_A2              TCATATAGGCTTATGCAATATTTATTAACACTGTAAGCCTATAAAACGCAGTTATATATC     854
P_PimaS-7_At      TCATATAGGCTTATGCAATATTTATTAACACTGTAAGCCTATAAAACGCAGTTATATATC     854
P_Sicala40_At     TCATATAGGCTTATGCAATATTTATTAACACTGTAAGCCTATAAAACGCAGTTATATATC     796
P_TM-1_At         TCATATAGGCTTATGCAATATTTATTAACACTGTAAGCCTATAAAACGCAGTTATATATC     796
P_D5              TCATATAGGCTTAAGTAATATTTAT-TACACTGTAAGCCTATAAAACGCACTTATATATC     368
P_PimaS-7_Dt      TCATATAGGCTTAAGTAATATTTAT-TAGACTGTAAGCCTATAAAACGCACTTATATATC     855
P_Sicala40_Dt     TCATATAGGCTTAAGTAATATTTAT-TAGACTGTAAGCCTATAAAACGCACTTATATATC     975
P_TM-1_Dt         TCATATAGGCTTAAGTAATATTTAT-TAGACTGTAAGCCTATAAAACGCACTTATATATC     855
                  ************* * *********  * ********************* *********

P_A2              TACTTTAAATTAACCAACTCTACCCACATTTTCGTTGCTTTCAACTGCGTATAATCTCTC     914
P_PimaS-7_At      TACTTTAAATTAACCAACTCTACCCACATTTTCGTTGCTTTCAACTGCGTATAATCTCTC     914
P_Sicala40_At     TACTTTAAATTAACCAACTCTACCCACATTTTCGTTGCTTTCAACTGCGTATAATCTCTC     856
P_TM-1_At         TACTTTAAATTAACCAACTCTACCCACATTTTCGTTGCTTTCAACTGCGTATAATCTCTC     856
P_D5              TACTTTAAATTAACCAACTCTACCCACATTTTCGTTGCTTTCAACTGCGTATAATATCTC     428
P_PimaS-7_Dt      TACTTTAAATTAACCAATTCTACCCACATTTTCGTTGCTTTCAACTGCGTATAATATCTC     915
P_Sicala40_Dt     TACTTTAAATTAACCAATTCTACCCACATTTTCGTTGCTTTCAACTGCGTATAATATCTC     1035
P_TM-1_Dt         TACTTTAAATTAACCAATTCTACCCACATTTTCGTTGCTTTCAACTGCGTATAATATCTC     915
                  ***************** ************************************* ****

P_A2              TTTCTCTATCATCTCTTTATCATTGTCTGTTTTCAAATTAAGGAACCAAATTCATTGAGG     974
P_PimaS-7_At      TTTCTCTATCATCTCTTTATCATTGTCTGTTTTCAAATTAAGGAACCAAATTCATTGAGG     974
P_Sicala40_At     TTTCTCTATCATCTCTTTATCATTGTCTGTTTTCAAATTAAGGAACCAAATTCATTGAGG     916
P_TM-1_At         TTTCTCTATCATCTCTTTATCATTGTCTGTTTTCAAATTAAGGAACCAAATTCATTGAGG     916
P_D5              ----TCTATCATCTCTTTATCATTGTCTGTTTTCAAATTAAGGAACAAAATTCATTGAGG     484
P_PimaS-7_Dt      T--CTCTATCATCTCTTTATCATTGTCTGTTTTCAAATTAAGGAACAAAATTCATTGAGG     973
P_Sicala40_Dt     T--CTCTATCATCTATTTATCATTGTCTGTTTTCAAATTAAGGAACAAAATTCATTGAGG     1093
P_TM-1_Dt         T--CTCTATCATCTATTTATCATTGTCTGTTTTCAAATTAAGGAACAAAATTCATTGAGG     973
                      ********** ****************************** **************

P_A2              ATTCGGCCTGGCTTGA-TTCAAAGAACATG         1003
P_PimaS-7_At      ATTCGGCCTGGCTTGA-TTCAAAGAACATG         1003
P_Sicala40_At     ATTCGGCCTGGCTTGA-TTCAAAGAACATG         945
P_TM-1_At         ATTCGGCCTGGCTTGA-TTCAAAGAACATG         945
P_D5              ATTCGGCCTGGCTTGACTTCAAAGAACATG         514
P_PimaS-7_Dt      ATTCGGCCTGGCTTGACTTCAAAGAACATG         1003
P_Sicala40_Dt     ATTCGGCCTGGCTTGACTTCAAAGAACATG         1123
P_TM-1_Dt         ATTCGGCCTGGCTTGACTTCAAAGAACATG         1003
                  **************** *  **  *
```

MYB binding site differs between At and Dt
