# Supplementary material for: Genetic dissection of the fuzzless seed trait in Gossypium barbadense
Source: J Exp Bot. 2018 Jan 17;69(5):997–1009. doi: 10.1093/jxb/erx459 (PMC6018843; doi:10.1093/jxb/erx459)
Supplement: Supplementary Figure S1_S5-S7_S9-S10 [file erx459_suppl_supplementary_figure_s1_s5-s7_s9-s10.pdf]

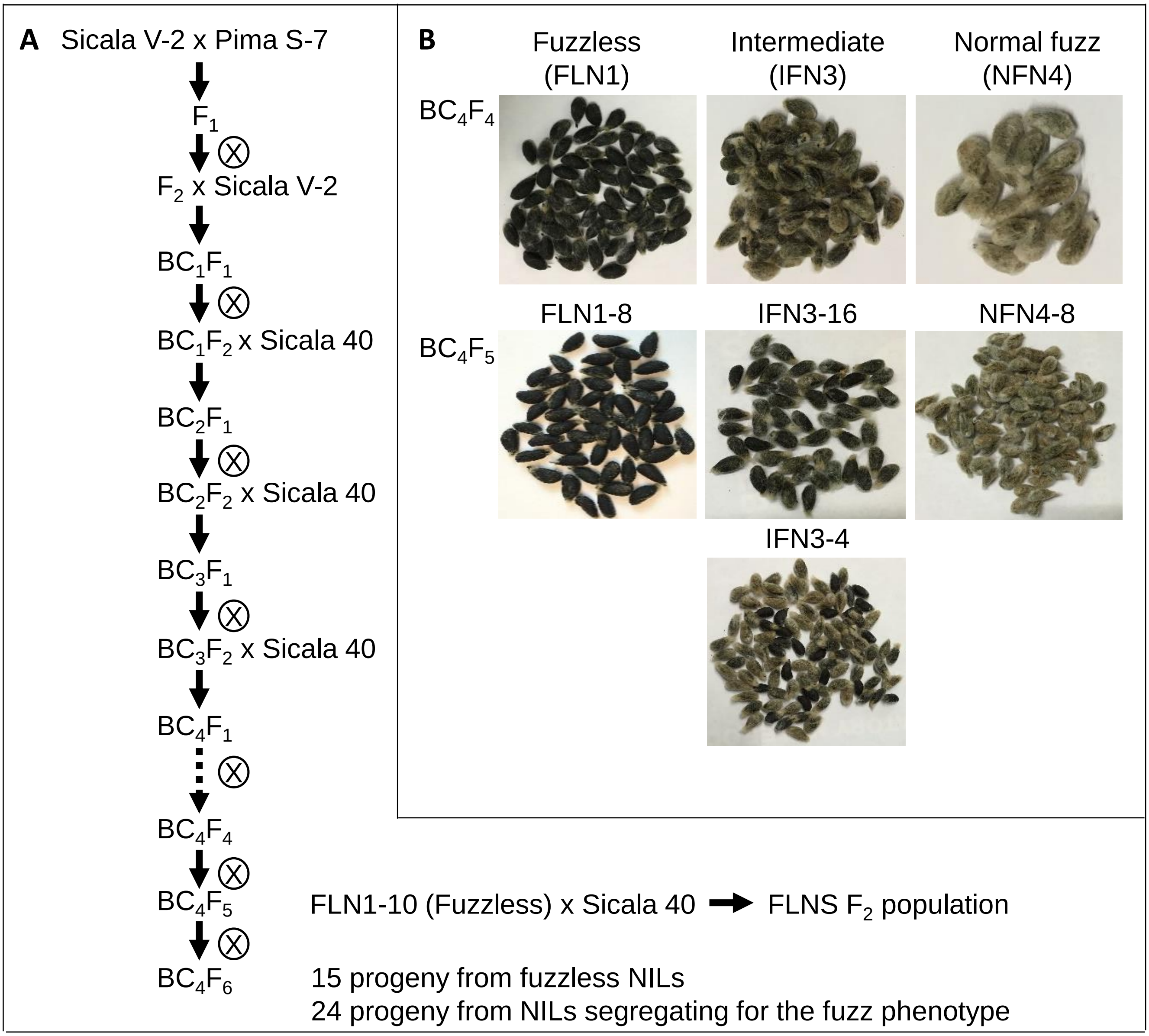

Figure S1 Schematic of the development of the near isogeneic lines (NILs) used in SNP genotyping and mapping-by-sequencing. (A) The BC_1F_1 was selfed and fuzzless BC_1F_2 was used in further backcrosses, which were repeated three more times. After several generations of selfing, BC_4F_4 and BC_4F_5 NILs showing normal and reduced fuzz (including fuzzless and intermediate) were genotyped using the Cotton SNP63K array. BC_4F_5 progeny derived from BC_4F_4 NILs homozygous for normal fuzz (NFN4) or fuzzless (FLN1) phenotype were used for the collection of outer integuments for gene expression analysis. BC_4F_6 progeny derived from fuzzless and fuzzy seeded NILs were used in mapping-by-sequencing. (B) Fuzz phenotype of BC_4F_4 NILs with fuzzless (FLN1), intermediate (IFN3) and normal fuzz (NFN4) seeds, and their representative BC_4F_5 progeny.

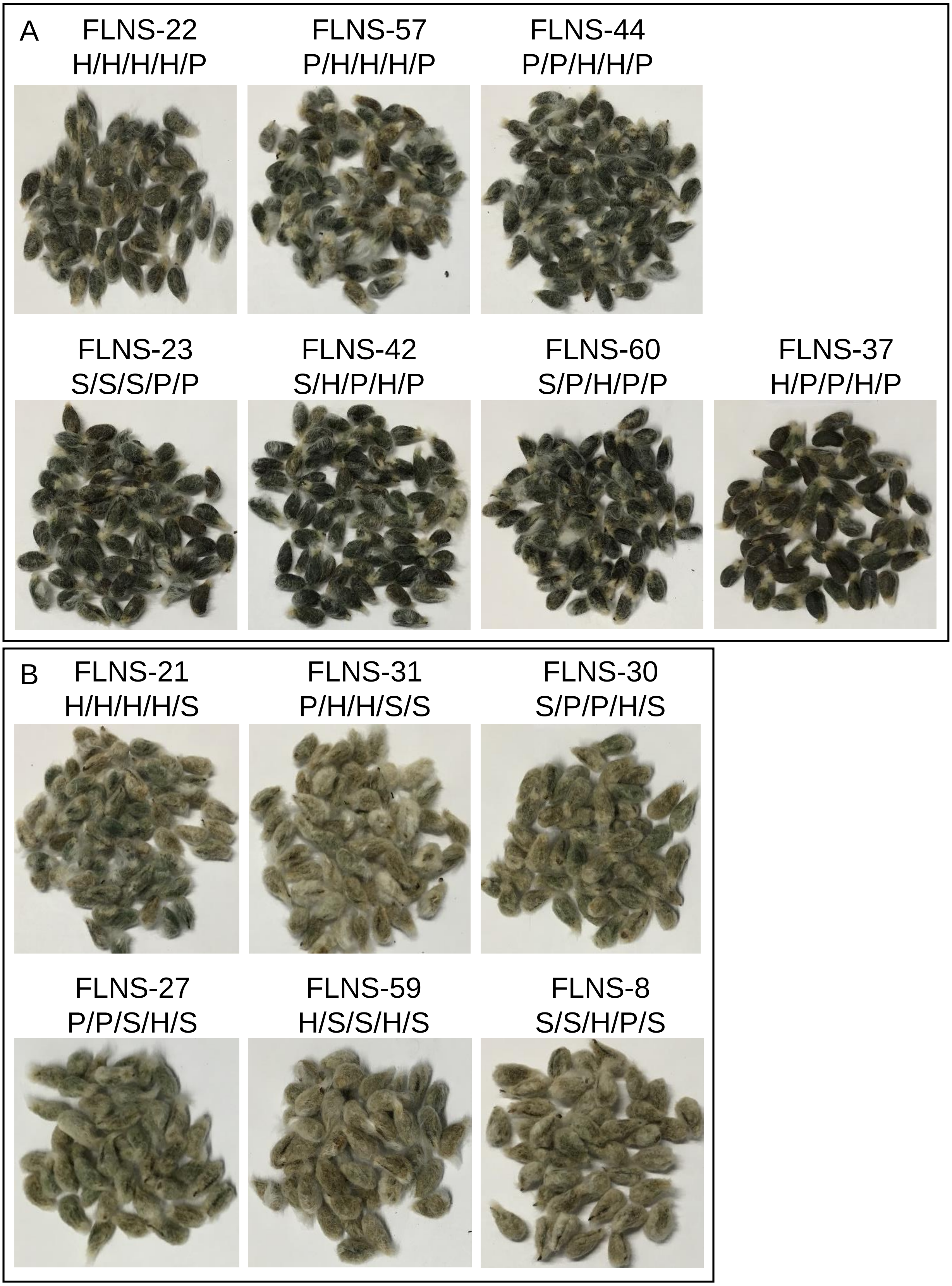

Figure S5 Representative fuzz phenotype of the FLNS F_2:F_3 families with a genotype of homozygous Pima S-7 (A) or Sicala 40 (B) for Locus V. F_2:F_3 family homozygous for Pima S-7 had less fuzz than F_2:F_3 family homozygous for Sicala 40. The genotypes of loci I/II/III/IV are also shown for each F_2:F_3 family. S: homozygous Sicala 40; P: homozygous Pima S-7; H: heterozygous.

M210235	M210399	M210445	M210676	M210353
0.60%	3.02%	3.99%	7.04%	9.20%

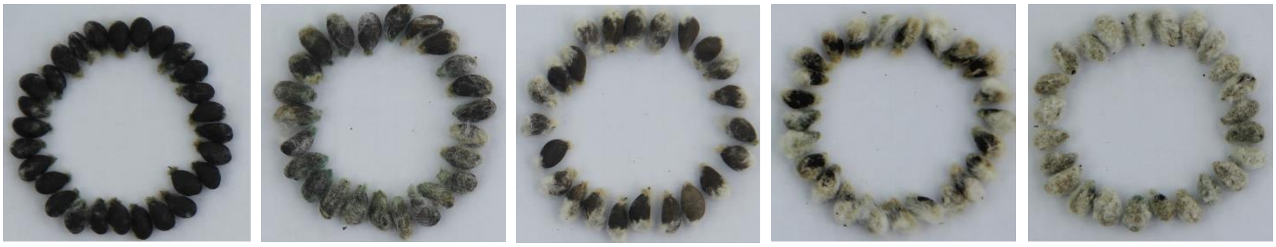

Figure S6 Seed fuzz phenotype of representative *G. barbadense* accessions.

```
Xu142fl_At     MQQSPCSDKVGLKKGPWTPEEDQKLLSYIQEHGGGSWRGLPAKAGLQRCGKSCRLRWINY 60
Xu142_At       MQQSPCSDKVGLKKGPWTPEEDQKLLSYIQEHGGGSWRGLPAKAGLQRCGKSCRLRWINY 60
TM-1_At        MQQSPCSDKVGLKKGPWTPEEDQKLLSYIQEHGGGSWRGLPAKAGLQRCGKSCRLRWINY 60
Pima S-7_At    MQQSPCSDKVGLKKGPWTPEEDQKLSSYIQEHGGGSWRGLPAKAGLQRCGKSCRLRWINY 60
Xu142fl_Dt     MQQSPCSDKVGLKKGPWTPEEDQKLLSYIQEHGGGSWRGLPAKAGLQRCGKSCRLRWINY 60
Xu142_Dt       MQQSPCSDKVGLKKGPWTPEEDQKLLSYIQEHGGGSWRGLPAKAGLQRCGKSCRLRWINY 60
TM-1_Dt        MQQSPCSDKVVLKKGPWTPEEDQKLLSYIQEHGGGSWRGLPAKAGLQRCGKSCRLRWINY 60
Pima S-7_Dt    MQQSPCSDKVVLKKGPWTPEEDQKLLSYIQEHGGGSWRGLPAKAGLQRCGKSCRLRWINY 60
               ********** ************** **********************************

Xu142fl_At     LRPDIKRGKFSSQEERTIIQLHALLGNRWSAIAAHLPKRTDNEIMNYWNTQLKKRLTTIG 120
Xu142_At       LRPDIKRGKFSSQEERTIIQLHALLGNRWSAIAAHLPKRTDNEIKNYWNTQLKKRLTTIG 120
TM-1_At        LRPDIKRGKFSSQEERTIIQLHALLGNRWSAIAAHLPKRTDNEIKNYWNTQLKKRLTTIG 120
Pima S-7_At    LRPDIKRGKFSSQEERTIIQLHALLGNRWSAIAAHLPKRTDNEIKNYWNTQLKKRLTTIG 120
Xu142fl_Dt     LRPDIKRGKFSSQEERTIIQLHALLGNRWSAIAAHLPKRTDNEIKNYWNTQLKKRLTKIG 120
Xu142_Dt       LRPDIKRGKFSSQEERTIIQLHALLGNRWSAIAAHLPKRTDNEIKNYWNTQLKKRLTKIG 120
TM-1_Dt        LRPDIKRGKFSSQEERTIIQLHALLGNRWSAIAAHLPKRTDNEIKNYWNTQLKKRLTKIG 120
Pima S-7_Dt    LRPDIKRGKFSSQEERTIIQLHALLGNRWSAIAAHLPKRTDNEIKNYWNTQLKKRLTKIG 120
               ******************************************** ************.**

Xu142fl_At     IDPATHRPKTDTLGSTPKDAANLSHMAQWESARLEAEARLVRESKRVSNPSQNQFRFTSS 180
Xu142_At       IDPATHRPKTDTLGSTPKDAANLSHMAQWESARLEAEARLVRESKRVSNPSQNQFRFTSS 180
TM-1_At        IDPATHRPKTDTLGSTPKDAANLSHMAQWESARLEAEARLVRESKRVSNPPQNQFRFTSS 180
Pima S-7_At    IDPATHRPKTDTLGSTPKDAANLSHMAQWESARLEAEARLVRESKRVSNPPQNQFRFTSS 180
Xu142fl_Dt     IDPATHRPKTDTLGSTPKDVANFSHMAQWESARLEAEARLVRESKRVSNPPQNQFRFTSS 180
Xu142_Dt       IDPATHRPKTDTLGSTPKDVANFSHMAQWESARLEAEARLVRESKRVSNPPQNQFRFTSS 180
TM-1_Dt        IDPATHRPKTDTLGSTPKDAANLSHMAQWESARLEAEARLVRESKRVSNPPQNQFRFTSS 180
Pima S-7_Dt    IDPATHRPKTDTLGSTPKDVANLSHMAQWESARLEAEARLVRESKRVSNPPQNQFRFTSS 180
               *******************.**:*************************** *********

Xu142fl_At     SAPPLVSKIDVGLAHATKPQCLDVLKAWQRVVTGLFTFNTDNLQSPTSTSSFTENTLPIS 240
Xu142_At       SAPPLVSKIDVGLAHATKPQCLDVLKAWQRVVTGLFTFNTDNLQSPTSTSSFTENTLPIS 240
TM-1_At        SAPPLVNKIDVGLAHATKPQCLDVLKAWQRVVTGLFTFNTDNLQSPTSTSSFTENTLPIS 240
Pima S-7_At    SAPPLVSKIDVGLAHATKPQCLDVLKAWQRVVTGLFTFNTDNLQSPTSTSSFTENTLPIS 240
Xu142fl_Dt     SAPPLVNKIDVGLAHATKPQCLDVLKAWQRVVTGLFTFNTDNLQSPTSTSSFTENRLPIS 240
Xu142_Dt       SAPPLVNKIDVGLAHATKPQCLDVLKAWQRVVTGLFTFNTDNLQSPTSTSSFTENRLPIS 240
TM-1_Dt        SAPPLVNKIDVGLAHATKPQCLDVLKAWQRVVTGLFTFNTDNLQSPTSTSSFTENRLPIS 240
Pima S-7_Dt    SAPPLVNKIDVGLAHATKPQCLDVLKAWQRVVTGLFTFNTDNLQSPTSTSSFTENRLPIS 240
               ******.************************************************ ****

Xu142fl_At     SVGFIDSFVGNSNNSCCGNNWECVEKSSQVAELQERLDNSMGLHDILDLSSEDVWFQGSY 300
Xu142_At       SVGFIDSFVGNSNNSCCGNNWECVEKSSQVAELQERLDNSMGLHDILDLSSEDVWFQGSY 300
TM-1_At        SVGFIDSFVGNSNNSCCGNNWECVEKSSQVAELQEILDNSMGLHDILDLSSEDVWFQGSY 300
Pima S-7_At    SVGFIDSFVGNSNNSCCGNNWECVEKSSQVAELQERLDNSMGLHDILDLSSEDVWFQGSY 300
Xu142fl_Dt     SVGFIDSFVGNSNNSCCGNNWECVEKSSQVAELQERLDNSMGLHDILDFSSEDVWFQGSY 300
Xu142_Dt       SVGFIDSFVGNSNNSCCGNNWECVEKSSQVAELQERLDNSMGLHDILDFSSEDVWFQGSY 300
TM-1_Dt        SVGFIDSFVGNSNNSCCGNNWECVEKSSQVAELQERLDNSMGLHDILDFSSEDVWFQGSY 300
Pima S-7_Dt    SVGFIDSFVGNSNNSCCGNNWECVEKSSQVAELQERLDNSMGLHDILDFSSEDVWFQGSY 300
               *********************************** ************:***********

Xu142fl_At     RAENMMEGYSDTLMVCDSGDHPKSLSMEPRQNFNVGTSNASSFEENKNYWNNILNFANAS 360
Xu142_At       RAENMMEGYSDTLMVCDSGDHPKSLSMEPRQNFNVGTSNASSFEENKNYWNNILNFANAS 360
TM-1_At        RAENMMEGYSDTLMVCDSGDHPKSLSMEPRQNFNVGTSNASSFEENKNYWNNILNFANAS 360
Pima S-7_At    RAENMMEGYSDTLMVCDSGDHPKSLSMEPRQNFNVGTSNASSFEENKNYWNNILNFANAS 360
Xu142fl_Dt     RAENMMEGYSDTLMVCDSGDHQKSLSMEPRQNFNVGTSNASSFEENKNYWNNILNFANAS 360
Xu142_Dt       RAENMMEGYSDTLMVCDSGDHQKSLSMEPRQNFNVGTSNASSFEENKNYWNNILNFANAS 360
TM-1_Dt        RAENMMEGYSDTLMVCDSGDHPKSLSMEPRQNFNVGTSNASSFEENKNYWNNILNFANAS 360
Pima S-7_Dt    RAENMMEGYSDTLMVCDSGDHQKSLSMEPRQNFNVGTSNASSFEENKNYWNNILNFANAS 360
               ********************* **************************************

Xu142fl_At     PSGSSVF   367
Xu142_At       PSGSSVF   367
TM-1_At        PSGSSVF   367
Pima S-7_At    PSGSSVF   367
Xu142fl_Dt     PSGSSVF   367
Xu142_Dt       PSGSSVF   367
TM-1_Dt        PSGSSVF   367
Pima S-7_Dt    PSGSSVF   367
               *******
```

Figure S7 Alignment of the amino acid sequences of *MYB25-like* from Xu142, Xu142*fl*, TM-1 and Pima S-7. The conserved MYB-binding and SANT domains are highlighted in yellow and green, respectively. The amino acid that is different between Xu142_At and Xu142*fl*_At in the SANT domain is shown in red. The two amino acids that are different between TM-1_Dt and Pima S-7_Dt are highlighted in red.

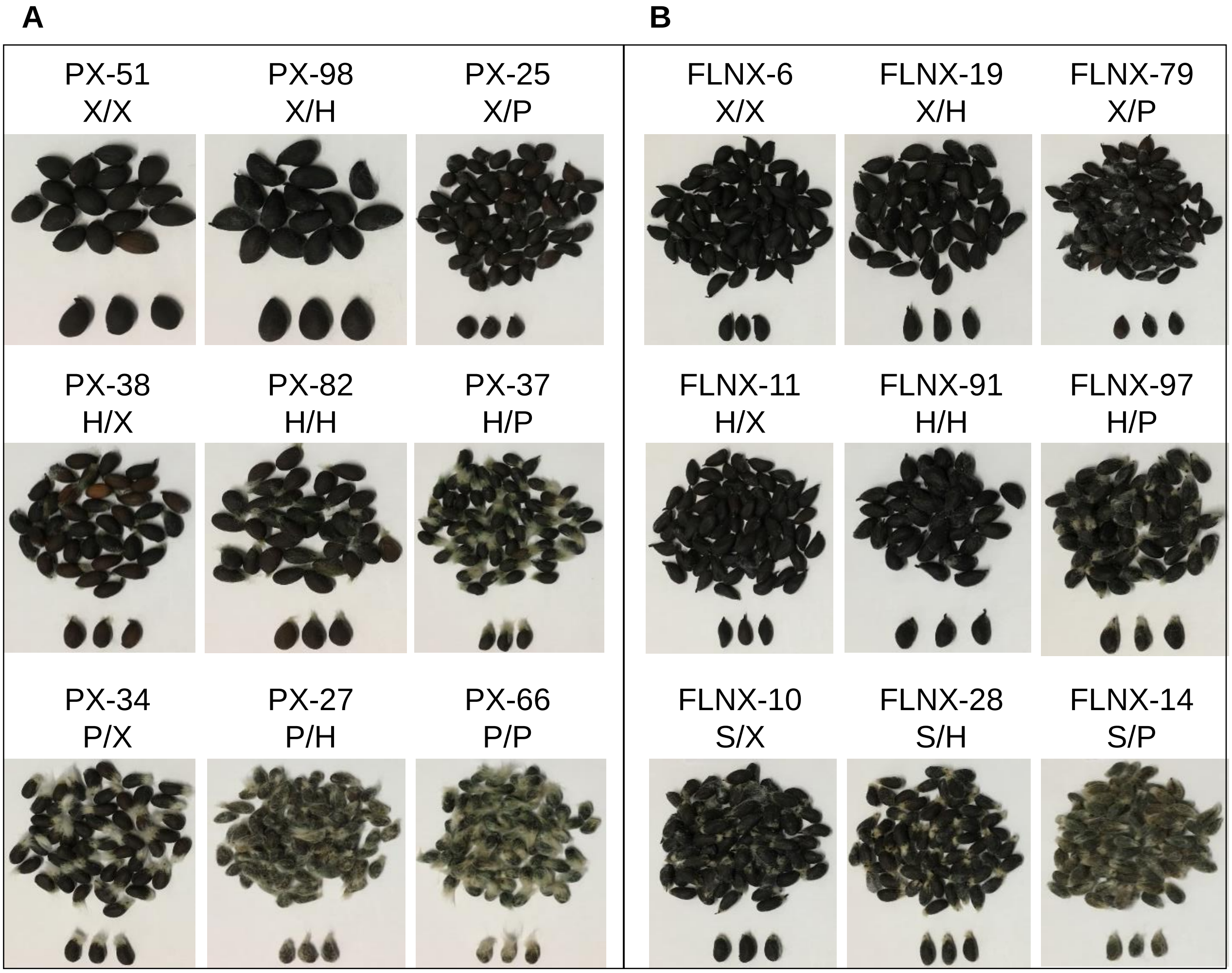

Figure S9 Distribution of fuzz phenotypes (F_3 seeds) in the F_2 populations PX and FLNX by *MYB25-like* homoeoalleles. (A) Representative fuzz phenotypes of the PX F_2 population derived from Pima S-7 x Xu142*fl*. (B) Representative fuzz phenotypes of the FLNX F_2 population derived from FLN1-10 x Xu142*fl*. The genotype of *MYB25-like_At/MYB25-like_Dt* of each F_2 plant are shown blow the plant ID. X: homozygous Xu142*fl*; P: homozygous Pima S-7; S: homozygous Sicala 40; H: heterozygous.

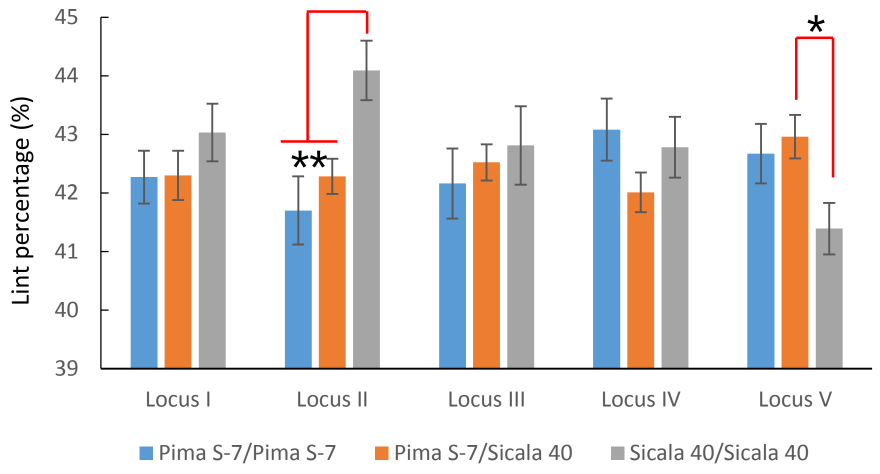

Figure S10 Effects of fuzz associated loci on lint percentage of the FLNS F_2:F_3 families. F_2 plants were genotyped using the markers shown in Fig. 3A. For each locus, F_2 plants were grouped based on their genotypes, *i.e.* homozygous Pima S-7 (Pima S-7/Pima S-7) or Sicala 40 (Sicala 40/Sicala 40), or heterozygous (Pima S-7/Sicala 40) individuals. Error bars represent standard errors. * and ** denote a significant difference at $p<0.05$ and $p<0.01$, respectively, determined by a Student's *t*-test.
