# Supplementary material for: Genetic dissection of the fuzzless seed trait in Gossypium barbadense
Source: J Exp Bot. 2018 Jan 17;69(5):997–1009. doi: 10.1093/jxb/erx459 (PMC6018843; doi:10.1093/jxb/erx459)
Supplement: Supplementary_Tables_S1-S3 [file erx459_suppl_supplementary_tables_s1-s3.pdf]

Supplementary Table S1 Primers used in this study

| Primer ID | Primer sequence (5' - 3')* | Usage |
|---|---|---|
| Myb25like_A1 (D12_47603353A1) | CAGAAGAAGACCAAAAACTCC | KASP, distinguish MYB25-like_Dt of Xu142fl and Pima S-7, Region11 (Locus V) |
| Myb25like_A2 (D12_47603353A2) | CAGAAGAAGACCAAAAACTCT | KASP, distinguish MYB25-like_Dt of Xu142fl and Pima S-7, Region11 (Locus V) |
| Myb25like_C (D12_47603353C) | CTAAGAAGAGATGTGGAAATAATGACC | KASP, distinguish MYB25-like_Dt of Xu142fl and Pima S-7, Region11 (Locus V) |
| Xu142flAt_A1 | GAACAGACAATGAGATCAA | KASP, distinguish MYB25-like_At of Xu142 and others |
| Xu142flAt_A2 | GAACAGACAATGAGATCAT | KASP, distinguish MYB25-like_At of Xu142 and others |
| Xu142flAt_C | GAGGGTATCGGTTTTAGGC | KASP, distinguish MYB25-like_At of Xu142 and others |
| A12g1503_A1 | GAAGAAGACCAAAAACTCTT | KASP, distinguish MYB25-like_At of Pima S-7 and Sicala 40 |
| A12g1503_A2 | GAAGAAGACCAAAAACTCTC | KASP, distinguish MYB25-like_At of Pima S-7 and Sicala 40 |
| A12g1503_C | GATGTGGAAATAATACCAGC | KASP, distinguish MYB25-like_At of Pima S-7 and Sicala 40 |
| A03_97995070A1 | GCACTACCAGAATACAAATG | KASP, Region1 |
| A03_97995070A2 | GCACTACCAGAATACAAATA | KASP, Region1 |
| A03_97995070C | CAGGATTGATGAACATCGGG | KASP, Region1 |
| A08_1022550A1 | TGCAGGCATCCACCAATA | KASP, Region2 (Locus I) |
| A08_1022550A2 | TGCAGGCATCCACCAATT | KASP, Region2 (Locus I) |
| A08_1022550C | AAACTCCAATTCCTAACCTACC | KASP, Region2 (Locus I) |
| A08_1030181A1 | TCTCAAGTACAGTCAATCGA | KASP, Region2 (Locus I) |
| A08_1030181A2 | TCTCAAGTACAGTCAATCGG | KASP, Region2 (Locus I) |
| A08_1030181C | TTTAATCAATCGGTTTGGTCG | KASP, Region2 (Locus I) |
| A10_6287475A1 | TTCGTCCTAAGAAACACGCT | KASP, Region3 |
| A10_6287475A2 | TTCGTCCTAAGAAACACGCC | KASP, Region3 |
| A10_6287475C | GCTATTGACGAGTCATCCAAG | KASP, Region3 |
| A10_8981057A1 | AGGTCCTTGTATCTCTG | KASP, Region3 |
| A10_8981057A2 | AGGTCCTTGTATCTCTC | KASP, Region3 |
| A10_8981057C | GGTATCTCTTCTTTGGTACAG | KASP, Region3 |
| A10_20181494A1 | TAGGTCCAGTAATGCTCCA | KASP, Region4 (Locus II) |
| A10_20181494A2 | TAGGTCCAGTAATGCTCCG | KASP, Region4 (Locus II) |
| A10_20181494C | GCTATTACAATTTGTTCCTACC | KASP, Region4 (Locus II) |
| A10_57848894A1 | CGACAGATTTTGACCTAGAG | KASP, Region5 (Locus III) |
| A10_57848894A2 | CGACAGATTTTGACCTAGAC | KASP, Region5 (Locus III) |
| A10_57848894C | CAAGGCATCGTAGAAGAAGGTC | KASP, Region5 (Locus III) |
| A10_58277903A1 | AGGTCTTTCAACTAGGCTT | KASP, Region5 (Locus III) |
| A10_58277903A2 | AGGTCTTTCAACTAGGCTC | KASP, Region5 (Locus III) |
| A10_58277903C | GCCTACAAGTATCAAAGAAGCAC | KASP, Region5 (Locus III) |
| A10_58706671A1 | TGCCACTTATCCCATGTCCG | KASP, Region5 (Locus III) |
| A10_58706671A2 | TGCCACTTATCCCATGTCCA | KASP, Region5 (Locus III) |
| A10_58706671C | CCAAACCCAATCAAGTATCCC | KASP, Region5 (Locus III) |

| | | |
|---|---|---|
| A11_13920555A1 | TATTCAGACCAGGCTTGGGT | KASP, Region6 |
| A11_13920555A2 | TATTCAGACCAGGCTTGGGC | KASP, Region6 |
| A11_13920555C | GATTGTGTGTCAGGTTCAGG | KASP, Region6 |
| A11_15966494A1 | TTCCGCTATCCAAGAACCGC | KASP, Region6 |
| A11_15966494A2 | TTCCGCTATCCAAGAACCGT | KASP, Region6 |
| A11_15966494C | GTTGTAGTTGCACCTGATTCG | KASP, Region6 |
| D05G0978_823543A1 | CGTCATGCAAAGATGAGAGAT | KASP, Region8 |
| D05G0978_8235432A2 | CGTCATGCAAAGATGAGAGAG | KASP, Region8 |
| D05G0978_8235432C | CATCAATTAAAAACCTCTCCCACC | KASP, Region8 |
| D07_12347328A1 | TATGGTGCTTGTGTTTGACG | KASP, Region9 (Locus IV) |
| D07_12347328A2 | TATGGTGCTTGTGTTTGACA | KASP, Region9 (Locus IV) |
| D07_12347328C | GTTTTCTTACACTATATTGTGG | KASP, Region9 (Locus IV) |
| D07_13012711A1 | GGATTCGGATACCCTACTCG | KASP, Region9 (Locus IV) |
| D07_13012711A2 | GGATTCGGATACCCTACTCC | KASP, Region9 (Locus IV) |
| D07_13012711C | GGTCCTCCTTTGAATTTCCA | KASP, Region9 (Locus IV) |
| D11_4264641A1 | ACATATTTAGATCTGGCGAC | KASP, Region10 |
| D11_4264641A2 | ACATATTTAGATCTGGCGAT | KASP, Region10 |
| D11_4264641C | CTACAATGTCAAAATCCCTAC | KASP, Region10 |
| D11_4446303A1 | GTGCAATTCAGATGTGACAC | KASP, Region10 |
| D11_4446303A1 | GTGCAATTCAGATGTGACAT | KASP, Region10 |
| D11_4446303A1 | CACCGACTGTTCAAGTTAAGC | KASP, Region10 |
| A12_74507074A1 | CCCCATTGAGTTATCCAATA | KASP, non-association control |
| A12_74507074A2 | CCCCATTGAGTTATCCAATC | KASP, non-association control |
| A12_74507074C | CATTGACAGCTTTGTGGGGA | KASP, non-association control |
| MYB25L_F | GATCGACCCTGCAACTCACA | qPCR, both At and Dt of MYB25-like |
| 08g179600DtF5C | CACAGTTGAAGAAAAGATTGACCCA | qPCR, MYB25-like_Dt |
| 08g179600_R2 | GGTTAGCGGCATCCTTGGGAG | qPCR, both At and Dt of MYB25-like |
| Ubi_F | CCAGAAGGAATCCACTTTGC | qPCR, Ubiquitin |
| Ubi_R | CCAGCTCACATCAGCATACG | qPCR, Ubiquitin |

* For the primers used in KASP, the 5' extensions of the A1 and A2 were not shown due to proprietary.

Supplementary Table S2 Chromosomal distribution of 5426 polymorphic SNPs between Pima S-7 and Sicala 40 based on the Cotton SNP63K array

| Chromosome | No. of SNPs | Chromosome | No. of SNPs |
|---|---|---|---|
| A01 | 228 | D01 | 79 |
| A02 | 5 | D02 | 90 |
| A03 | 110 | D03 | 87 |
| A04 | 5 | D04 | 16 |
| A05 | 93 | D05 | 104 |
| A06 | 104 | D06 | 214 |
| A07 | 210 | D07 | 271 |
| A08 | 511 | D08 | 513 |
| A09 | 211 | D09 | 184 |
| A10 | 324 | D10 | 230 |
| A11 | 247 | D11 | 233 |
| A12 | 155 | D12 | 267 |
| A13 | 45 | D13 | 144 |
| Scaffold | 746 | | |

Supplementary Table S3 Candidate regions identified based on mapping-by-sequencing (MBS) and SNP array

| Candidate region | Chromosome | Coordinates (start::end) | | Region associated with fuzz %? | Overlapping interval between MBS and SNP array | Size of the interval (bp) | No. of genes in the interval |
|---|---|---|---|---|---|---|---|
| | | MBS | SNP array# | | | | |
| Region1 | A03 | 97002205::98339209 | ni | No | | | |
| Region2 | A08 (locus I) | 581980::6070679 | ni (606264::4824766) | Yes | 606264::4824766 | 4218502 | 314 |
| Region3 | A10-1 | 6197847::10760000 | ni | No | | | |
| Region4 | A10-2 (locus II) | 17240145::21463786 | ni (18771617::19945462) | Yes | 18771617::19945462 | 1173845 | 34 |
| Region5 | A10-3 (locus III) | 56851983::73544008 | ni (57150183::58365745) | Yes | 57150183::58365745 | 1215562 | 22 |
| Region6 | A11 | 12753735::18040622 | ni | No | | | |
| Region7 | A12 | ni | 75882859::77545244* | No | | | |
| Region8 | D05 | ni | 8215296::8237410^ | No | | | |
| Region9 | D07 (locus IV) | 10569829::13973680 | ni (11000345::19225750) | Yes | 11054420::13973680 | 2919260 | 169 |
| Region10 | D11 | 4247822::4506814 | ni | No | | | |
| Region11 | D12 (locus V) | 42953348::47758100 | 46359219::48033804 | Yes | 46359219::47758100 | 1398881 | 84 |

ni: not identified

#: the interval was determined based on the individuals showing co-segregatiion of phenotype and genotype

*: only 3 markers in the interval and all were mapped to Chr26 (D12) based on Hulse-Kemp et al. (2015b)

^: 6 markers in the interval
