# Supplementary material for: Genetic dissection of the fuzzless seed trait in Gossypium barbadense
Source: J Exp Bot. 2018 Jan 17;69(5):997–1009. doi: 10.1093/jxb/erx459 (PMC6018843; doi:10.1093/jxb/erx459)
Supplement: Supplementary_Tables_S4 [file erx459_suppl_supplementary_tables_s4.pdf]

Supplementary Table S4 List of genes in the chromosomal regions associated with fuzz development

Gene ID	Coordinate1	Coordinate2	Annotation
Locus I			
Gh_A08G0068	605735	606576	Polynucleotidyl transferase; ribonuclease H-like superfamily protein
Gh_A08G0069	607704	618632	BRI1 suppressor 1 (BSU1)-like 3
Gh_A08G0070	626811	632509	Tetratricopeptide repeat (TPR)-like superfamily protein
Gh_A08G0071	635236	638304	RING-H2 group F2A
Gh_A08G0072	639890	642641	
Gh_A08G0073	645188	647657	HSP20-like chaperones superfamily protein
Gh_A08G0074	651859	655829	Calcium-binding EF hand family protein
Gh_A08G0075	660080	664049	RING/U-box superfamily protein
Gh_A08G0076	666649	667029	
Gh_A08G0077	670360	676471	nitrate excretion transporter1
Gh_A08G0078	677321	680640	Transducin/WD40 repeat-like superfamily protein
Gh_A08G0079	682587	690807	vacuolar proton ATPase A1
Gh_A08G0080	715646	716431	Dof-type zinc finger DNA-binding family protein
Gh_A08G0081	729373	731507	LOB domain-containing protein 11
Gh_A08G0082	738593	739852	proton gradient regulation 3
Gh_A08G0083	741880	742266	
Gh_A08G0084	747155	747481	Preprotein translocase Sec; Sec61-beta subunit protein
Gh_A08G0085	756097	760011	geminivirus rep interacting kinase 2
Gh_A08G0086	761897	763855	chaperonin 10
Gh_A08G0087	766191	768544	cofactor of nitrate reductase and xanthine dehydrogenase 2
Gh_A08G0088	770637	772278	Ribosomal protein L30/L7 family protein
Gh_A08G0089	774792	775352	molybdopterin biosynthesis MoaE family protein
Gh_A08G0090	778549	783180	transferases;nucleotidyltransferases
Gh_A08G0091	789400	790349	Copper transport protein family
Gh_A08G0092	792104	797877	dgd1 suppressor 1
Gh_A08G0093	799701	800255	ribosomal protein L12-A
Gh_A08G0094	802103	802803	poly(A) binding protein 3
Gh_A08G0095	807246	808190	C2H2 type zinc finger transcription factor family
Gh_A08G0096	814685	816085	cytochrome P450; family 94; subfamily D; polypeptide 2
Gh_A08G0097	820571	821569	Pentatricopeptide repeat (PPR) superfamily protein

Gh_A08G0098	824253	825135	Pentatricopeptide repeat (PPR) superfamily protein
Gh_A08G0099	828676	829659	BREVIS RADIX-like 4
Gh_A08G0100	831622	836352	Regulator of chromosome condensation (RCC1) family with FYVE zinc finger domain
Gh_A08G0101	861724	863597	TBP-associated factor 7
Gh_A08G0102	866185	868446	Lipase/lipooxygenase; PLAT/LH2 family protein
Gh_A08G0103	868941	873648	protein phosphatase 2A-4
Gh_A08G0104	882017	884195	Bifunctional inhibitor/lipid-transfer protein/seed storage 2S albumin superfamily protein
Gh_A08G0105	887814	892025	RNA-binding KH domain-containing protein
Gh_A08G0106	897796	898770	Family of unknown function (DUF716)
Gh_A08G0107	913522	914115	Disease resistance-responsive (dirigent-like protein) family protein
Gh_A08G0108	921934	922311	
Gh_A08G0109	924211	924516	
Gh_A08G0110	925654	939192	Protein kinase family protein
Gh_A08G0111	942970	981899	glucan synthase-like 10
Gh_A08G0112	987489	988481	AP2/B3 transcription factor family protein
Gh_A08G0113	993164	996534	NAD(P)-binding Rossmann-fold superfamily protein
Gh_A08G0114	998459	1002012	Emsy N Terminus (ENT)/ plant Tudor-like domains-containing protein
Gh_A08G0115	1006795	1011386	
Gh_A08G0116	1013789	1015027	AGC (cAMP-dependent; cGMP-dependent and protein kinase C) kinase family protein
Gh_A08G0117	1031008	1047041	NIMA-related kinase 5
Gh_A08G0118	1048984	1055274	outer envelope protein of 80 kDa
Gh_A08G0119	1057094	1060321	protein serine/threonine kinases;ATP binding;catalytics
Gh_A08G0120	1078005	1082197	RING/U-box superfamily protein
Gh_A08G0121	1084526	1097630	lysophosphatidyl acyltransferase 2
Gh_A08G0122	1107626	1111538	RNA-binding (RRM/RBD/RNP motifs) family protein
Gh_A08G0123	1112762	1119644	origin of replication complex 1B
Gh_A08G0124	1132198	1136371	CRM family member 3A
Gh_A08G0125	1136804	1139061	ribosome biogenesis regulatory protein (RRS1) family protein
Gh_A08G0126	1143485	1146384	evolutionarily conserved C-terminal region 2
Gh_A08G0127	1148892	1155704	Lysyl-tRNA synthetase; class II
Gh_A08G0128	1169139	1173759	cytochrome P450; family 716; subfamily A; polypeptide 1
Gh_A08G0129	1184451	1184681	
Gh_A08G0130	1188184	1189767	Tetratricopeptide repeat (TPR)-like superfamily protein

Gh_A08G0131	1194031	1195443	F-box and associated interaction domains-containing protein
Gh_A08G0132	1198692	1199801	F-box and associated interaction domains-containing protein
Gh_A08G0133	1206673	1207791	F-box and associated interaction domains-containing protein
Gh_A08G0134	1220398	1221810	F-box and associated interaction domains-containing protein
Gh_A08G0135	1226599	1227708	F-box and associated interaction domains-containing protein
Gh_A08G0136	1258378	1259471	F-box and associated interaction domains-containing protein
Gh_A08G0137	1276309	1277460	F-box and associated interaction domains-containing protein
Gh_A08G0138	1280549	1285631	Protein kinase superfamily protein
Gh_A08G0139	1289685	1294363	ARM repeat superfamily protein
Gh_A08G0140	1294754	1295488	
Gh_A08G0141	1320838	1322935	alpha/beta-Hydrolases superfamily protein
Gh_A08G0142	1328225	1329880	alpha/beta-Hydrolases superfamily protein
Gh_A08G0143	1342590	1348723	cycloartenol synthase 1
Gh_A08G0144	1355393	1359375	protein serine/threonine kinases;protein kinases;ATP binding;sugar binding;kinases;carbohydrate binding
Gh_A08G0145	1360470	1367897	transducin family protein / WD-40 repeat family protein
Gh_A08G0146	1370593	1425719	Transducin/WD40 repeat-like superfamily protein
Gh_A08G0147	1425790	1426257	
Gh_A08G0148	1430786	1431430	
Gh_A08G0149	1432533	1435881	RNA-binding (RRM/RBD/RNP motifs) family protein
Gh_A08G0150	1436829	1440964	myb-like HTH transcriptional regulator family protein
Gh_A08G0151	1444635	1447087	chaperone binding;ATPase activators
Gh_A08G0152	1449010	1464668	
Gh_A08G0153	1466339	1467817	Ubiquitin carboxyl-terminal hydrolase family protein
Gh_A08G0154	1469328	1471841	S-locus lectin protein kinase family protein
Gh_A08G0155	1472956	1477228	regulatory particle non-ATPase 13
Gh_A08G0156	1483462	1485591	Leucine-rich repeat protein kinase family protein
Gh_A08G0157	1491172	1492005	Leucine-rich repeat protein kinase family protein
Gh_A08G0158	1501016	1513459	autoinhibited Ca(2+)-ATPase 10
Gh_A08G0159	1529900	1534947	TRICHOME BIREFRINGENCE-LIKE 5
Gh_A08G0160	1543084	1546994	RNA-binding KH domain-containing protein
Gh_A08G0161	1576215	1583323	subtilisin-like serine protease 3
Gh_A08G0162	1585175	1587157	rna processing factor 2
Gh_A08G0163	1590314	1593141	Core-2/I-branching beta-1;6-N-acetylglucosaminyltransferase family protein

Gh_A08G0164	1602745	1607164	metaxin-related
Gh_A08G0165	1609729	1617201	valyl-tRNA synthetase / valine--tRNA ligase (VALRS)
Gh_A08G0166	1619387	1622482	S-adenosyl-L-methionine-dependent methyltransferases superfamily protein
Gh_A08G0167	1623900	1627469	S-adenosyl-L-methionine-dependent methyltransferases superfamily protein
Gh_A08G0168	1661189	1666868	PDI-like 5-3
Gh_A08G0169	1669026	1673900	Ypt/Rab-GAP domain of gyp1p superfamily protein
Gh_A08G0170	1674619	1678643	formin homology5
Gh_A08G0171	1716135	1719173	Leucine-rich repeat transmembrane protein kinase family protein
Gh_A08G0172	1728675	1729610	histone H2A 10
Gh_A08G0173	1737158	1739425	plastid developmental protein DAG; putative
Gh_A08G0174	1744554	1744856	Calcium-binding EF-hand family protein
Gh_A08G0175	1745923	1752129	ubiquitin-specific protease 14
Gh_A08G0176	1754928	1756864	Protein kinase superfamily protein
Gh_A08G0177	1756867	1758150	Protein kinase superfamily protein
Gh_A08G0178	1761442	1763104	MYB-like 102
Gh_A08G0179	1776126	1778325	Protein of unknown function (DUF1223)
Gh_A08G0180	1784388	1785546	Toll-Interleukin-Resistance (TIR) domain family protein
Gh_A08G0181	1811707	1816976	Seven transmembrane MLO family protein
Gh_A08G0182	1819020	1827222	Protein kinase superfamily protein
Gh_A08G0183	1828990	1833383	embryo defective 2737
Gh_A08G0184	1836691	1845060	Auxin-responsive GH3 family protein
Gh_A08G0185	1851405	1858713	P-loop containing nucleoside triphosphate hydrolases superfamily protein
Gh_A08G0186	1860419	1861756	
Gh_A08G0187	1862847	1864995	
Gh_A08G0188	1884282	1884638	
Gh_A08G0189	1887614	1887967	
Gh_A08G0190	1904548	1904907	
Gh_A08G0191	1924683	1927435	Lactate/malate dehydrogenase family protein
Gh_A08G0192	1929796	1930828	CONSTANS-like 5
Gh_A08G0193	1938799	2007157	Deoxyxylulose-5-phosphate synthase
Gh_A08G0194	2030430	2035856	Calmodulin-binding protein
Gh_A08G0195	2040072	2041080	xyloglucan endotransglycosylase 6
Gh_A08G0196	2045365	2046186	nicotianamine synthase 4

Gh_A08G0197	2072085	2072570	zinc finger protein 2
Gh_A08G0198	2104606	2106438	Syntaxin/t-SNARE family protein
Gh_A08G0199	2107006	2108392	
Gh_A08G0200	2111278	2112006	
Gh_A08G0201	2114464	2119237	P450 reductase 2
Gh_A08G0202	2122477	2127105	Dihydrolipoamide succinyltransferase
Gh_A08G0203	2130609	2134059	aspartic proteinase A1
Gh_A08G0204	2142260	2143877	glutathione S-transferase TAU 16
Gh_A08G0205	2147605	2149852	RNAse THREE-like protein 3
Gh_A08G0206	2153108	2153785	ethylene responsive element binding factor 3
Gh_A08G0207	2155456	2158483	cytidinediphosphate diacylglycerol synthase 2
Gh_A08G0208	2161399	2165413	RING/FYVE/PHD zinc finger superfamily protein
Gh_A08G0209	2167957	2170585	ATPase; V0 complex; subunit E
Gh_A08G0210	2171660	2173803	syntaxin of plants 51
Gh_A08G0211	2177090	2178590	Ribosomal protein S8e family protein
Gh_A08G0212	2183293	2186523	WRKY family transcription factor family protein
Gh_A08G0213	2190471	2194290	Haloacid dehalogenase-like hydrolase (HAD) superfamily protein
Gh_A08G0214	2201257	2201978	
Gh_A08G0215	2216058	2221163	beta-amylase 4
Gh_A08G0216	2254518	2256860	Histone superfamily protein
Gh_A08G0217	2258295	2260591	nicotinate/nicotinamide mononucleotide adenyltransferase
Gh_A08G0218	2273290	2275943	ABC-2 type transporter family protein
Gh_A08G0219	2276489	2279126	heat shock protein 81-3
Gh_A08G0220	2308805	2311733	heat shock protein 81-3
Gh_A08G0221	2316646	2319598	Subtilase family protein
Gh_A08G0222	2321670	2327542	Transducin/WD40 repeat-like superfamily protein
Gh_A08G0223	2330202	2331843	ubiquitin-conjugating enzyme 30
Gh_A08G0224	2333535	2334113	prenylated RAB acceptor 1.G2
Gh_A08G0225	2337102	2341462	
Gh_A08G0226	2420669	2423827	proline-rich family protein
Gh_A08G0227	2426790	2439604	protein N-terminal asparagine amidohydrolase family protein
Gh_A08G0228	2439937	2440263	
Gh_A08G0229	2446800	2449813	2-oxoglutarate (2OG) and Fe(II)-dependent oxygenase superfamily protein

Gh_A08G0230	2450729	2452961	basic helix-loop-helix (bHLH) DNA-binding superfamily protein
Gh_A08G0231	2461248	2464306	SWAP (Suppressor-of-White-APricot)/surp domain-containing protein / D111/G-patch domain-containing protein
Gh_A08G0232	2475980	2495610	Leucine-rich repeat receptor-like protein kinase family protein
Gh_A08G0233	2497768	2498289	A20/AN1-like zinc finger family protein
Gh_A08G0234	2504903	2509504	RNA binding (RRM/RBD/RNP motifs) family protein
Gh_A08G0235	2510990	2515659	zinc finger (CCCH-type/C3HC4-type RING finger) family protein
Gh_A08G0236	2541475	2544423	C2H2-type zinc finger family protein
Gh_A08G0237	2563405	2564770	ADP/ATP carrier 2
Gh_A08G0238	2574910	2576036	myb domain protein 83
Gh_A08G0239	2583856	2588086	MATE efflux family protein
Gh_A08G0240	2591644	2593776	CCCH-type zinc finger protein with ARM repeat domain
Gh_A08G0241	2601477	2607892	Pectin lyase-like superfamily protein
Gh_A08G0242	2608029	2613726	PHD finger family protein
Gh_A08G0243	2623134	2627201	cation/hydrogen exchanger 28
Gh_A08G0244	2629959	2632440	Ras-related small GTP-binding family protein
Gh_A08G0245	2634758	2637198	Protein kinase superfamily protein
Gh_A08G0246	2660210	2667367	eukaryotic translation initiation factor 4G
Gh_A08G0247	2688010	2688762	WUSCHEL related homeobox 13
Gh_A08G0248	2691404	2692951	Pentatricopeptide repeat (PPR) superfamily protein
Gh_A08G0249	2695978	2698456	casein kinase II beta chain 1
Gh_A08G0250	2716431	2720098	ELMO/CED-12 family protein
Gh_A08G0251	2722033	2724408	Hyaluronan / mRNA binding family
Gh_A08G0252	2726306	2740649	glycine-tRNA ligases
Gh_A08G0253	2742957	2748939	Leucine carboxyl methyltransferase
Gh_A08G0254	2755340	2760090	Proteasome component (PCI) domain protein
Gh_A08G0255	2765408	2771184	ARABIDILLO-1
Gh_A08G0256	2803317	2805011	Mitochondrial transcription termination factor family protein
Gh_A08G0257	2806941	2807346	
Gh_A08G0258	2810161	2816901	histone mono-ubiquitination 1
Gh_A08G0259	2818489	2822306	alpha/beta-Hydrolases superfamily protein
Gh_A08G0260	2857590	2861136	PLAC8 family protein
Gh_A08G0261	2870368	2871732	C2H2-type zinc finger family protein
Gh_A08G0262	2883316	2886088	structural molecules;transmembrane receptors;structural molecules

Gh_A08G0263	2890380	2904762	Ubiquitin-like superfamily protein
Gh_A08G0264	3115549	3115959	Histone superfamily protein
Gh_A08G0265	3127209	3128333	Plant protein of unknown function (DUF641)
Gh_A08G0266	3146256	3147563	UDP-D-glucuronate 4-epimerase 3
Gh_A08G0267	3167992	3169835	ribosomal protein S13A
Gh_A08G0268	3178366	3181686	Transducin/WD40 repeat-like superfamily protein
Gh_A08G0269	3182900	3185980	N-terminal nucleophile aminohydrolases (Ntn hydrolases) superfamily protein
Gh_A08G0270	3187399	3189285	
Gh_A08G0271	3191945	3193375	Plant protein of unknown function (DUF641)
Gh_A08G0272	3204951	3207998	SEC7-like guanine nucleotide exchange family protein
Gh_A08G0273	3210757	3216992	armadillo repeat kinesin 2
Gh_A08G0274	3233678	3237228	zinc finger protein-related
Gh_A08G0275	3257871	3271779	WD-40 repeat family protein / beige-related
Gh_A08G0276	3279449	3279888	HSP20-like chaperones superfamily protein
Gh_A08G0277	3280560	3282382	Uncharacterized conserved protein (DUF2358)
Gh_A08G0278	3288794	3291655	Phosphoenolpyruvate carboxylase family protein
Gh_A08G0279	3292699	3296407	Mechanosensitive ion channel protein
Gh_A08G0280	3300527	3300916	brassinosteroid-responsive RING-H2
Gh_A08G0281	3301898	3305153	
Gh_A08G0282	3305773	3307624	Cytidine/deoxycytidylate deaminase family protein
Gh_A08G0283	3308631	3313783	RNAhelicase-like 8
Gh_A08G0284	3317942	3321244	GATA type zinc finger transcription factor family protein
Gh_A08G0285	3329052	3334479	shaggy-like protein kinase 32
Gh_A08G0286	3338516	3342676	S-adenosyl-L-methionine-dependent methyltransferases superfamily protein
Gh_A08G0287	3347964	3350433	RING/U-box superfamily protein
Gh_A08G0288	3356954	3363390	Calcium-dependent lipid-binding (CaLB domain) family protein
Gh_A08G0289	3373820	3375633	Uncharacterized conserved protein (DUF2358)
Gh_A08G0290	3377148	3381375	POZ/BTB containin G-protein 1
Gh_A08G0291	3389857	3390123	
Gh_A08G0292	3394438	3397878	adenine nucleotide transporter 1
Gh_A08G0293	3406467	3412516	nucleotidyltransferases
Gh_A08G0294	3416108	3417027	cullin4
Gh_A08G0295	3420491	3424036	Plant protein 1589 of unknown function

Gh_A08G0296	3426388	3426732	lipid transfer protein 6
Gh_A08G0297	3429656	3430509	lipid transfer protein 3
Gh_A08G0298	3433656	3437049	
Gh_A08G0299	3462667	3463937	myb domain protein 3
Gh_A08G0300	3514107	3516533	RNA-binding (RRM/RBD/RNP motifs) family protein
Gh_A08G0301	3524119	3524487	SAUR-like auxin-responsive protein family
Gh_A08G0302	3531789	3532358	
Gh_A08G0303	3537709	3538327	60S acidic ribosomal protein family
Gh_A08G0304	3542559	3544671	nodulin MtN21 /EamA-like transporter family protein
Gh_A08G0305	3610336	3620681	
Gh_A08G0306	3634296	3635767	germin-like protein 10
Gh_A08G0307	3672758	3676697	Methylenetetrahydrofolate reductase family protein
Gh_A08G0308	3685263	3690564	Ypt/Rab-GAP domain of gyp1p superfamily protein
Gh_A08G0309	3692677	3693618	Transducin/WD40 repeat-like superfamily protein
Gh_A08G0310	3700006	3701115	S-adenosylmethionine decarboxylase
Gh_A08G0311	3716522	3717467	ADP-ribosylation factor-like A1A
Gh_A08G0312	3721707	3722522	plasmodesmata-located protein 7
Gh_A08G0313	3727697	3734144	histone acetyltransferase of the CBP family 1
Gh_A08G0314	3735274	3736171	allene oxide cyclase 4
Gh_A08G0315	3740104	3741001	allene oxide cyclase 4
Gh_A08G0316	3747184	3747922	allene oxide cyclase 4
Gh_A08G0317	3754097	3754995	allene oxide cyclase 4
Gh_A08G0318	3777730	3782151	NagB/RpiA/CoA transferase-like superfamily protein
Gh_A08G0319	3785555	3792467	decapping 5
Gh_A08G0320	3796826	3800722	
Gh_A08G0321	3807268	3807966	RING/U-box superfamily protein
Gh_A08G0322	3822952	3823650	RING/U-box superfamily protein
Gh_A08G0323	3826196	3826885	RING/U-box superfamily protein
Gh_A08G0324	3834410	3835108	RING/U-box superfamily protein
Gh_A08G0325	3869295	3870632	Pyridoxal phosphate (PLP)-dependent transferases superfamily protein
Gh_A08G0326	3872728	3877769	squamosa promoter binding protein-like 1
Gh_A08G0327	3894650	3896904	Protein kinase superfamily protein
Gh_A08G0328	3901534	3906517	

Gh_A08G0329	3909170	3911010	NAD(P)-binding Rossmann-fold superfamily protein
Gh_A08G0330	3922876	3924354	microsomal glutathione s-transferase; putative
Gh_A08G0331	3925270	3930468	polyamine oxidase 4
Gh_A08G0332	3937037	3937495	
Gh_A08G0333	3950261	3962258	ARM repeat superfamily protein
Gh_A08G0334	3962978	3973684	suppressor of abi3-5
Gh_A08G0335	3977922	3979781	AMP-dependent synthetase and ligase family protein
Gh_A08G0336	4028572	4032453	NAC domain containing protein 28
Gh_A08G0337	4039893	4046464	UDP-glucose pyrophosphorylase 2
Gh_A08G0338	4047562	4051774	
Gh_A08G0339	4058514	4059868	TPX2 (targeting protein for Xklp2) protein family
Gh_A08G0340	4079325	4079675	VQ motif-containing protein
Gh_A08G0341	4084747	4085991	Mitochondrial substrate carrier family protein
Gh_A08G0342	4112704	4113435	Pentatricopeptide repeat (PPR) superfamily protein
Gh_A08G0343	4124644	4125999	Eukaryotic aspartyl protease family protein
Gh_A08G0344	4155124	4157377	PLATZ transcription factor family protein
Gh_A08G0345	4189416	4190503	Peroxidase superfamily protein
Gh_A08G0346	4289296	4289624	Peroxidase superfamily protein
Gh_A08G0347	4289654	4290385	Peroxidase superfamily protein
Gh_A08G0348	4291053	4291523	polyubiquitin 10
Gh_A08G0349	4292436	4292999	Protein phosphatase 2C family protein
Gh_A08G0350	4314554	4316740	Heavy metal transport/detoxification superfamily protein
Gh_A08G0351	4319715	4320026	
Gh_A08G0352	4332893	4334060	ADP-ribosylation factor A1B
Gh_A08G0353	4334745	4339511	gibberellin 2-oxidase 6
Gh_A08G0354	4418569	4421115	Cyclophilin-like peptidyl-prolyl cis-trans isomerase family protein
Gh_A08G0355	4421923	4425991	P-loop containing nucleoside triphosphate hydrolases superfamily protein
Gh_A08G0356	4432478	4433161	Plant invertase/pectin methylesterase inhibitor superfamily protein
Gh_A08G0357	4434210	4437610	P-loop containing nucleoside triphosphate hydrolases superfamily protein
Gh_A08G0358	4439467	4440336	
Gh_A08G0359	4460644	4461072	basic region/leucine zipper motif 53
Gh_A08G0360	4466589	4468615	Ras-related small GTP-binding family protein
Gh_A08G0361	4474329	4477631	thioredoxin family protein

Gh_A08G0362	4482526	4484491	
Gh_A08G0363	4496475	4502681	CTP synthase family protein
Gh_A08G0364	4509441	4510034	zinc finger protein 6
Gh_A08G0365	4581247	4581615	Nucleotide-diphospho-sugar transferases superfamily protein
Gh_A08G0366	4594997	4595701	RING/U-box superfamily protein
Gh_A08G0367	4598987	4599262	RING/U-box superfamily protein
Gh_A08G0368	4620914	4621438	
Gh_A08G0369	4621897	4630862	
Gh_A08G0370	4700573	4707130	fumarase 1
Gh_A08G0371	4710904	4711874	ethylene-responsive element binding protein
Gh_A08G0372	4776320	4777555	receptor like protein 9
Gh_A08G0373	4780477	4780764	
Gh_A08G0374	4781358	4783154	Leucine-rich repeat transmembrane protein kinase
Gh_A08G0375	4788214	4795119	RELA/SPOT homolog 1
Gh_A08G0376	4801777	4802328	Plant invertase/pectin methylesterase inhibitor superfamily protein
Gh_A08G0377	4803638	4805425	ribosomal protein L18
Gh_A08G0378	4806419	4806790	senescence-associated gene 21
Gh_A08G0379	4816653	4818889	NADH-ubiquinone oxidoreductase-related
Gh_A08G0380	4820996	4822841	HSP20-like chaperones superfamily protein
Gh_A08G0381	4824829	4826836	Small nuclear ribonucleoprotein family protein
Locus II			
Gh_A10G0866	18790967	18794636	SEC14-like 12
Gh_A10G0867	18812916	18815114	MAP kinase 7
Gh_A10G0868	18872333	18875855	folate transporter 1
Gh_A10G0869	18876428	18884548	FAD-linked oxidases family protein
Gh_A10G0870	18885122	18887476	Methylthiotransferase
Gh_A10G0871	18887952	18889452	thioredoxin H-type 1
Gh_A10G0872	18965723	18967778	RTE1-homolog
Gh_A10G0873	18970654	18972268	nodulin MtN21 /EamA-like transporter family protein
Gh_A10G0874	18973640	18975584	Glutaredoxin family protein
Gh_A10G0875	19013571	19015097	ferulic acid 5-hydroxylase 1
Gh_A10G0876	19018573	19022698	PHD finger family protein / SWIB complex BAF60b domain-containing protein / GYF domain-containing protein
Gh_A10G0877	19056758	19057687	B-box zinc finger family protein

Gh_A10G0878	19058757	19059512	Lactoylglutathione lyase / glyoxalase I family protein
Gh_A10G0879	19091953	19092396	NAD(P)-binding Rossmann-fold superfamily protein
Gh_A10G0880	19094987	19096756	peptidylprolyl cis/trans isomerase; NIMA-interacting 1
Gh_A10G0881	19110559	19111783	Ribosomal protein L2 family
Gh_A10G0882	19121677	19121991	SAUR-like auxin-responsive protein family
Gh_A10G0883	19126886	19129630	zinc finger (Ran-binding) family protein
Gh_A10G0884	19135722	19136784	Homeodomain-like superfamily protein
Gh_A10G0885	19144134	19144502	
Gh_A10G0886	19193297	19197076	calcium-dependent protein kinase 28
Gh_A10G0887	19201305	19204587	RADIATION SENSITIVE 17
Gh_A10G0888	19268072	19269237	plasma membrane intrinsic protein 3
Gh_A10G0889	19271732	19272266	
Gh_A10G0890	19284868	19290141	Golgi-localized GRIP domain-containing protein
Gh_A10G0891	19295716	19301610	Phosphoinositide phosphatase family protein
Gh_A10G0892	19305188	19306760	Protein kinase superfamily protein
Gh_A10G0893	19341907	19342776	Vacuolar iron transporter (VIT) family protein
Gh_A10G0894	19344371	19346562	Ribosomal protein S4
Gh_A10G0895	19360060	19362678	Pentatricopeptide repeat (PPR) superfamily protein
Gh_A10G0896	19401601	19403964	Protein of unknown function (DUF1666)
Gh_A10G0897	19430317	19430741	
Gh_A10G0898	19667028	19667381	
Gh_A10G0899	19726691	19731839	UDP-sugar pyrophosphorylase
Locus III			
Gh_A10G1126	57194822	57196222	Protein kinase superfamily protein
Gh_A10G1127	57205771	57207386	Protein kinase superfamily protein
Gh_A10G1128	57237749	57239146	Protein kinase superfamily protein
Gh_A10G1129	57261653	57264212	receptor like protein 35
Gh_A10G1130	57322109	57326044	Leucine-rich receptor-like protein kinase family protein
Gh_A10G1131	57418967	57420707	S-adenosyl-L-homocysteine hydrolase
Gh_A10G1132	57472895	57475219	serine hydroxymethyltransferase 4
Gh_A10G1133	57484904	57488166	Werner syndrome-like exonuclease
Gh_A10G1134	57669306	57670670	UDP-D-glucuronate 4-epimerase 6
Gh_A10G1135	57697584	57701455	methylenetetrahydrofolate reductase 2

Gh_A10G1136	57732952	57740376	
Gh_A10G1137	57847838	57851848	RNA recognition motif (RRM)-containing protein
Gh_A10G1138	57889117	57909260	ARM repeat superfamily protein
Gh_A10G1139	57936838	57938115	
Gh_A10G1140	57988795	57990291	ralf-like 24
Gh_A10G1141	58046988	58048157	Exostosin family protein
Gh_A10G1142	58054211	58054426	
Gh_A10G1143	58134995	58151527	AMP-dependent synthetase and ligase family protein
Gh_A10G1144	58305761	58308406	selenium-binding protein 2
Gh_A10G1145	58325987	58326912	
Gh_A10G1146	58356513	58356851	ralf-like 32
Gh_A10G1147	58358312	58361580	Putative methyltransferase family protein
Locus IV			
Gh_D07G0847	11059477	11062522	Ypt/Rab-GAP domain of gyp1p superfamily protein
Gh_D07G0848	11064085	11067939	ankyrin repeat family protein
Gh_D07G0849	11068594	11072456	fimbrin-like protein 2
Gh_D07G0850	11076746	11077543	FASCICLIN-like arabinoogalactan 7
Gh_D07G0851	11086362	11089049	Immunoglobulin E-set superfamily protein
Gh_D07G0852	11110068	11113249	RNA-binding (RRM/RBD/RNP motifs) family protein
Gh_D07G0853	11115279	11116960	RNA-binding (RRM/RBD/RNP motifs) family protein
Gh_D07G0854	11121945	11128589	Kinase-related protein of unknown function (DUF1296)
Gh_D07G0855	11128727	11129101	
Gh_D07G0856	11138242	11140228	RNA-binding (RRM/RBD/RNP motifs) family protein
Gh_D07G0857	11146245	11150681	AMP-dependent synthetase and ligase family protein
Gh_D07G0858	11153344	11156901	
Gh_D07G0859	11211102	11211575	F-box family protein
Gh_D07G0860	11214658	11216709	DNAJ heat shock N-terminal domain-containing protein
Gh_D07G0861	11221175	11223955	Sterile alpha motif (SAM) domain-containing protein
Gh_D07G0862	11227186	11227542	
Gh_D07G0863	11229973	11235506	Peptidase M28 family protein
Gh_D07G0864	11241244	11243162	Protein of unknown function (DUF1423)
Gh_D07G0865	11244287	11245975	
Gh_D07G0866	11248401	11248853	RING/U-box superfamily protein

Gh_D07G0867	11252477	11254842	CONSTANS-like 9
Gh_D07G0868	11279915	11282977	Homeodomain-like protein with RING/FYVE/PHD-type zinc finger domain
Gh_D07G0869	11283749	11285716	Endomembrane protein 70 protein family
Gh_D07G0870	11286463	11288875	HhH-GPD base excision DNA repair family protein
Gh_D07G0871	11290845	11293966	glyoxylate reductase 1
Gh_D07G0872	11297101	11297334	
Gh_D07G0873	11299853	11301672	
Gh_D07G0874	11364649	11367705	Leucine-rich repeat receptor-like protein kinase family protein
Gh_D07G0875	11370927	11373183	P-loop containing nucleoside triphosphate hydrolases superfamily protein
Gh_D07G0876	11383782	11387128	formin homology 1
Gh_D07G0877	11400246	11408097	FRIGIDA-like protein
Gh_D07G0878	11409520	11410414	photosystem I subunit l
Gh_D07G0879	11422845	11428480	Glutamate receptor family protein
Gh_D07G0880	11432436	11437676	Glutamate receptor family protein
Gh_D07G0881	11441079	11446687	glutamate receptor 1.3
Gh_D07G0882	11467358	11472903	glutamate receptor 1.4
Gh_D07G0883	11516557	11516844	
Gh_D07G0884	11531199	11532233	
Gh_D07G0885	11534967	11539318	SKU5 similar 3
Gh_D07G0886	11559013	11559607	Lactoylglutathione lyase / glyoxalase I family protein
Gh_D07G0887	11562508	11563176	YGGT family protein
Gh_D07G0888	11563692	11563940	
Gh_D07G0889	11570347	11571158	
Gh_D07G0890	11575782	11579222	asparaginyl-tRNA synthetase 2
Gh_D07G0891	11579684	11587946	lipase class 3 family protein
Gh_D07G0892	11594283	11596819	Auxin-responsive family protein
Gh_D07G0893	11600674	11602101	Ribosomal protein L32e
Gh_D07G0894	11604772	11606417	Ribosomal protein L32e
Gh_D07G0895	11621163	11622802	Ribosomal protein L32e
Gh_D07G0896	11624821	11625726	Protein of unknown function (DUF506)
Gh_D07G0897	11648262	11651781	basic helix-loop-helix (bHLH) DNA-binding superfamily protein
Gh_D07G0898	11659953	11663346	FKBP-type peptidyl-prolyl cis-trans isomerase family protein
Gh_D07G0899	11682580	11683743	Pentatricopeptide repeat (PPR) superfamily protein

Gh_D07G0900	11686977	11687426	
Gh_D07G0901	11705043	11706491	Oxidoreductase; zinc-binding dehydrogenase family protein
Gh_D07G0902	11706712	11707305	Oxidoreductase; zinc-binding dehydrogenase family protein
Gh_D07G0903	11715091	11718185	Oxidoreductase; zinc-binding dehydrogenase family protein
Gh_D07G0904	11720140	11723657	Oxidoreductase; zinc-binding dehydrogenase family protein
Gh_D07G0905	11726619	11729828	Oxidoreductase; zinc-binding dehydrogenase family protein
Gh_D07G0906	11731919	11734498	Oxidoreductase; zinc-binding dehydrogenase family protein
Gh_D07G0907	11743446	11744202	
Gh_D07G0908	11744744	11748774	Cyclin family protein
Gh_D07G0909	11751396	11753851	Tetratricopeptide repeat (TPR)-like superfamily protein
Gh_D07G0910	11776584	11779939	cytochrome P450; family 82; subfamily G; polypeptide 1
Gh_D07G0911	11816943	11819051	cytochrome P450; family 82; subfamily G; polypeptide 1
Gh_D07G0912	11863897	11866672	cytochrome P450; family 82; subfamily G; polypeptide 1
Gh_D07G0913	11891038	11893713	Nucleotide-diphospho-sugar transferases superfamily protein
Gh_D07G0914	11924534	11925631	
Gh_D07G0915	11965503	11966135	RING/U-box superfamily protein
Gh_D07G0916	11973296	11975830	Integrase-type DNA-binding superfamily protein
Gh_D07G0917	11977663	11979305	Mitochondrial import inner membrane translocase subunit Tim17/Tim22/Tim23 family protein
Gh_D07G0918	11988503	11994329	RNA binding (RRM/RBD/RNP motifs) family protein
Gh_D07G0919	11998289	12000939	DNA repair (Rad51) family protein
Gh_D07G0920	12050539	12053123	transportin 1
Gh_D07G0921	12061318	12061524	
Gh_D07G0922	12082767	12083318	
Gh_D07G0923	12083661	12088342	Protein of unknown function (DUF604)
Gh_D07G0924	12099291	12101164	BTB/POZ domain-containing protein
Gh_D07G0925	12101512	12102438	
Gh_D07G0926	12115528	12116219	Embryo-specific protein 3; (ATS3)
Gh_D07G0927	12127346	12127657	
Gh_D07G0928	12164885	12168659	ferric reductase-like transmembrane component family protein
Gh_D07G0929	12217497	12219579	CCT motif family protein
Gh_D07G0930	12230924	12231382	HSP20-like chaperones superfamily protein
Gh_D07G0931	12268623	12273213	ER-type Ca2+-ATPase 1
Gh_D07G0932	12311508	12311963	

Gh_D07G0933	12314882	12317233	hercules receptor kinase 1
Gh_D07G0934	12336129	12338375	Ankyrin repeat family protein
Gh_D07G0935	12394627	12396430	
Gh_D07G0936	12410514	12411914	3-ketoacyl-CoA synthase 12
Gh_D07G0937	12444178	12446010	exocyst subunit exo70 family protein H7
Gh_D07G0938	12470559	12471186	Heavy metal transport/detoxification superfamily protein
Gh_D07G0939	12484619	12485599	ribosomal protein L23AA
Gh_D07G0940	12491403	12492431	Protein of unknown function (DUF1635)
Gh_D07G0941	12521294	12522240	myb domain protein 48
Gh_D07G0942	12583984	12596687	Domain of unknown function (DUF966)
Gh_D07G0943	12610365	12612298	PIF / Ping-Pong family of plant transposases
Gh_D07G0944	12614692	12615718	Ribosomal protein S8 family protein
Gh_D07G0945	12635197	12635652	Histone superfamily protein
Gh_D07G0946	12636321	12636641	
Gh_D07G0947	12645084	12647125	RNA-binding (RRM/RBD/RNP motifs) family protein
Gh_D07G0948	12663993	12666915	
Gh_D07G0949	12683145	12683754	dessication-induced 1VOC superfamily protein
Gh_D07G0950	12684290	12686300	ubiquitin-conjugating enzyme 13
Gh_D07G0951	12710159	12710999	WUSCHEL related homeobox 2
Gh_D07G0952	12714329	12714589	
Gh_D07G0953	12719410	12720306	
Gh_D07G0954	12761737	12762883	Dof-type zinc finger DNA-binding family protein
Gh_D07G0955	12853446	12855057	plasmodesmata-located protein 7
Gh_D07G0956	12910767	12914844	plant U-box 13
Gh_D07G0957	12963812	12965086	HXXXD-type acyl-transferase family protein
Gh_D07G0958	13006266	13006550	
Gh_D07G0959	13010269	13012514	51 kDa subunit of complex I
Gh_D07G0960	13014095	13019403	SEC12P-like 2 protein
Gh_D07G0961	13022634	13023116	heat shock protein 18.2
Gh_D07G0962	13051596	13053994	XB3 ortholog 1 in Arabidopsis thaliana
Gh_D07G0963	13054865	13056609	
Gh_D07G0964	13058943	13059452	
Gh_D07G0965	13073468	13075112	actin 1

Gh_D07G0966	13075827	13077517	Fasciclin-like arabinogalactan family protein
Gh_D07G0967	13086927	13088037	Tetratricopeptide repeat (TPR)-like superfamily protein
Gh_D07G0968	13088050	13088976	Tetratricopeptide repeat (TPR)-like superfamily protein
Gh_D07G0969	13089370	13090399	outer plastid envelope protein 16-1
Gh_D07G0970	13093746	13094732	CAX interacting protein 4
Gh_D07G0971	13111450	13115948	arginine methyltransferase 11
Gh_D07G0972	13120467	13122276	beta-ureidopropionase
Gh_D07G0973	13149189	13149569	flowering promoting factor 1
Gh_D07G0974	13176766	13177922	expansin A4
Gh_D07G0975	13206205	13208180	Exostosin family protein
Gh_D07G0976	13209417	13211294	
Gh_D07G0977	13246821	13249124	SCARECROW-like 14
Gh_D07G0978	13259905	13262262	SCARECROW-like 14
Gh_D07G0979	13271646	13275374	cytochrome P450; family 707; subfamily A; polypeptide 2
Gh_D07G0980	13306323	13306565	
Gh_D07G0981	13311248	13312333	zinc finger (C3HC4-type RING finger) family protein
Gh_D07G0982	13334635	13340518	pumilio 1
Gh_D07G0983	13341278	13341903	
Gh_D07G0984	13356669	13357685	Homeodomain-like superfamily protein
Gh_D07G0985	13382126	13386341	translocon at the outer envelope membrane of chloroplasts 75-III
Gh_D07G0986	13386938	13388427	
Gh_D07G0987	13445304	13445657	
Gh_D07G0988	13446340	13449847	Transcription factor IIA; alpha/beta subunit
Gh_D07G0989	13451168	13453229	plastid transcriptionally active 16
Gh_D07G0990	13464229	13464774	telomerase activator1
Gh_D07G0991	13478064	13480612	type one serine/threonine protein phosphatase 2
Gh_D07G0992	13487032	13488138	metal tolerance protein B1
Gh_D07G0993	13488652	13490894	RAB GTPase homolog A2B
Gh_D07G0994	13499139	13499339	HSP20-like chaperones superfamily protein
Gh_D07G0995	13501942	13505014	UDP-Glycosyltransferase superfamily protein
Gh_D07G0996	13505993	13512771	UDP-glucosyl transferase 76E2
Gh_D07G0997	13538368	13539350	
Gh_D07G0998	13541969	13543027	maternal effect embryo arrest 60

Gh_D07G0999	13545115	13546479	ARM repeat superfamily protein
Gh_D07G1000	13548031	13548306	zinc ion binding;nucleic acid binding
Gh_D07G1001	13595815	13597273	UDP-Glycosyltransferase superfamily protein
Gh_D07G1002	13616787	13618240	UDP-Glycosyltransferase superfamily protein
Gh_D07G1003	13667683	13669154	UDP-glucosyl transferase 76E2
Gh_D07G1004	13707278	13710741	hAT dimerisation domain-containing protein
Gh_D07G1005	13734451	13734714	HSP20-like chaperones superfamily protein
Gh_D07G1006	13767600	13799786	Protein kinase protein with tetratricopeptide repeat domain
Gh_D07G1007	13800642	13802530	
Gh_D07G1008	13817441	13820373	uncoupling protein 2
Gh_D07G1009	13823831	13825195	ribonuclease P family protein / Rpp14 family protein
Gh_D07G1010	13830083	13833558	calmodulin-binding receptor-like cytoplasmic kinase 1
Gh_D07G1011	13834491	13836230	Protein of unknown function (DUF740)
Gh_D07G1012	13870084	13870458	josephin protein-related
Gh_D07G1013	13900980	13902524	cytochrome P450; family 86; subfamily A; polypeptide 1
Gh_D07G1014	13930669	13931820	zinc finger (C2H2 type) family protein
Gh_D07G1015	13962635	13964989	myb domain protein 119
Locus V			
Gh_D12G1548	46359064	46362409	heat shock transcription factor A8
Gh_D12G1549	46366732	46369472	semialdehyde dehydrogenase family protein
Gh_D12G1550	46371659	46386664	ARF-GAP domain 4
Gh_D12G1551	46449382	46456812	Protein of unknown function (DUF3755)
Gh_D12G1552	46459203	46462594	Leucine-rich repeat protein kinase family protein
Gh_D12G1553	46471427	46475035	glutaredoxin-related
Gh_D12G1554	46501249	46504186	NAD(P)-linked oxidoreductase superfamily protein
Gh_D12G1555	46506145	46510135	myo-inositol oxygenase 1
Gh_D12G1556	46513482	46522251	tobamovirus multiplication protein 3
Gh_D12G1557	46550083	46555842	Calcium-dependent lipid-binding (CaLB domain) family protein
Gh_D12G1558	46556735	46557196	
Gh_D12G1559	46570546	46572226	Peroxidase superfamily protein
Gh_D12G1560	46584783	46585541	Ubiquitin-like superfamily protein
Gh_D12G1561	46589401	46589795	Homeodomain-like superfamily protein
Gh_D12G1562	46600430	46601008	Late embryogenesis abundant (LEA) hydroxyproline-rich glycoprotein family

Gh_D12G1563	46679355	46683260	homeodomain GLABROUS 2
Gh_D12G1564	46691003	46724587	Transmembrane protein 97; predicted
Gh_D12G1565	46733863	46734451	Transmembrane protein 97; predicted
Gh_D12G1566	46736441	46736753	
Gh_D12G1567	46740003	46741391	Ras-related small GTP-binding family protein
Gh_D12G1568	46742388	46746298	glutamate receptor 3.4
Gh_D12G1569	46755414	46762909	aconitase 3
Gh_D12G1570	46766024	46766380	
Gh_D12G1571	46780267	46782207	Ubiquitin-like superfamily protein
Gh_D12G1572	46782934	46799934	ARM repeat superfamily protein
Gh_D12G1573	46807922	46808941	
Gh_D12G1574	46821972	46826338	Calcium-dependent phosphotriesterase superfamily protein
Gh_D12G1575	46828004	46829597	Tetratricopeptide repeat (TPR)-like superfamily protein
Gh_D12G1576	46839381	46844209	Phosphoribulokinase / Uridine kinase family
Gh_D12G1577	46856738	46864694	Peroxidase superfamily protein
Gh_D12G1578	46884467	46884780	phytosulfokine 4 precursor
Gh_D12G1579	46892795	46893385	basic leucine-zipper 58
Gh_D12G1580	46915126	46922155	Armadillo/beta-catenin-like repeat family protein
Gh_D12G1581	46940839	46949201	male gametophyte defective 3
Gh_D12G1582	46991591	46993522	phosphoinositide 4-kinase gamma 7
Gh_D12G1583	47029047	47031500	BES1-interacting Myc-like protein 2
Gh_D12G1584	47031860	47036942	Prolyl oligopeptidase family protein
Gh_D12G1585	47038016	47039444	BSD domain-containing protein
Gh_D12G1586	47040783	47042267	18S pre-ribosomal assembly protein gar2-related
Gh_D12G1587	47063564	47066044	ACT domain repeat 4
Gh_D12G1588	47081921	47082130	
Gh_D12G1589	47092661	47095212	
Gh_D12G1590	47097052	47104558	DNA binding
Gh_D12G1591	47139959	47141614	cytochrome P450; family 78; subfamily A; polypeptide 5
Gh_D12G1592	47149550	47153952	RNA helicase family protein
Gh_D12G1593	47158178	47160474	Major facilitator superfamily protein
Gh_D12G1594	47168597	47175885	Major facilitator superfamily protein
Gh_D12G1595	47176357	47178244	Dihydropterin pyrophosphokinase / Dihydropteroate synthase

Gh_D12G1596	47180300	47183605	fructokinase-like 2
Gh_D12G1597	47186333	47195143	Protein kinase superfamily protein
Gh_D12G1598	47272604	47276990	Asparagine synthase family protein
Gh_D12G1599	47282708	47284592	ABI five binding protein 2
Gh_D12G1600	47285223	47288606	Leucine-rich repeat transmembrane protein kinase
Gh_D12G1601	47293065	47296525	
Gh_D12G1602	47312753	47315974	magnesium transporter 3
Gh_D12G1603	47316258	47320540	Purple acid phosphatases superfamily protein
Gh_D12G1604	47322792	47326984	Protein of unknown function; DUF647
Gh_D12G1605	47327647	47328398	SPFH/Band 7/PHB domain-containing membrane-associated protein family
Gh_D12G1606	47334171	47334779	SPFH/Band 7/PHB domain-containing membrane-associated protein family
Gh_D12G1607	47343953	47352801	alpha/beta-Hydrolases superfamily protein
Gh_D12G1608	47359014	47361422	high mobility group A5
Gh_D12G1609	47374759	47375919	plasmodesmata callose-binding protein 3
Gh_D12G1610	47378322	47382834	Tetratricopeptide repeat (TPR)-like superfamily protein
Gh_D12G1611	47411421	47414852	QUASIMODO2 LIKE 2
Gh_D12G1612	47445793	47447235	UDP-glucose 6-dehydrogenase family protein
Gh_D12G1613	47457227	47462170	C2H2-like zinc finger protein
Gh_D12G1614	47470423	47472824	WRKY DNA-binding protein 57
Gh_D12G1615	47480036	47482078	homologue of bacterial MinE 1
Gh_D12G1616	47510862	47512730	Plant protein of unknown function (DUF863)
Gh_D12G1617	47514901	47517093	Tetratricopeptide repeat (TPR)-like superfamily protein
Gh_D12G1618	47517741	47518307	
Gh_D12G1619	47521801	47525150	Transducin/WD40 repeat-like superfamily protein
Gh_D12G1620	47525959	47526983	eukaryotic elongation factor 5A-1
Gh_D12G1621	47527458	47530331	Protein of unknown function (DUF1336)
Gh_D12G1622	47538053	47539099	related to AP2 11
Gh_D12G1623	47544233	47545654	
Gh_D12G1624	47547992	47549260	non-ATPase subunit 9
Gh_D12G1625	47550155	47551929	Vesicle transport v-SNARE family protein
Gh_D12G1626	47560907	47565363	VH1-interacting kinase
Gh_D12G1627	47572421	47576158	O-fucosyltransferase family protein
Gh_D12G1628	47601907	47603428	myb domain protein 16 (MYB25-like)

Gh_D12G1629	47649966	47650301	
Gh_D12G1630	47650316	47651139	myb domain protein 106
Gh_D12G1631	47755149	47755385	mucin-related
